# Supplementary material for: A biocatalytic hydroxylation-enabled unified approach to C19-hydroxylated steroids
Source: Nat Commun. 2019 Jul 29;10:3378. doi: 10.1038/s41467-019-11344-0 (PMC6662754; doi:10.1038/s41467-019-11344-0)
Supplement: Supplementary file 1 — Supplementary Information [file 41467_2019_11344_MOESM1_ESM.pdf]

Supplementary Information for

A Biocatalytic Hydroxylation-Enabled Unified Approach to C19-hydroxylated Steroids

Wang et al.

Table of Contents

Supplementary Figure 1-81	3-48
Supplementary Table 1-10	49-56
Supplementary methods	57
Supplementary notes 1-19	58-69
Supplementary References	70

Supplementary Figures

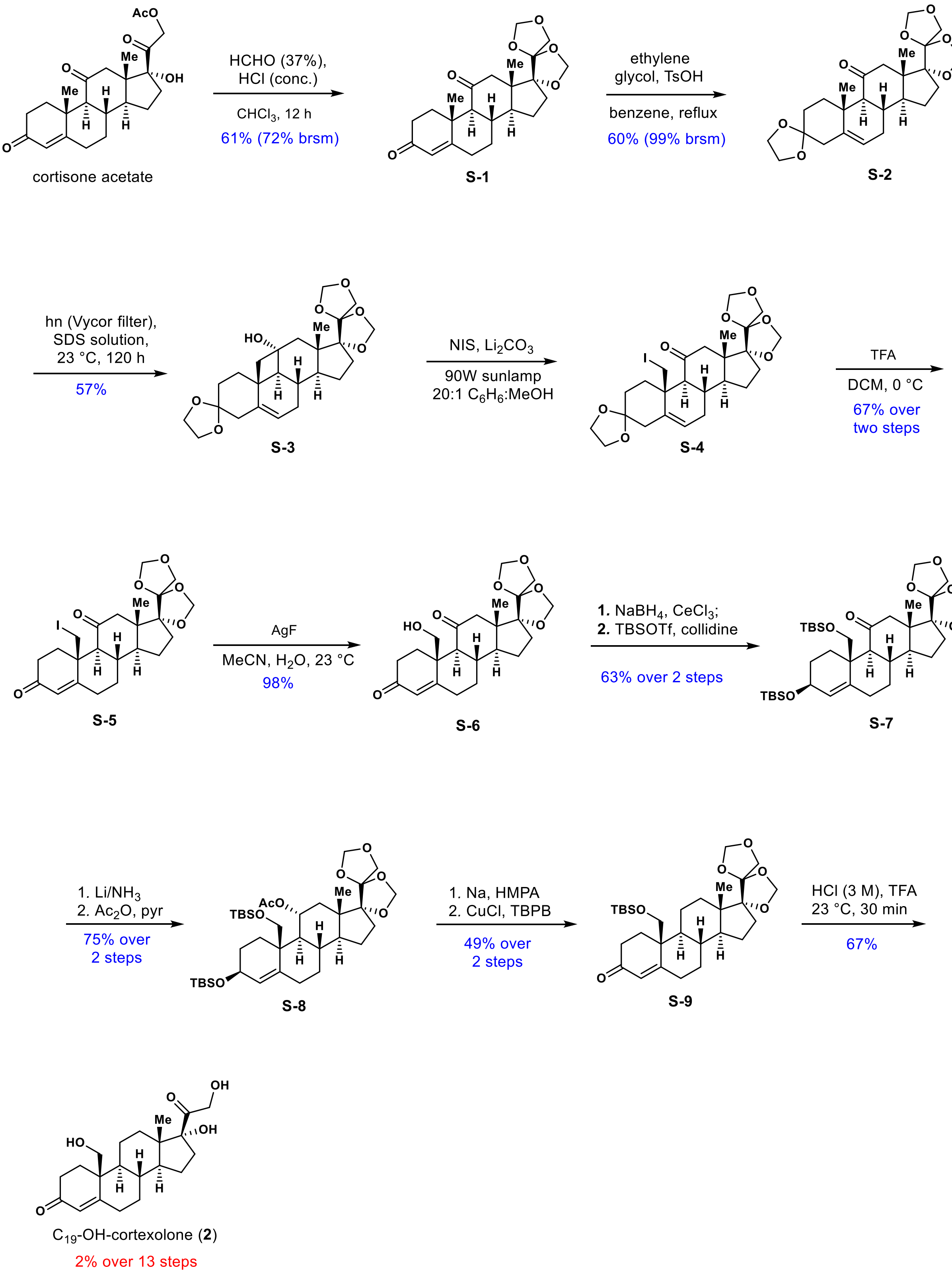

Supplementary Figure 1. Reported synthetic route of **2**[1]

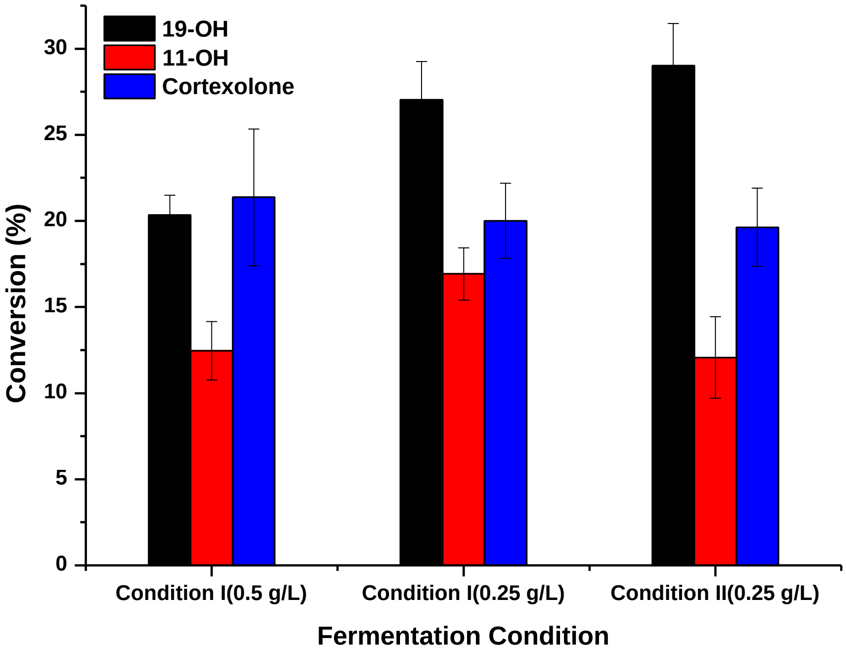

Supplementary Figure 2. Influence of different fermentation conditions on the conversation of cortexolone by *T. cucumeris*. (Condition I: fermentation with solid media culture; Condition II: fermentation with broth culture, n=2 biologically independent experiments). Error bar indicates the range of the values achieved by two independent experiments. Source data are provided as a Source Data file.

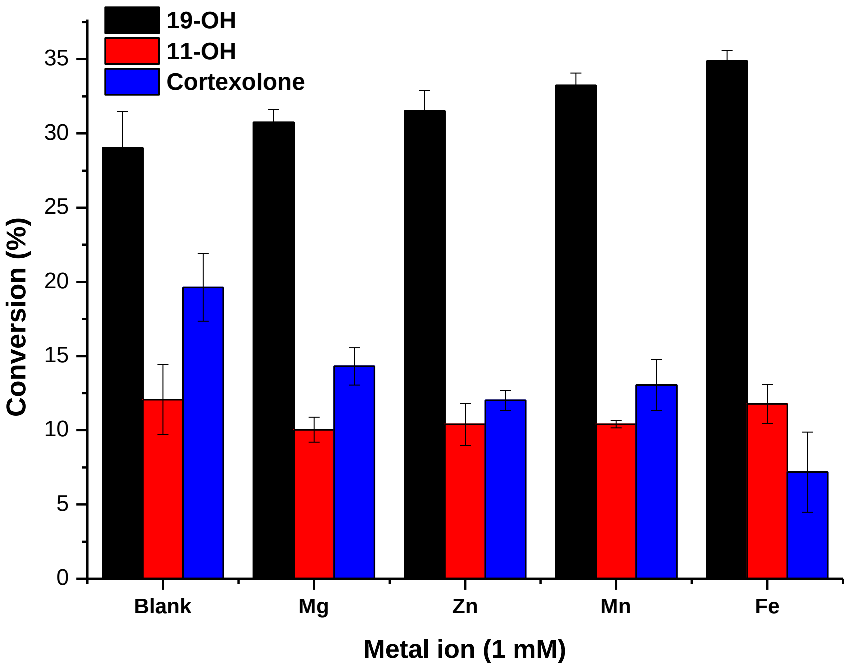

Supplementary Figure 3. Influence of different metal ions on the conversation of cortexolone by *T. cucumeris*. N=2 biologically independent experiments. Error bar indicates the range of the values achieved by two independent experiments. Source data are provided as a Source Data file.

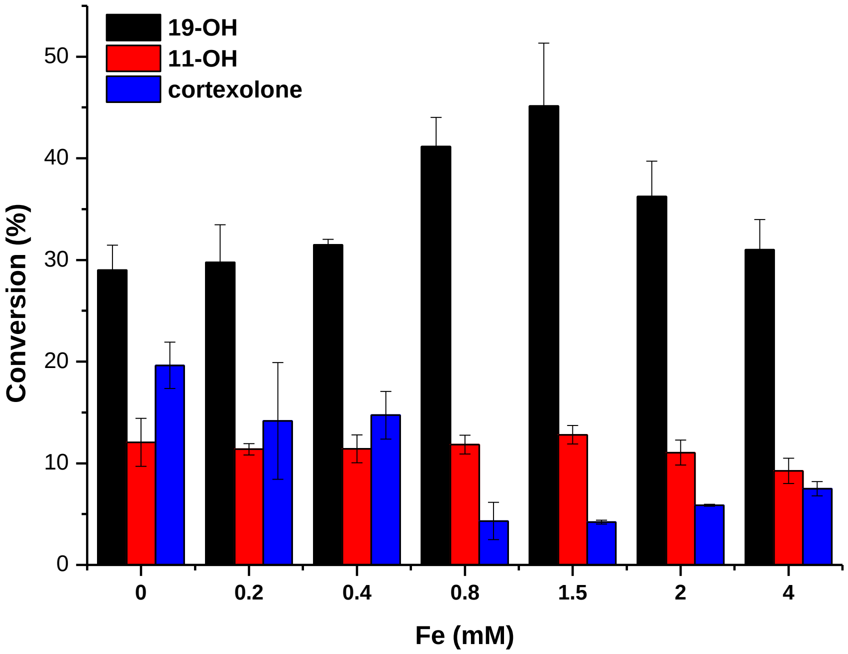

Supplementary Figure 4. Influence of different concentrations of Fe^{2+} on the conversation of cortexolone by *T. cucumeris*. N=2 biologically independent experiments. Error bar indicates the range of the values achieved by two independent experiments. Source data are provided as a Source Data file.

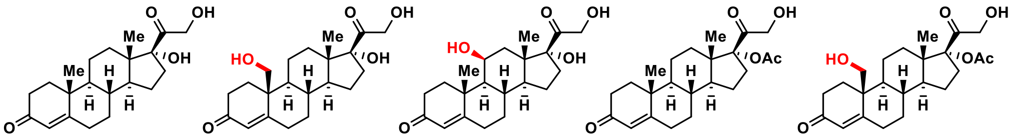

1 2 3 6 7

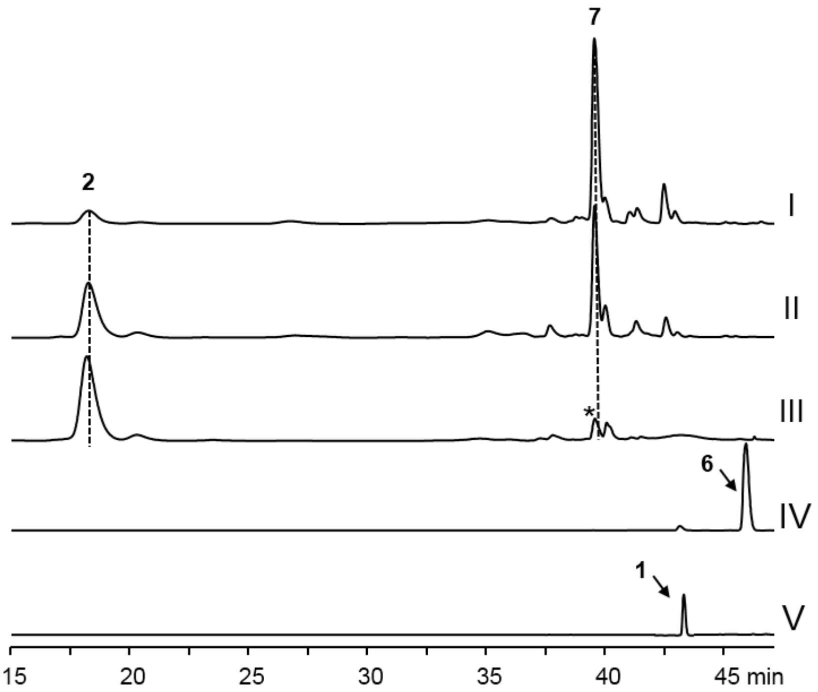

Supplementary Figure 5. Production of **7** in *T. cucumeris* from the first to third day. (I) the first day; (II) the second day; (III) the third day; (IV) standard 17-Ac cortexolone (**6**); (V) standard cortexolone (**1**). (*Mixture of **3** and **7** with a ratio of 5:1).

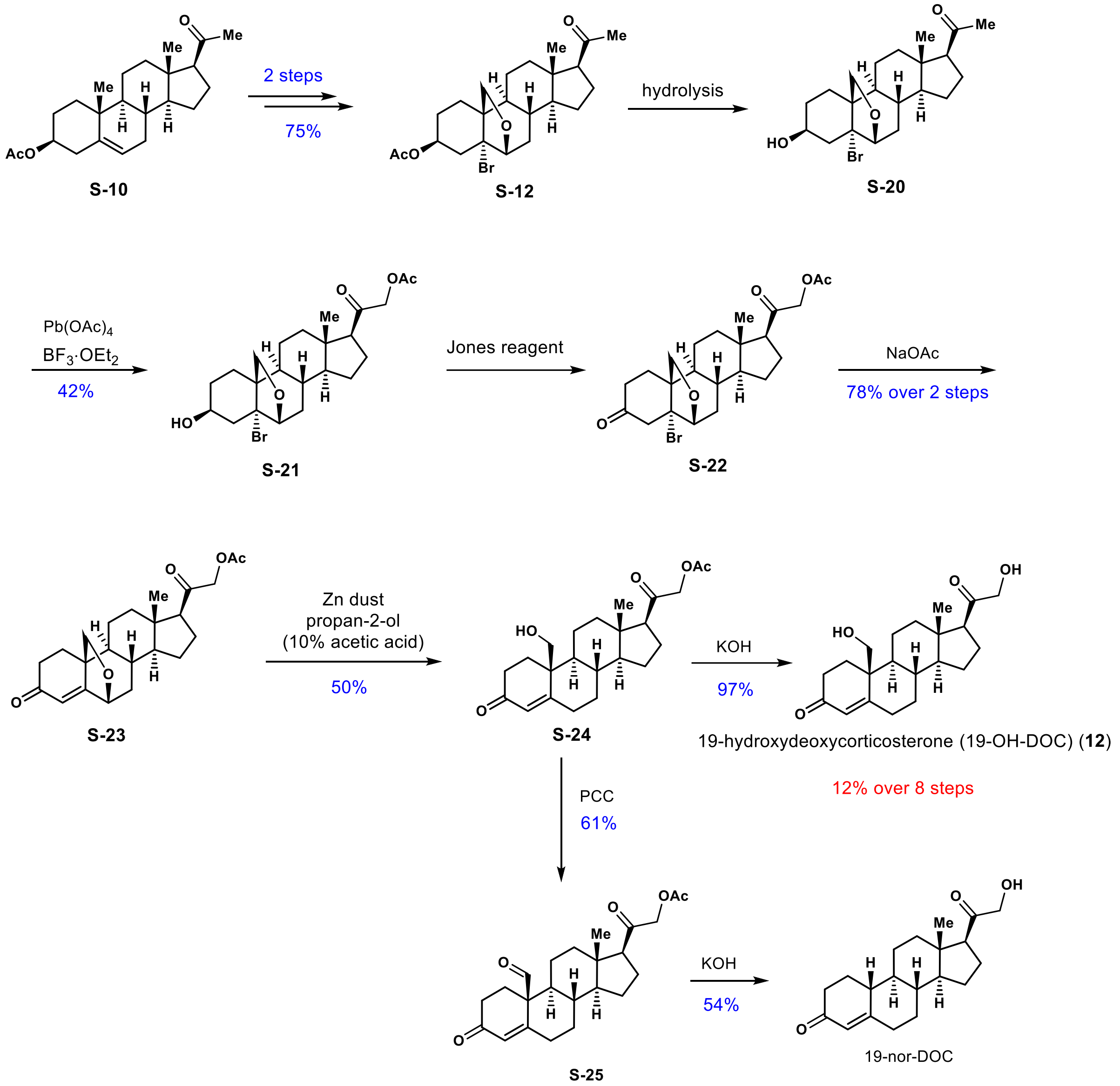

Supplementary Figure 6. Reported synthetic route A of **12** and its application to 19-nor-DOC synthesis[2]

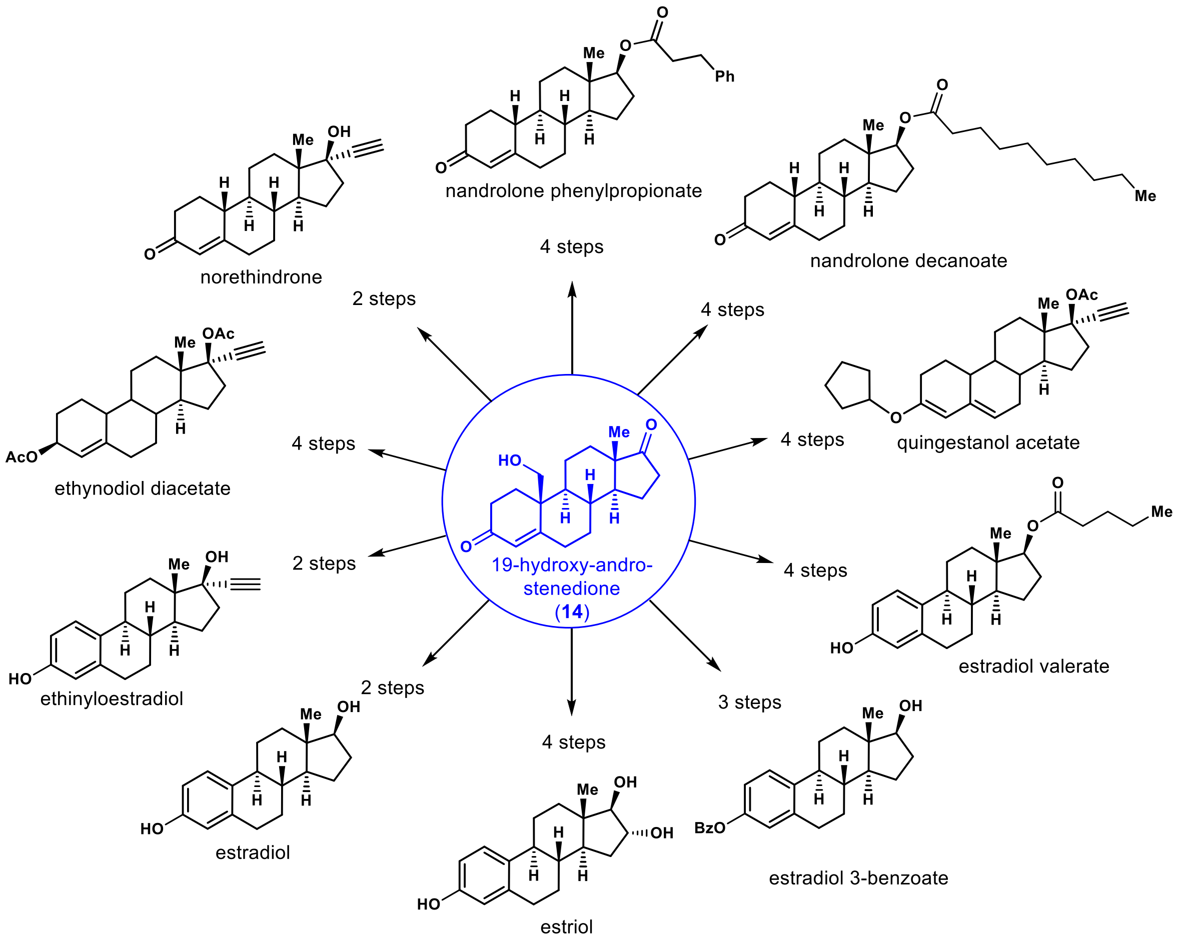

Supplementary Figure 7. 14 as key intermediate for synthesis of ten 19-nor steroid drugs[3]

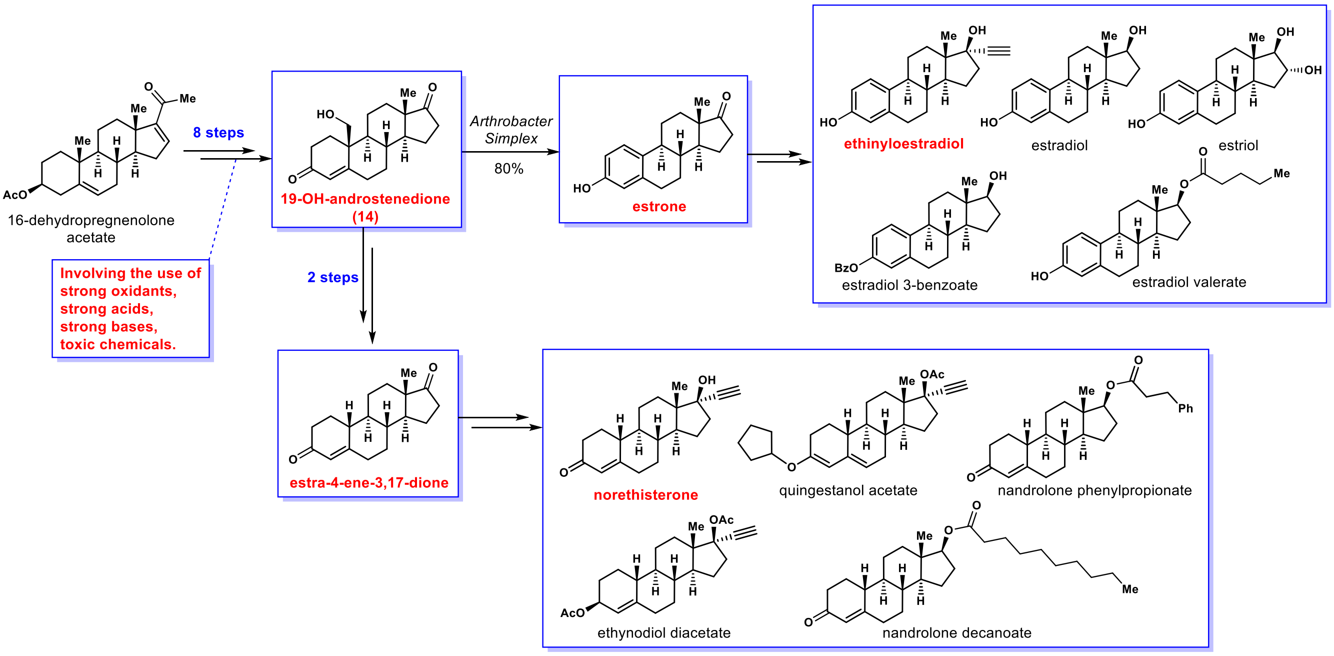

Supplementary Figure 8. Process for manufacturing 19-OH-androstenedione (**14**) and related 19-nor steroidal medicines in industry.

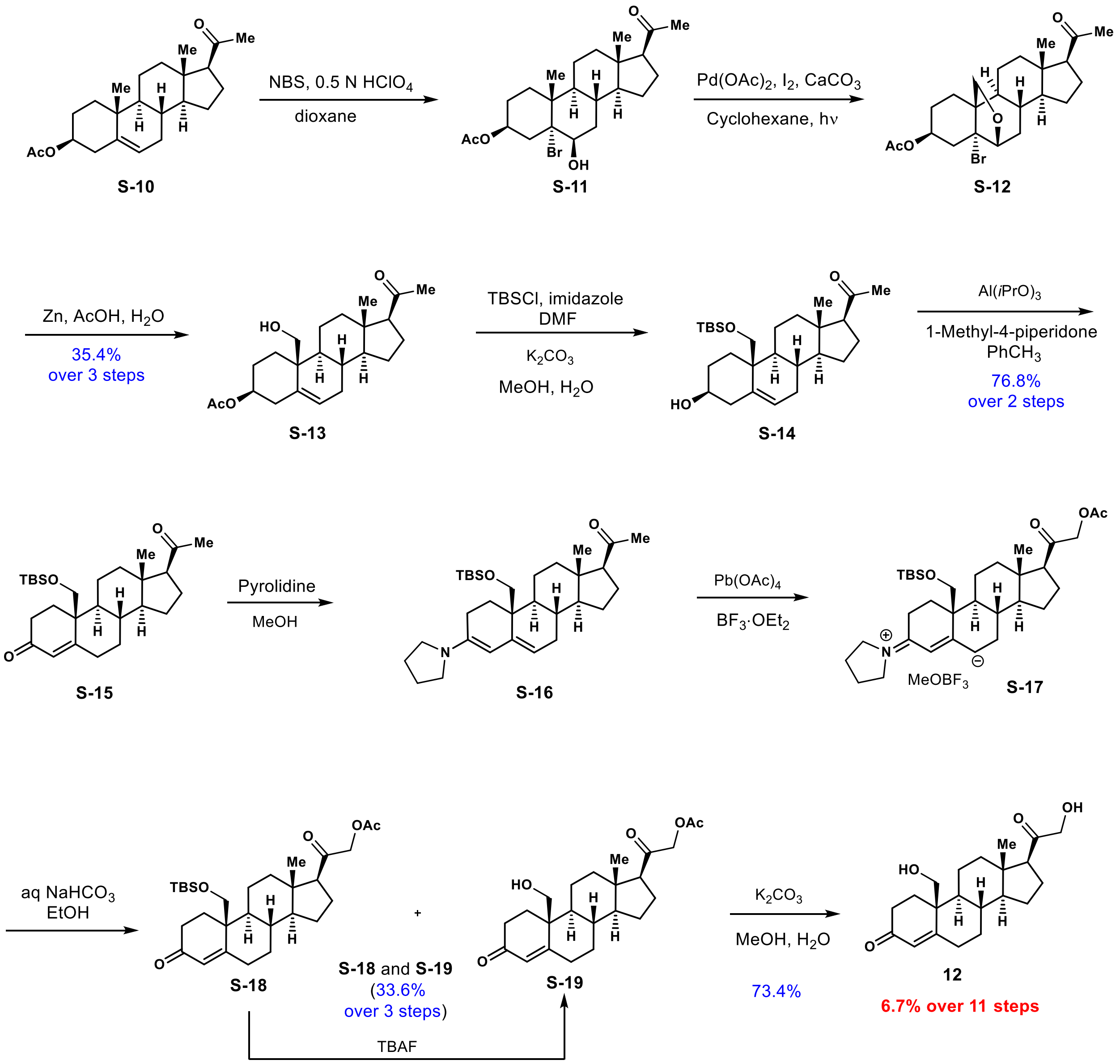

Supplementary Figure 9. Reported synthetic route B of **12**[4]

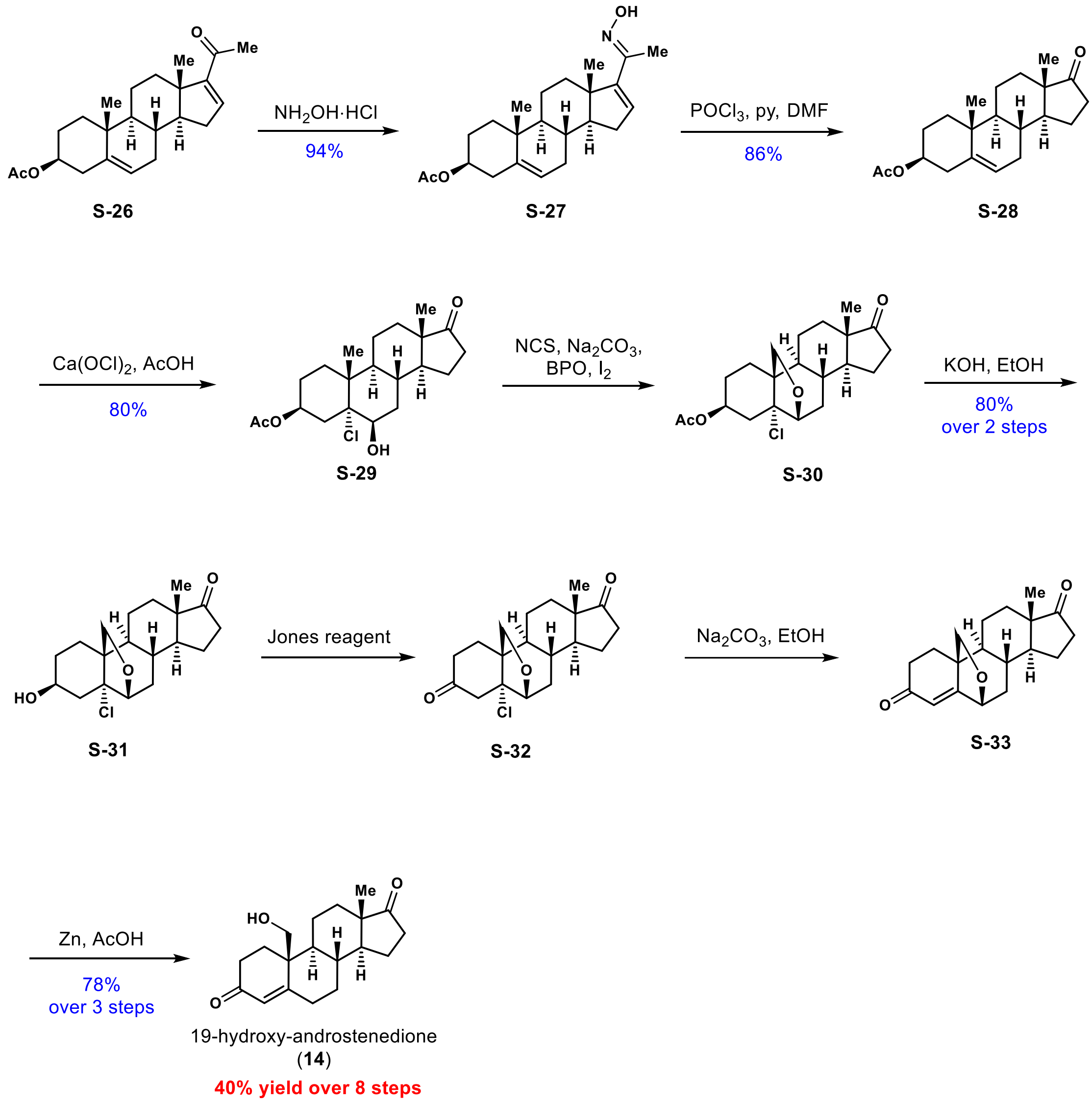

Supplementary Figure 10. Reported synthetic route A of **14**[3]

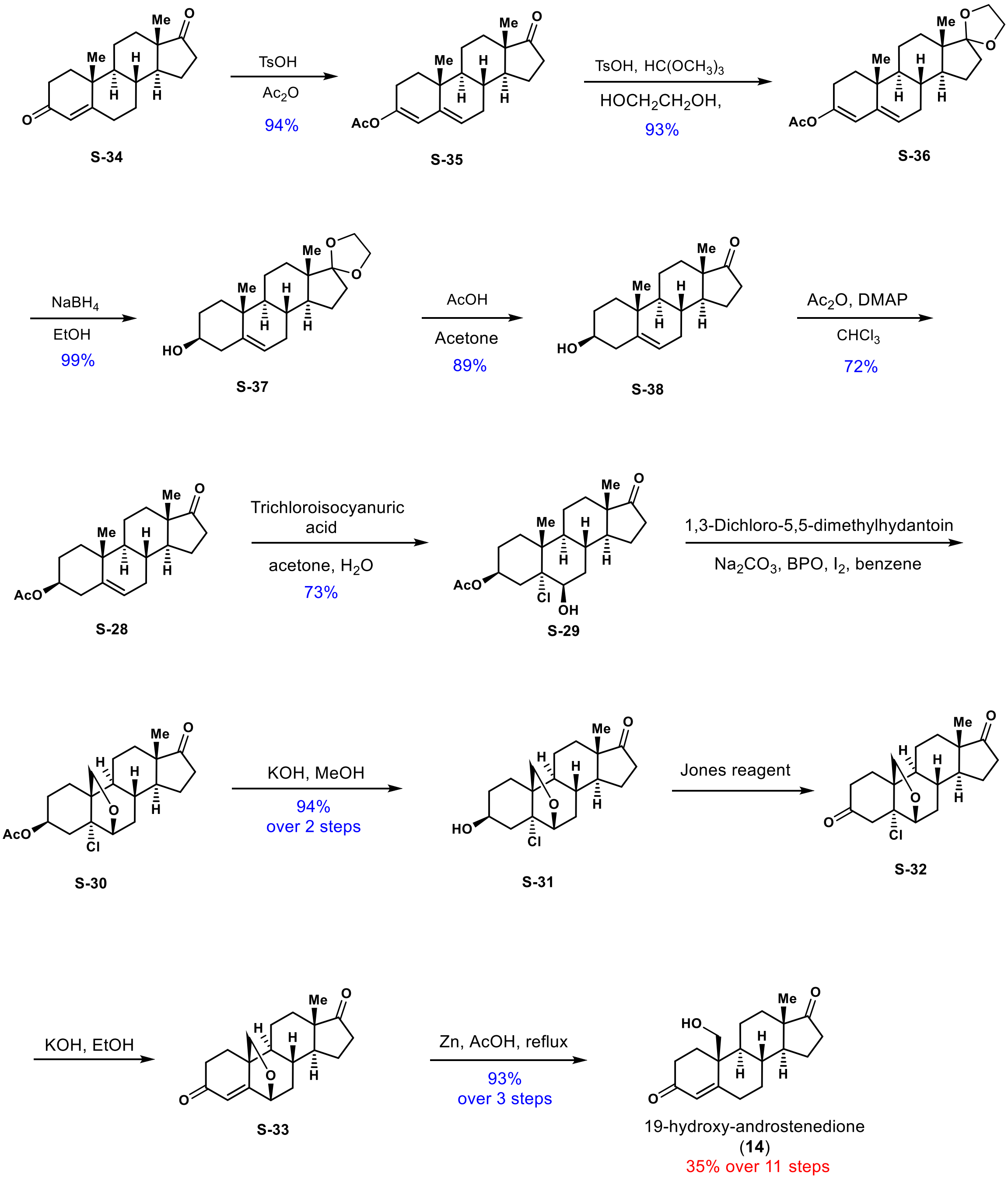

Supplementary Figure 11. Reported synthetic route B of **14**[5]

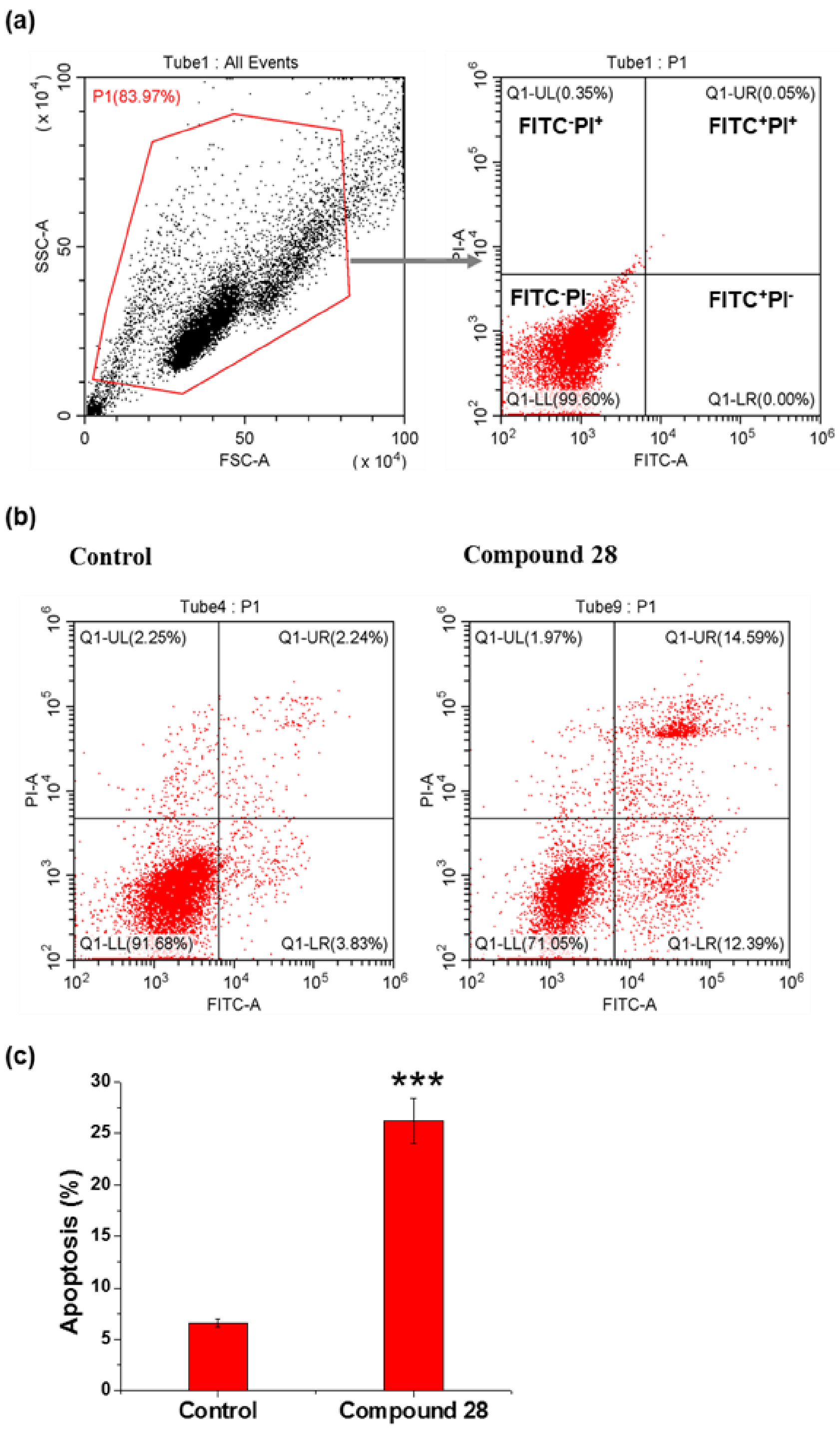

Supplementary Figure 12. Flow cytometry-based apoptosis assay of compound **28** on the HT-29 cells. (a) Gating strategy to sort apoptotic cells from the whole events. Each panel respectively stands for the distribution of normal cells (FITC$^-$PI$^-$), the early apoptotic cells (FITC$^+$PI$^-$), the late apoptotic cells (FITC$^+$PI$^+$), and the necrotic cells (FITC$^-$PI$^+$). (b) Using above Gating strategy, flow cytometry-based apoptosis assay of compound **28** for HT-29 cells. Triplicate experiments were conducted for each group, figure shown as representative results. (c) The statistical quantification of cell apoptosis(FITC$^+$PI$^-$ and FITC$^+$PI$^+$), triplicate experiments were conducted for each group (***$p < 0.001$ versus control group, n=3 biologically independent experiments). Source data are provided as a Source Data file.

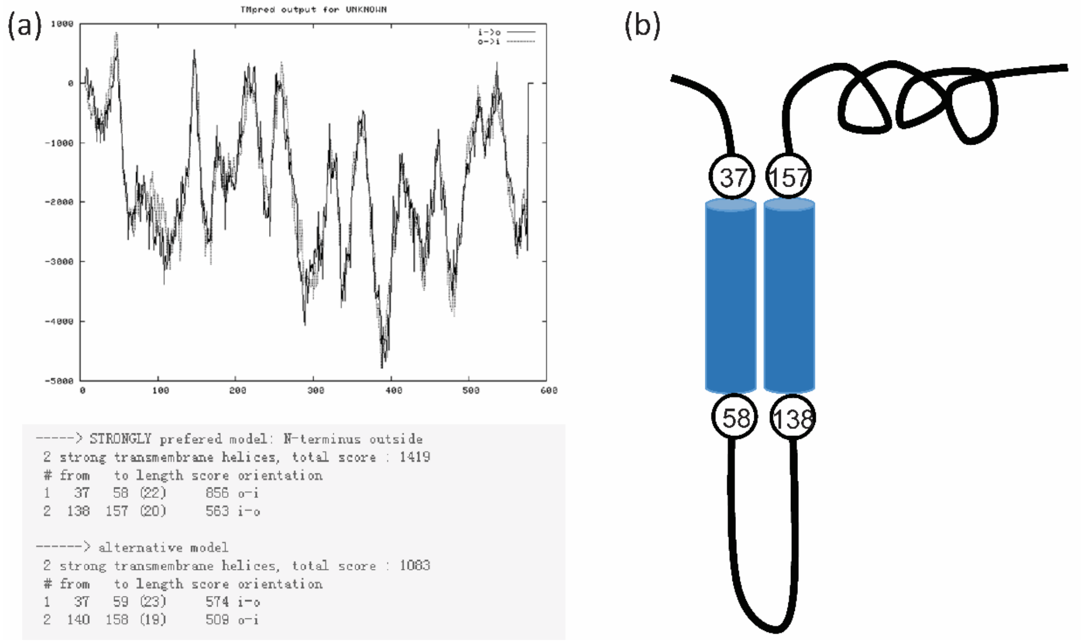

Supplementary Figure 13. Analysis of the transmembrane segments in tcP450-1. (a) Analysis results by using TMbase (https://embnet.vital-it.ch/software/TMPRED_form.html);[6] (b) Cartoon for illustration of the transmembrane segments in tcP450-1.

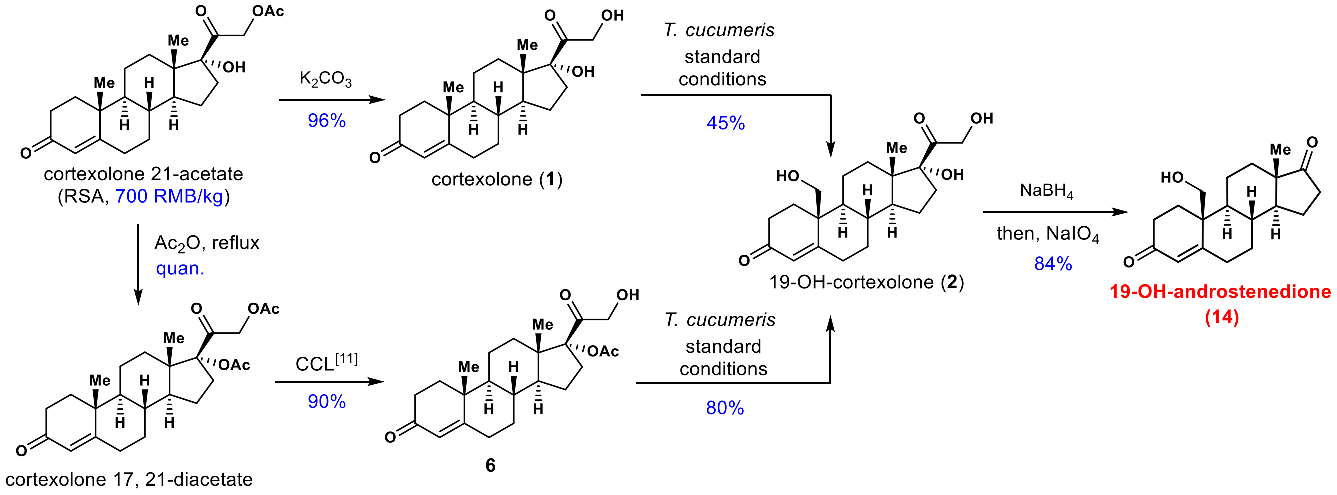

Supplementary Figure 14. Our strategy for 19-OH-androstenedione (**14**) synthesis

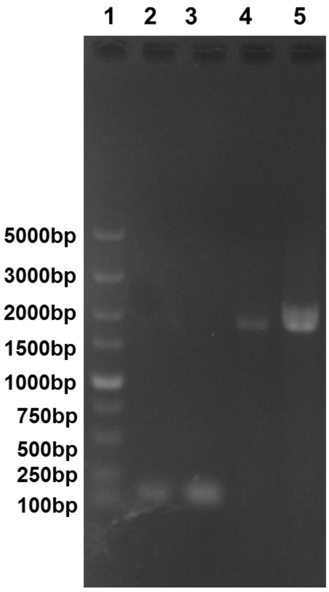

Supplementary Figure 15. Verification of transformants genotype. Lane 1: maker; Lane 2：mWHU2488; Lane 3: mWHU2488; Lane 4: pWHU2490 (1755bp); Lane 5: mWHU2487. Source data are provided as a Source Data file.

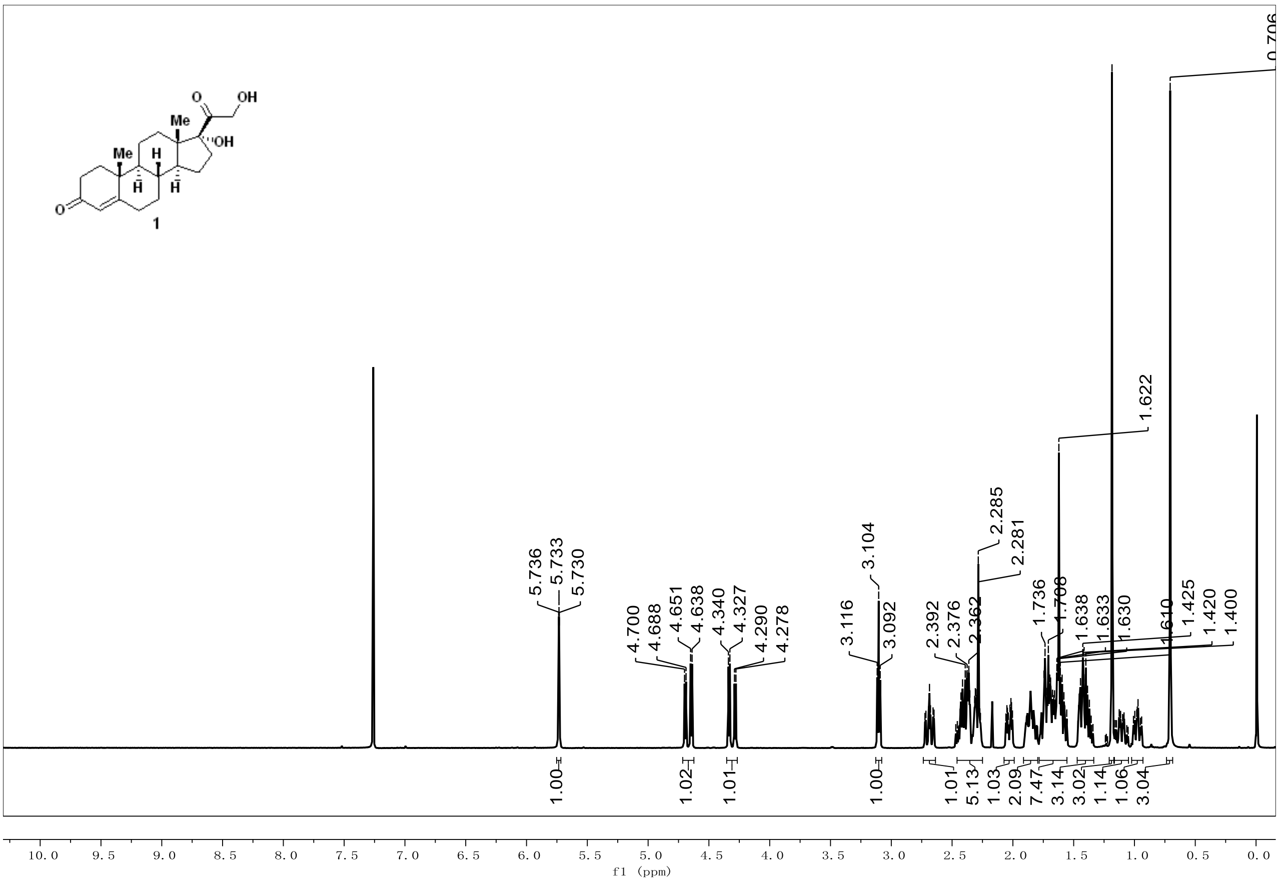

Supplementary Figure 16. ^{1}H NMR of **1**

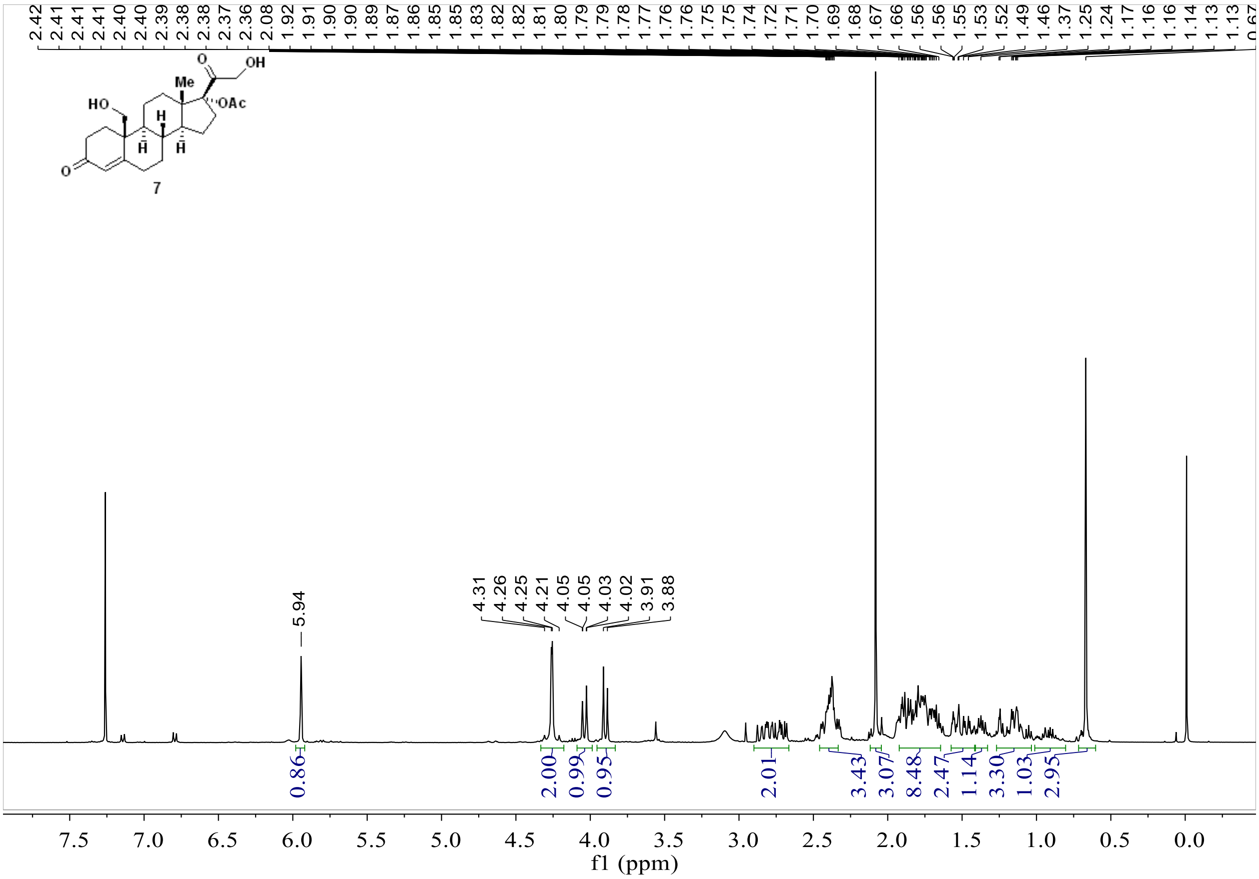

Supplementary Figure 17. ^{1}H NMR of **7**

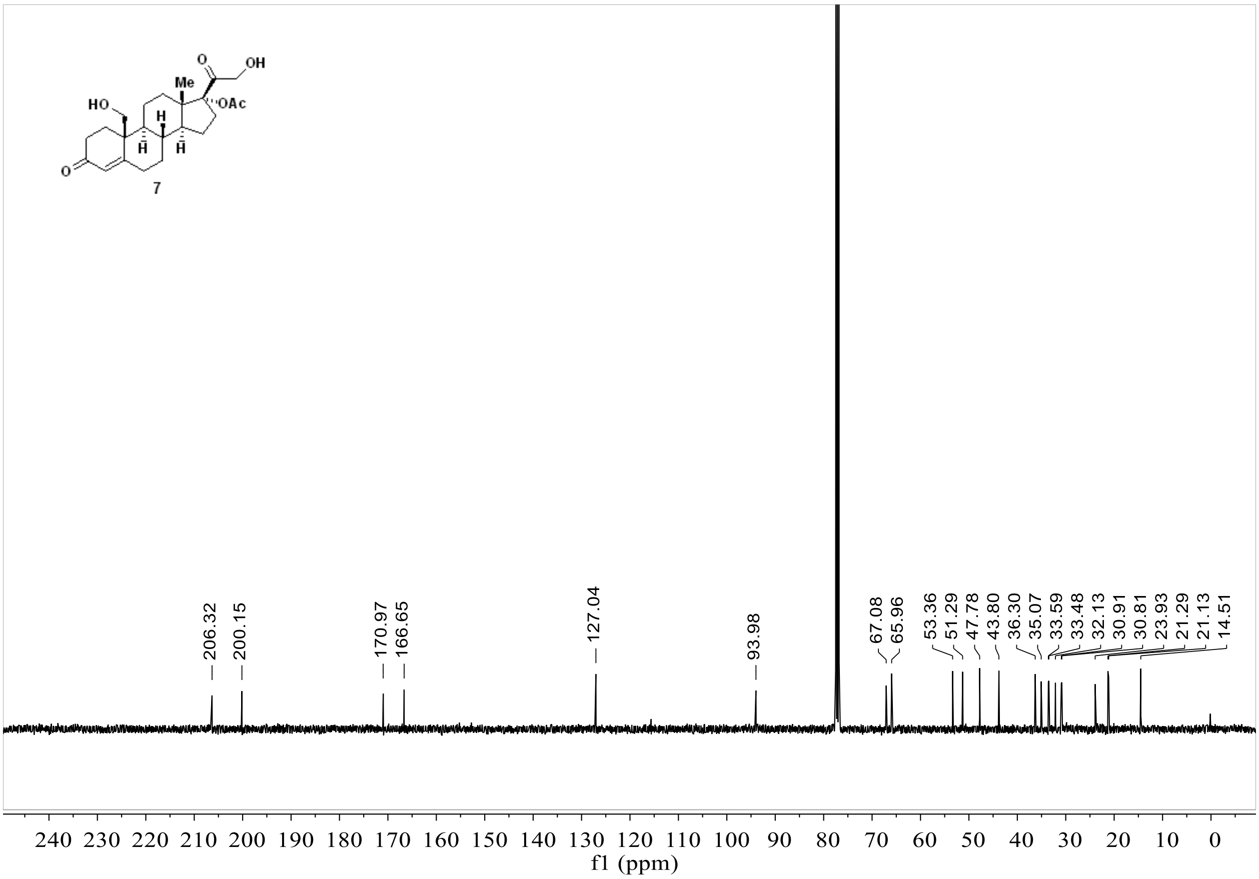

Supplementary Figure 18. ^{13}C NMR of **7**

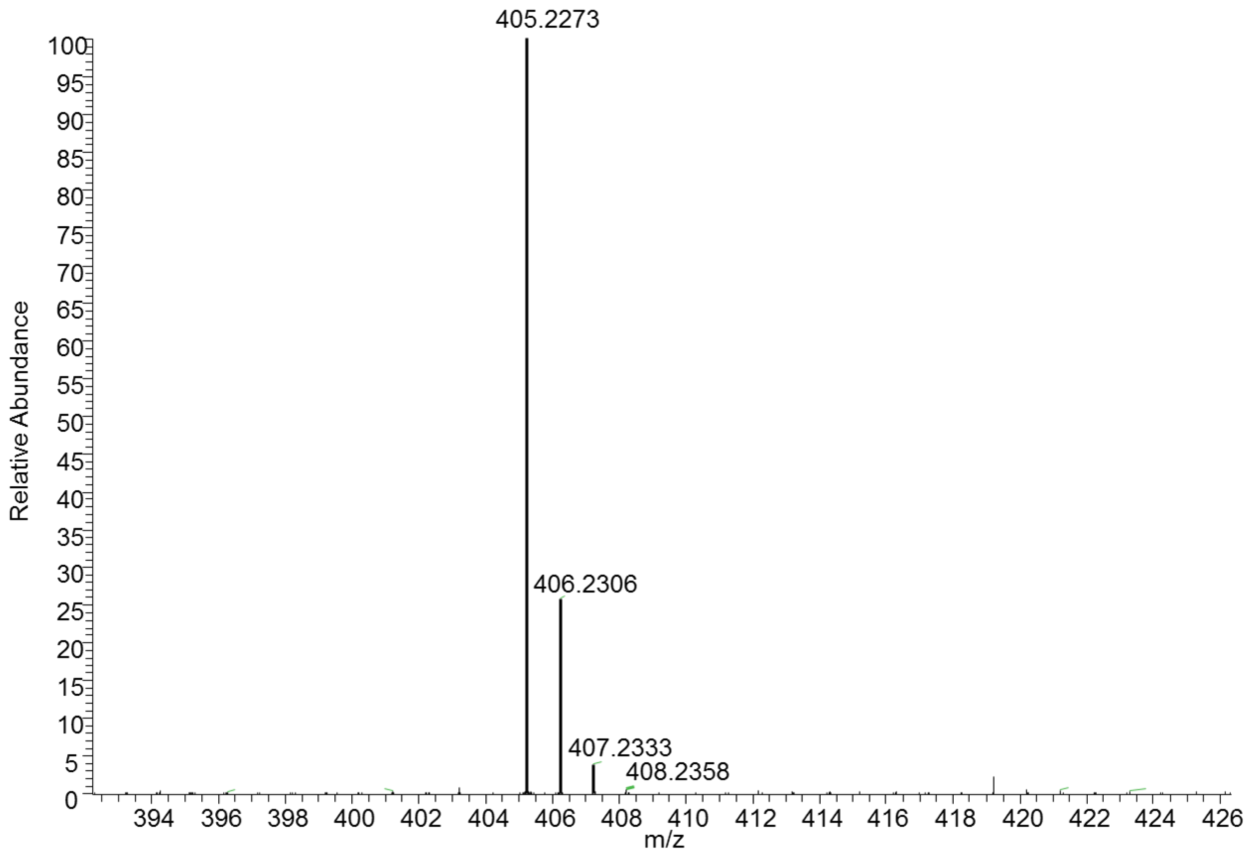

Supplementary Figure 19. HRMS of **7**

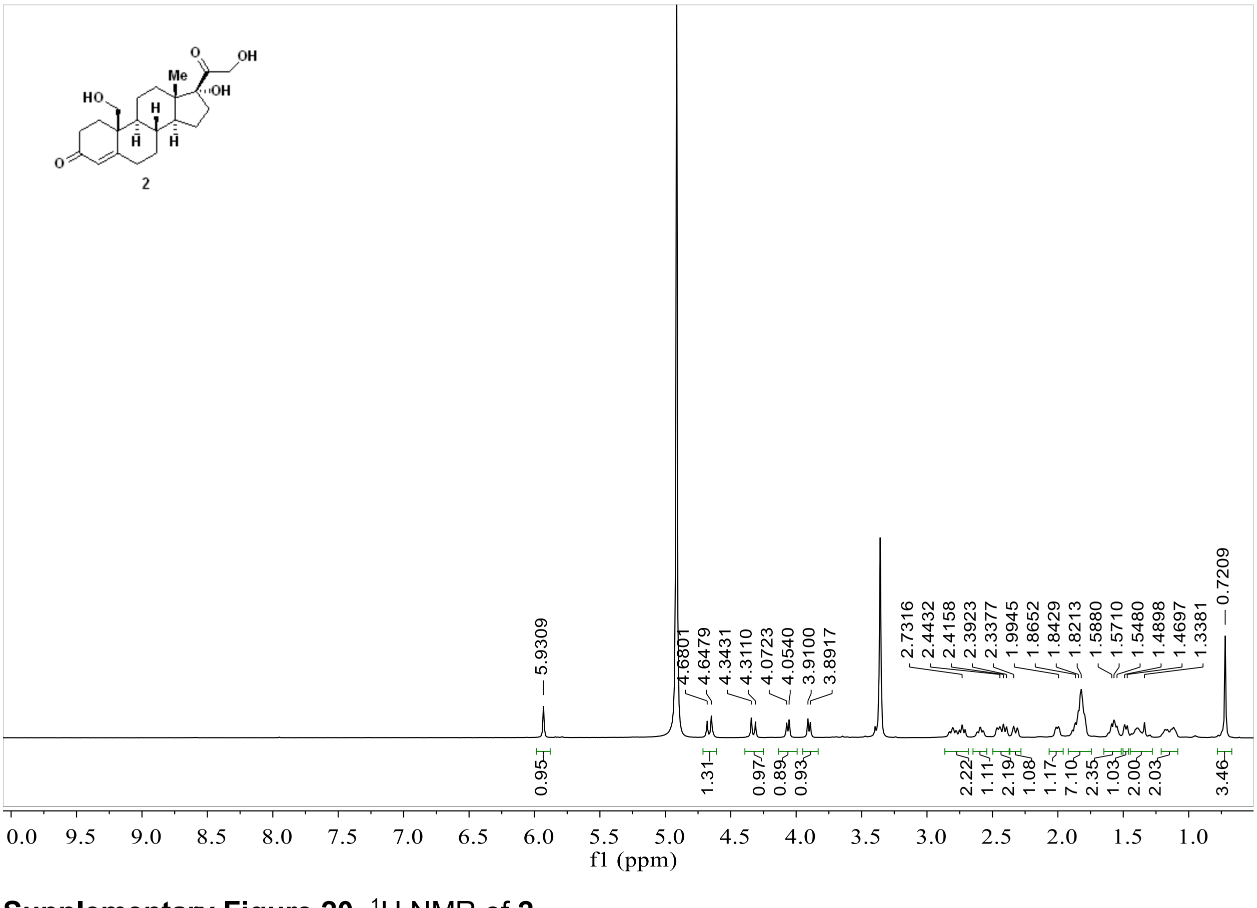

Supplementary Figure 20. ^{1}H NMR of **2**

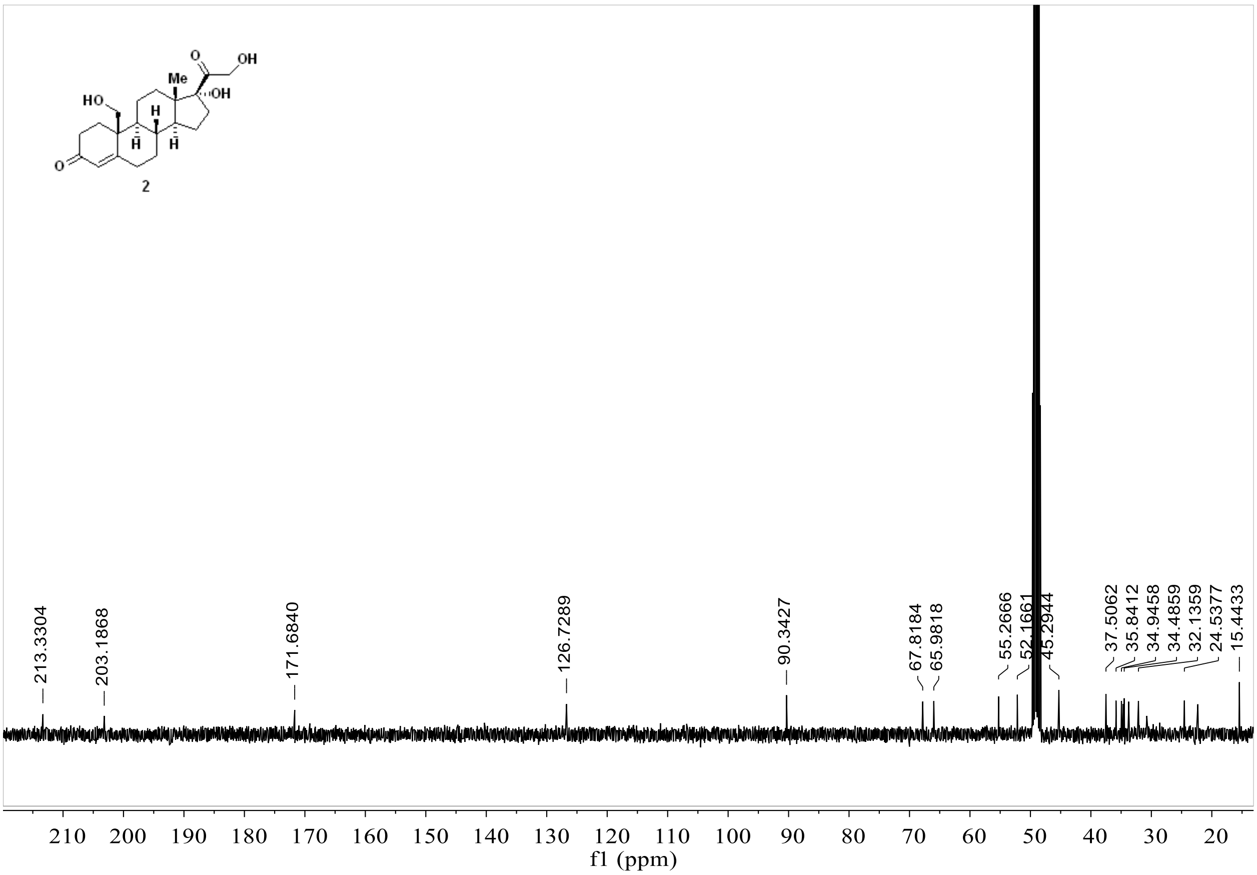

Supplementary Figure 21. ^{13}C NMR of **2**

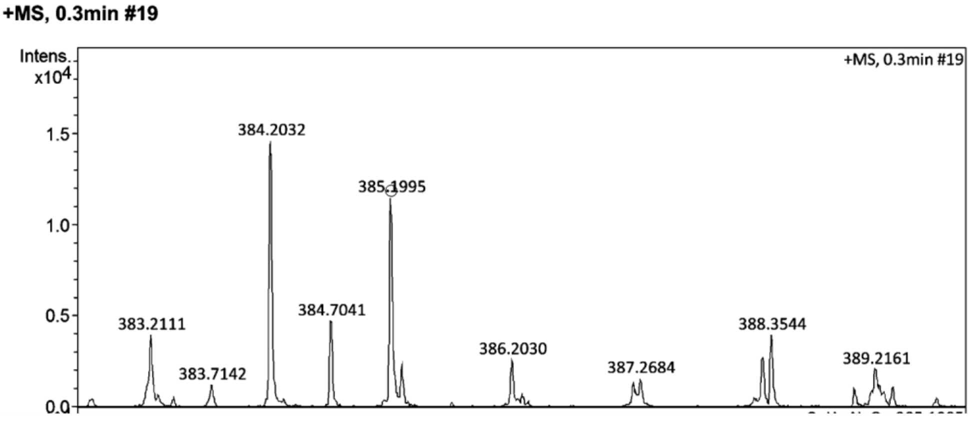

Supplementary Figure 22. HRMS of **2**

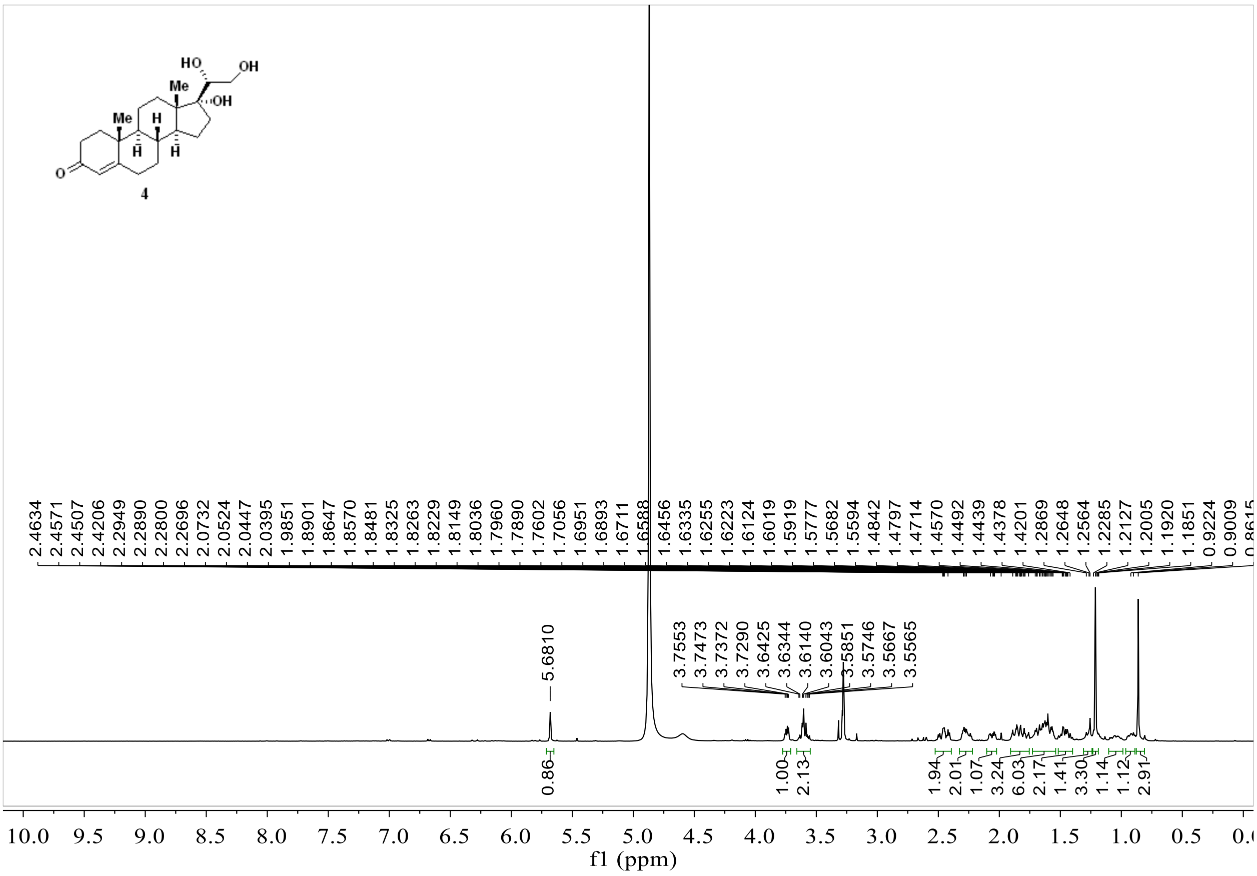

Supplementary Figure 23. ^{1}H NMR of **4**

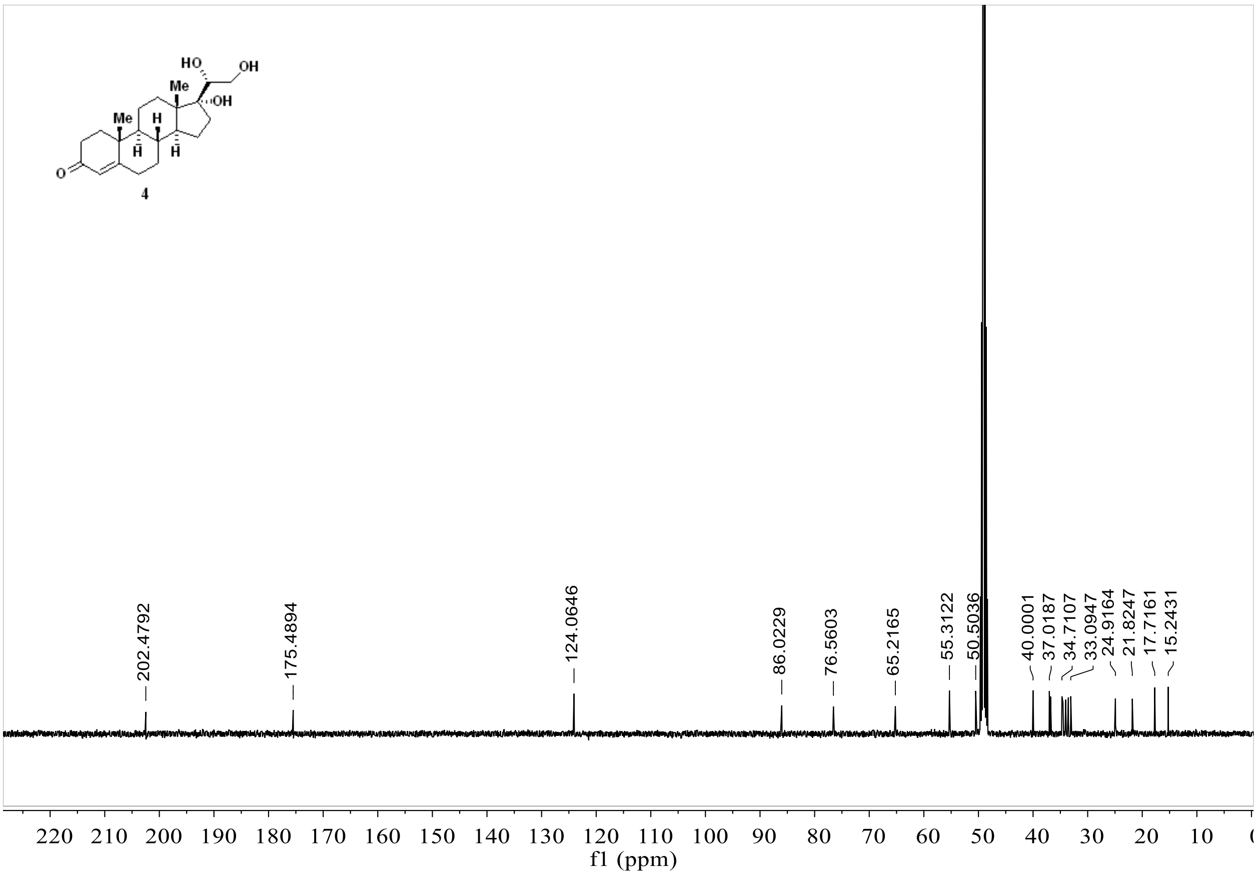

Supplementary Figure 24. ^{13}C NMR of **4**

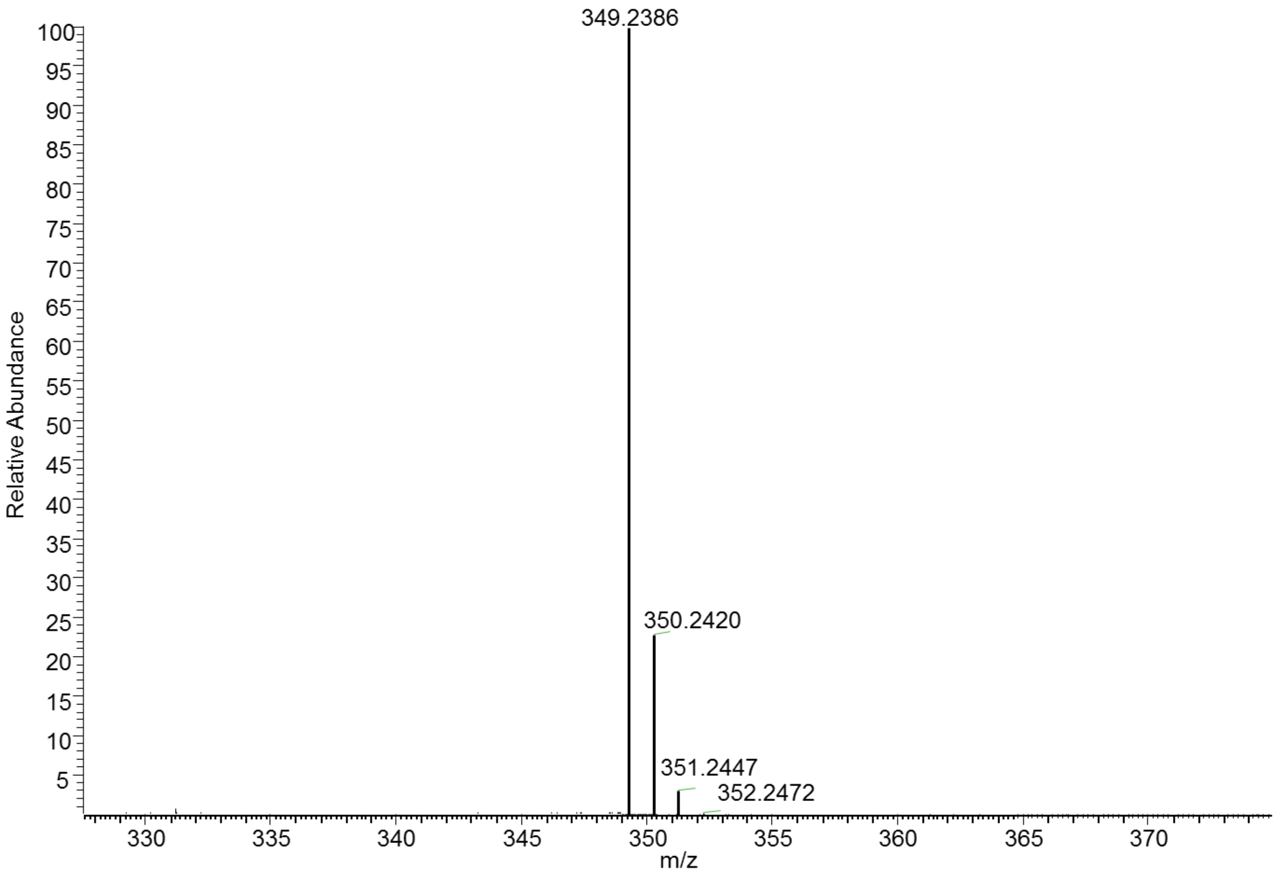

Supplementary Figure 25. HRMS of **4**

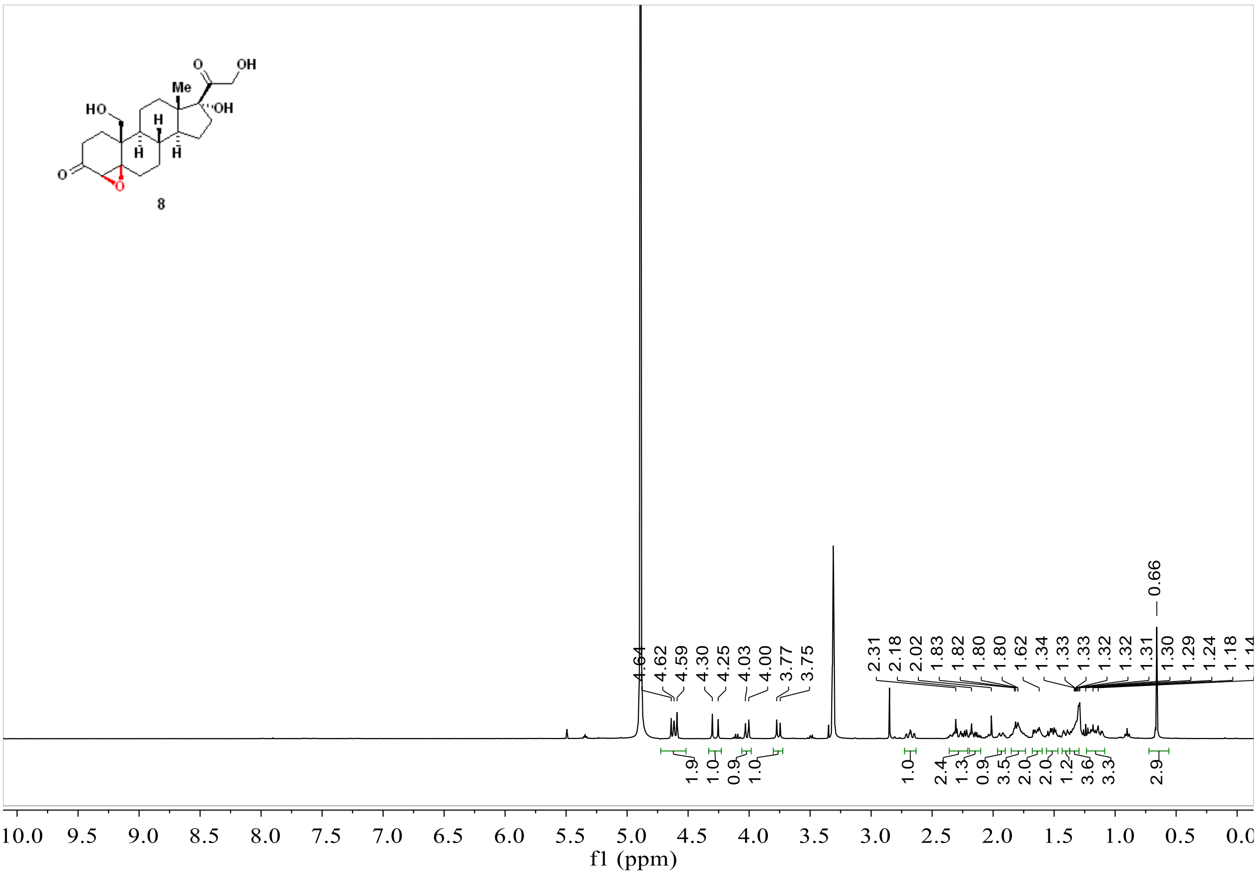

Supplementary Figure 26. ^{1}H NMR of **8**

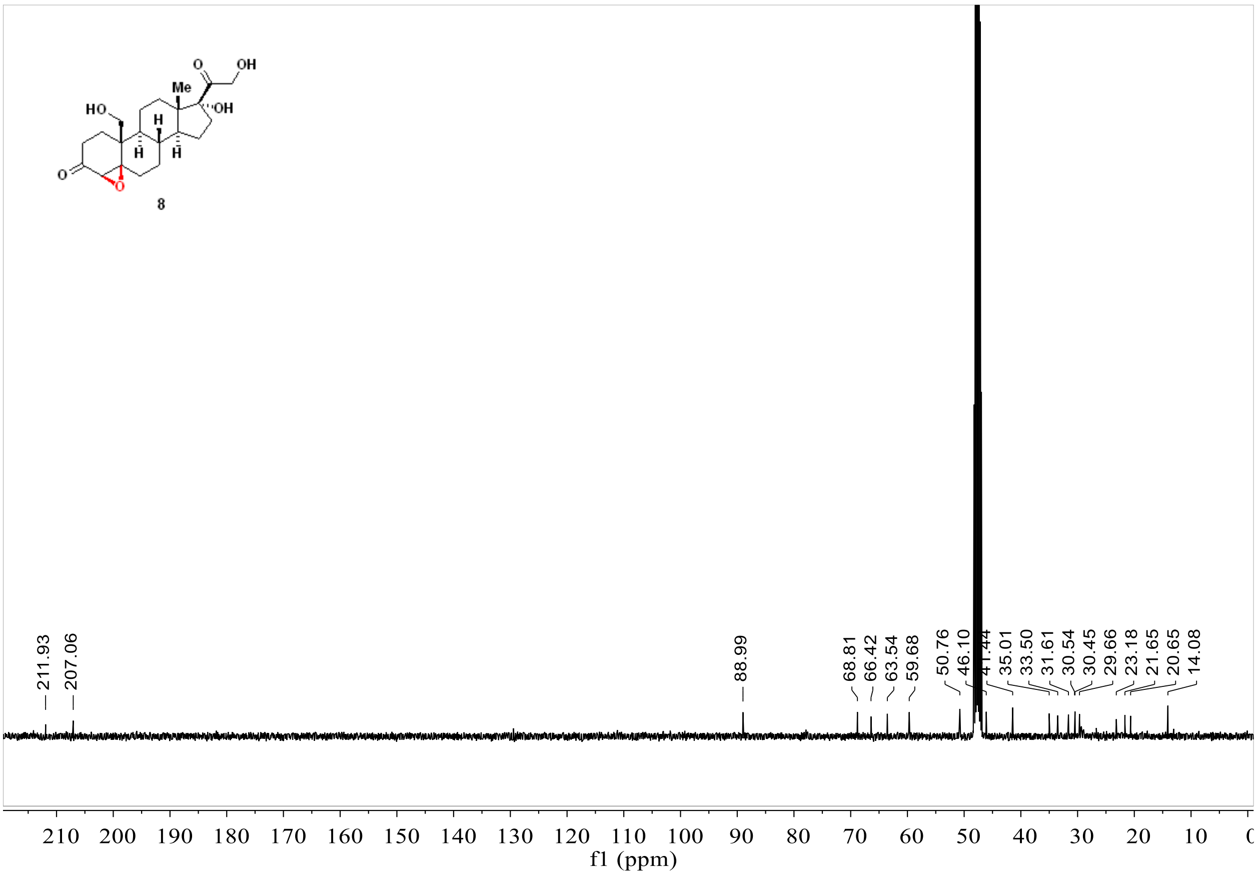

Supplementary Figure 27. ^{13}C NMR of **8**

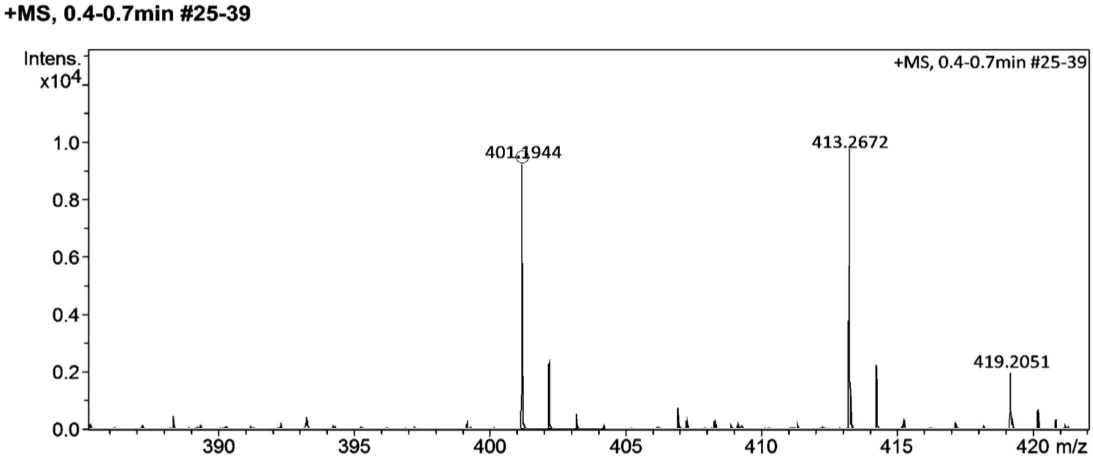

Supplementary Figure 28. HRMS of **8**

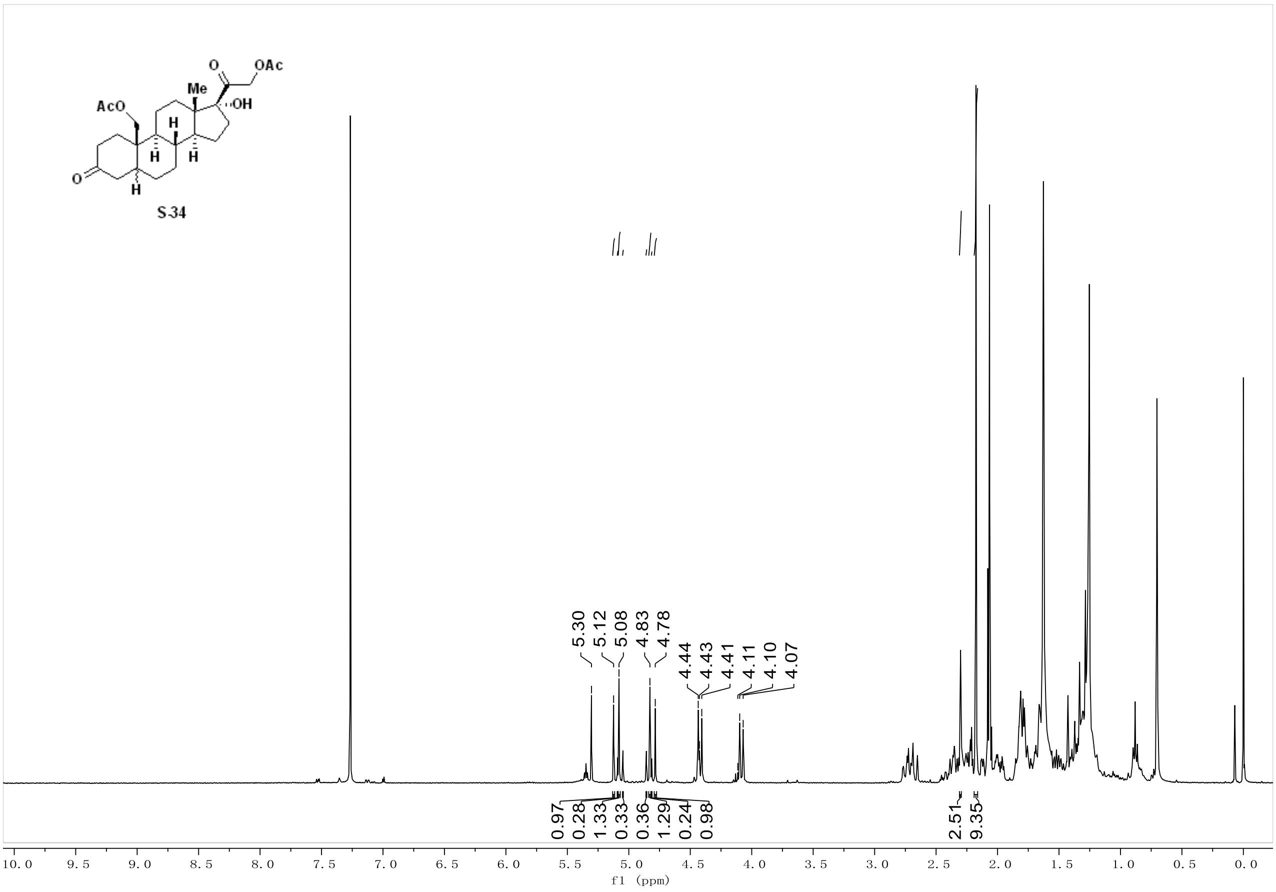

Supplementary Figure 29. ^{1}H NMR of **S-34**

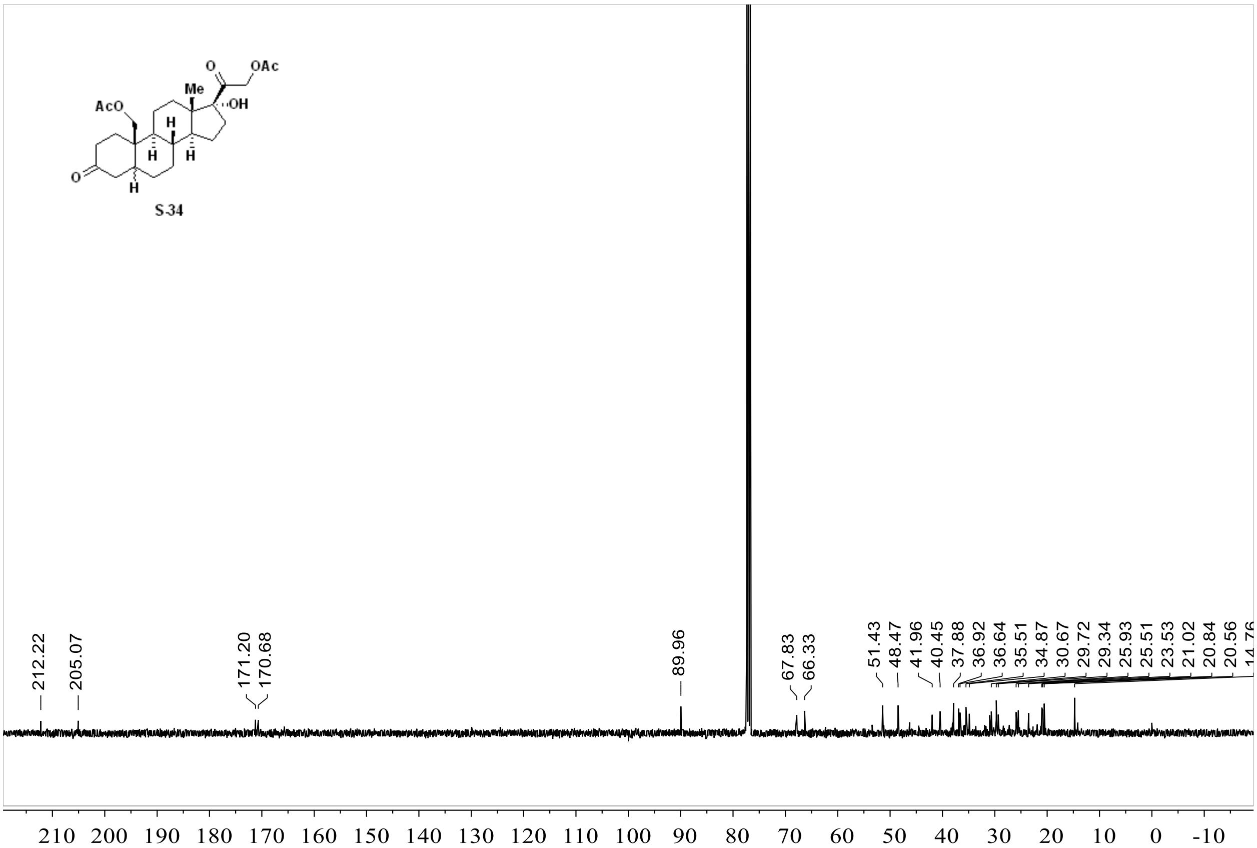

Supplementary Figure 30. ^{13}C NMR of **S-34**

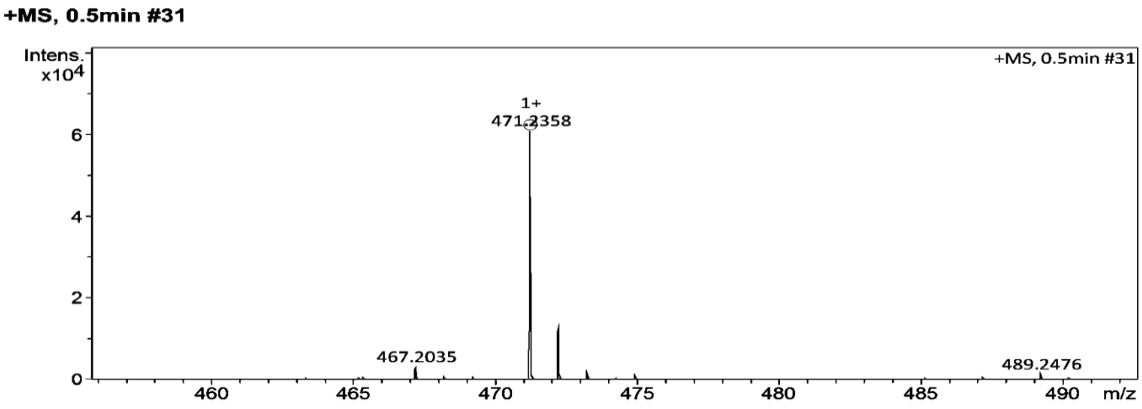

Supplementary Figure 31. HRMS of **S-34**

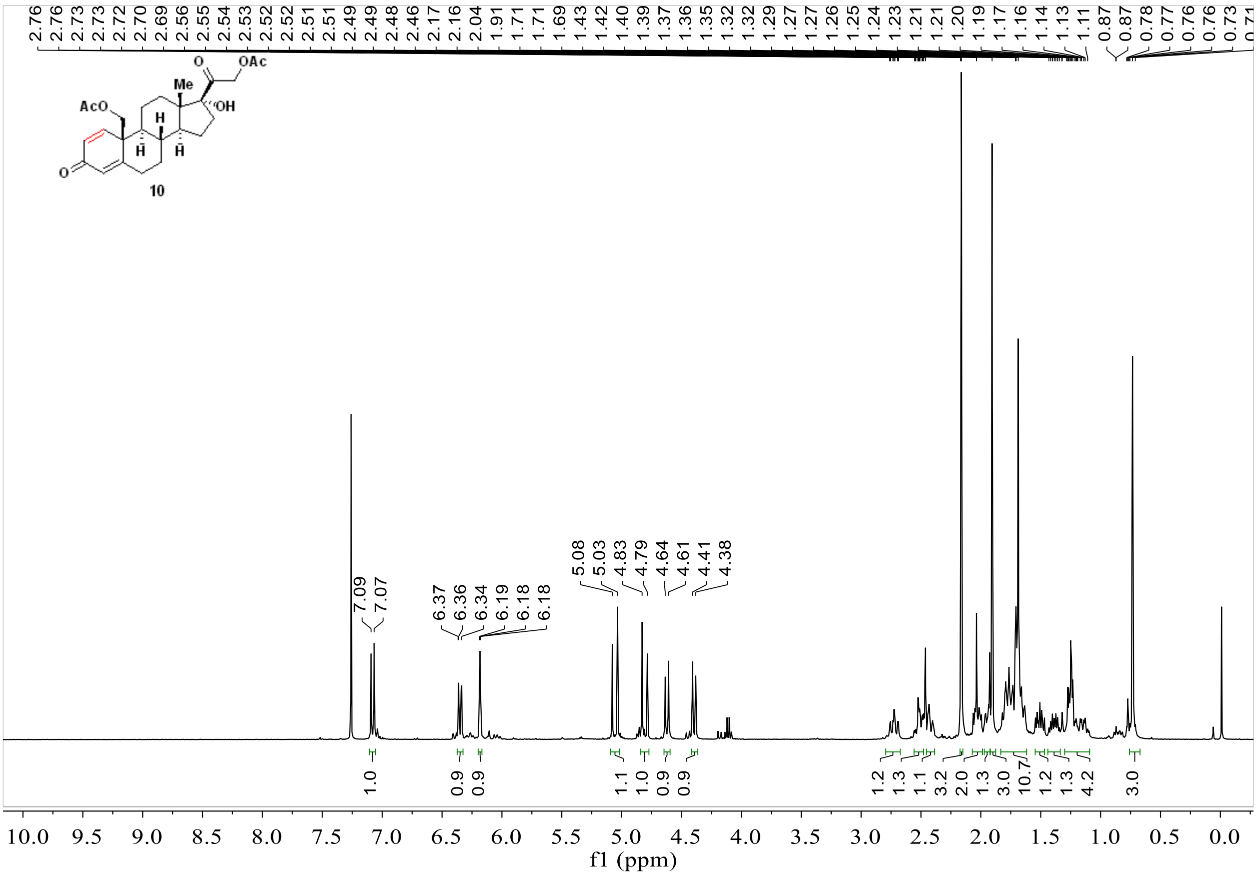

Supplementary Figure 32. ^{1}H NMR of **10**

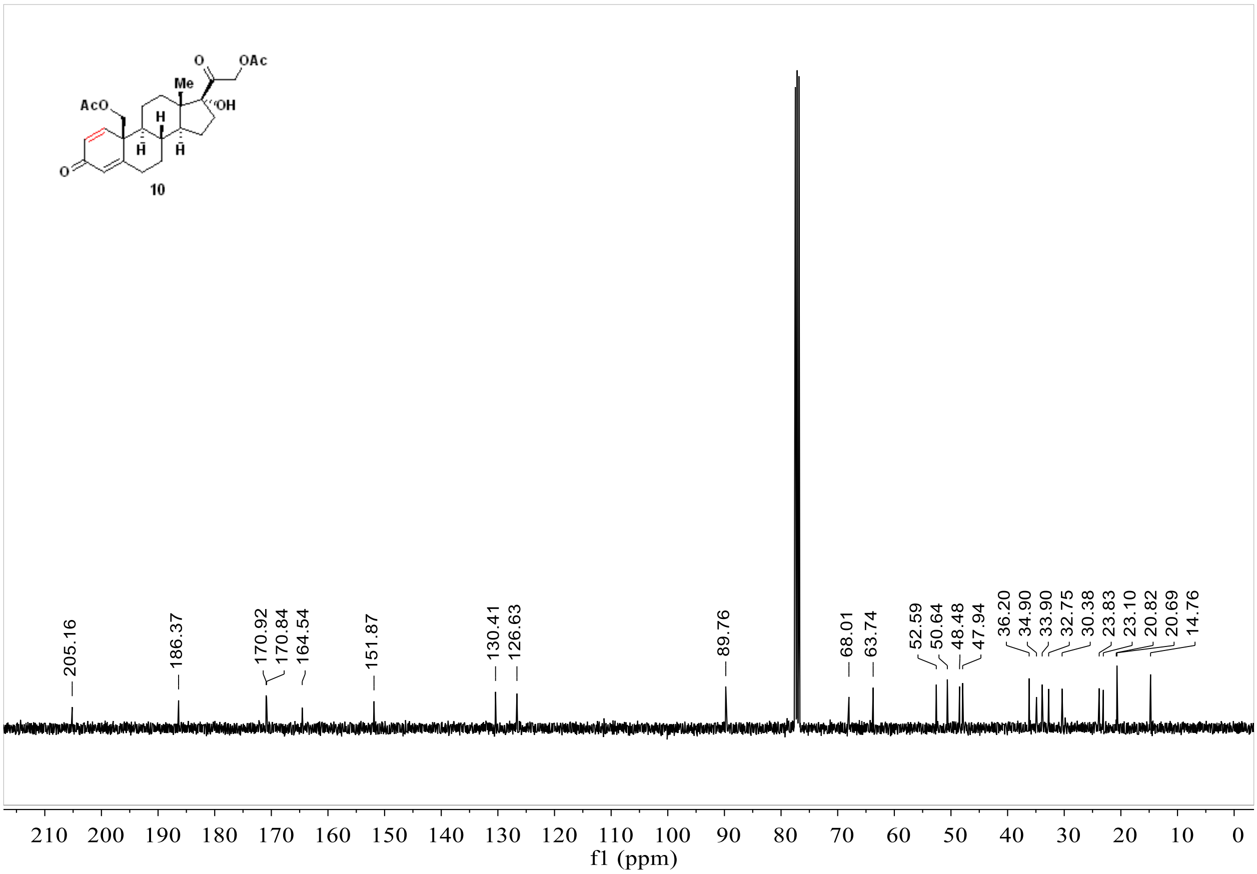

Supplementary Figure 33. ^{13}C NMR of **10**

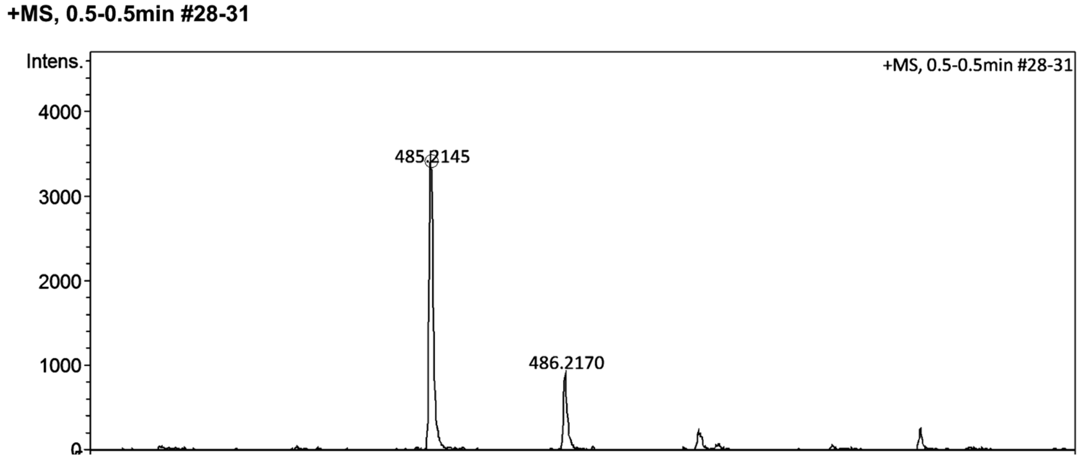

Supplementary Figure 34. HRMS of **10**

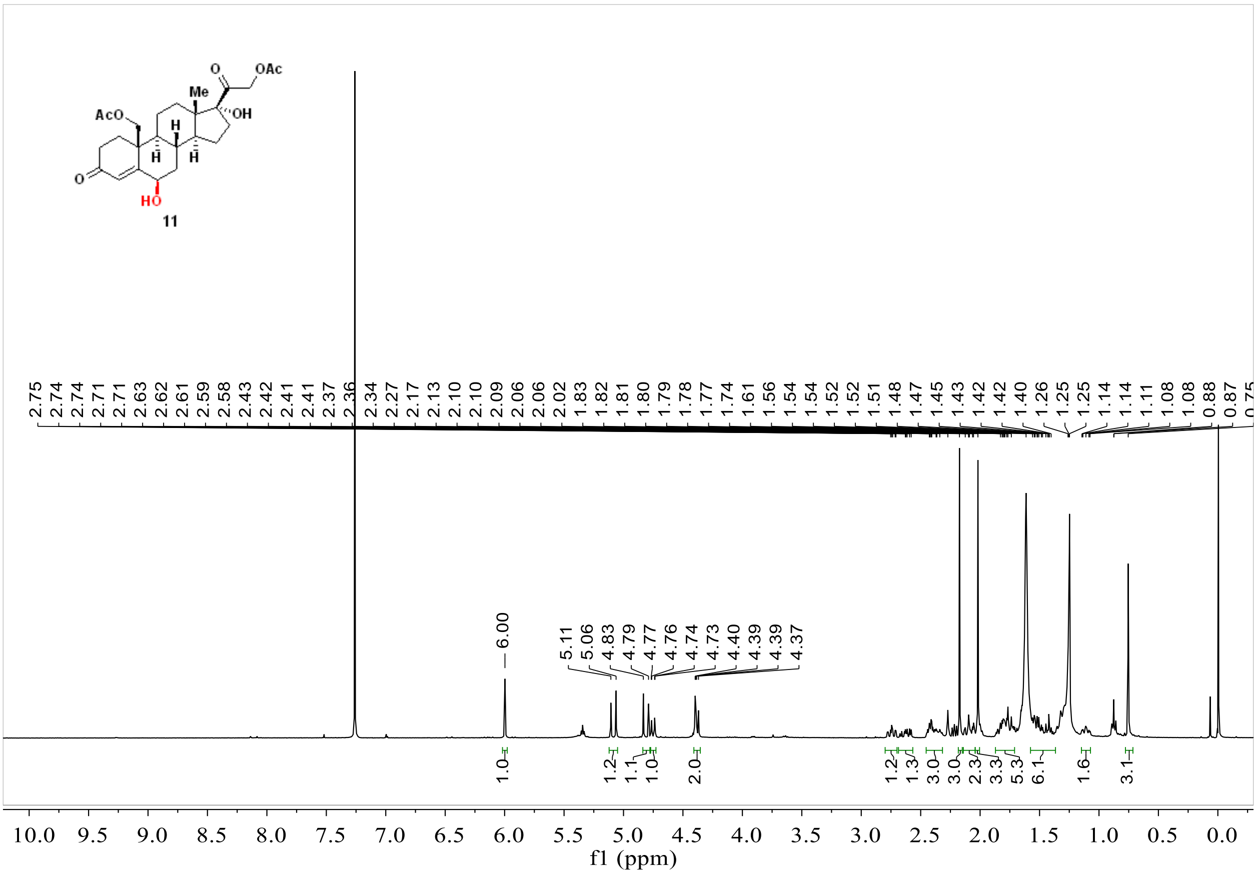

Supplementary Figure 35. ^{1}H NMR of **11**

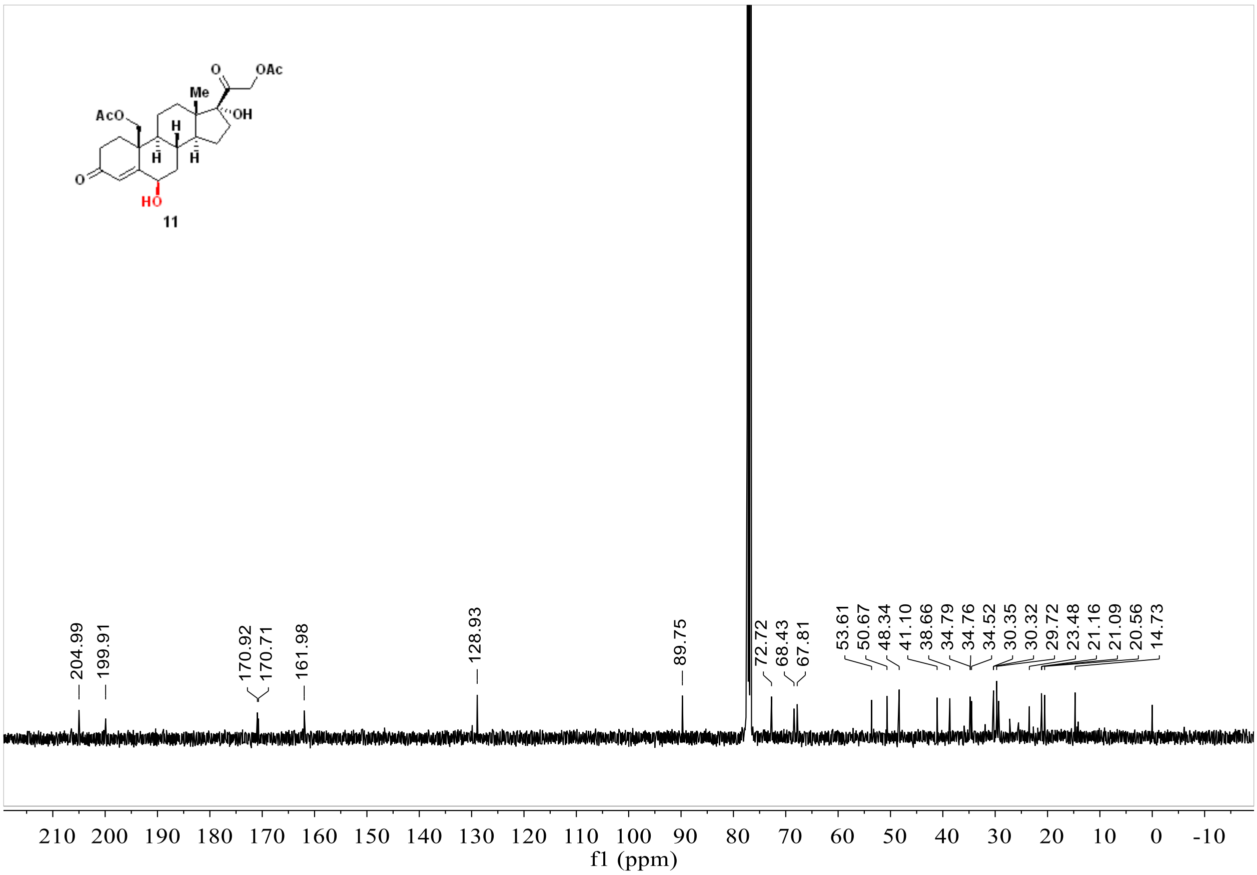

Supplementary Figure 36. ^{13}C NMR of **11**

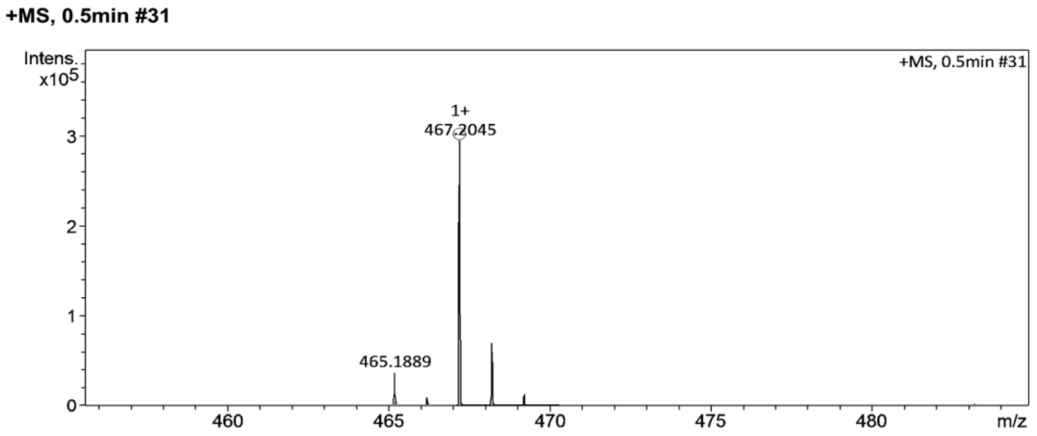

Supplementary Figure 37. HRMS of **11**

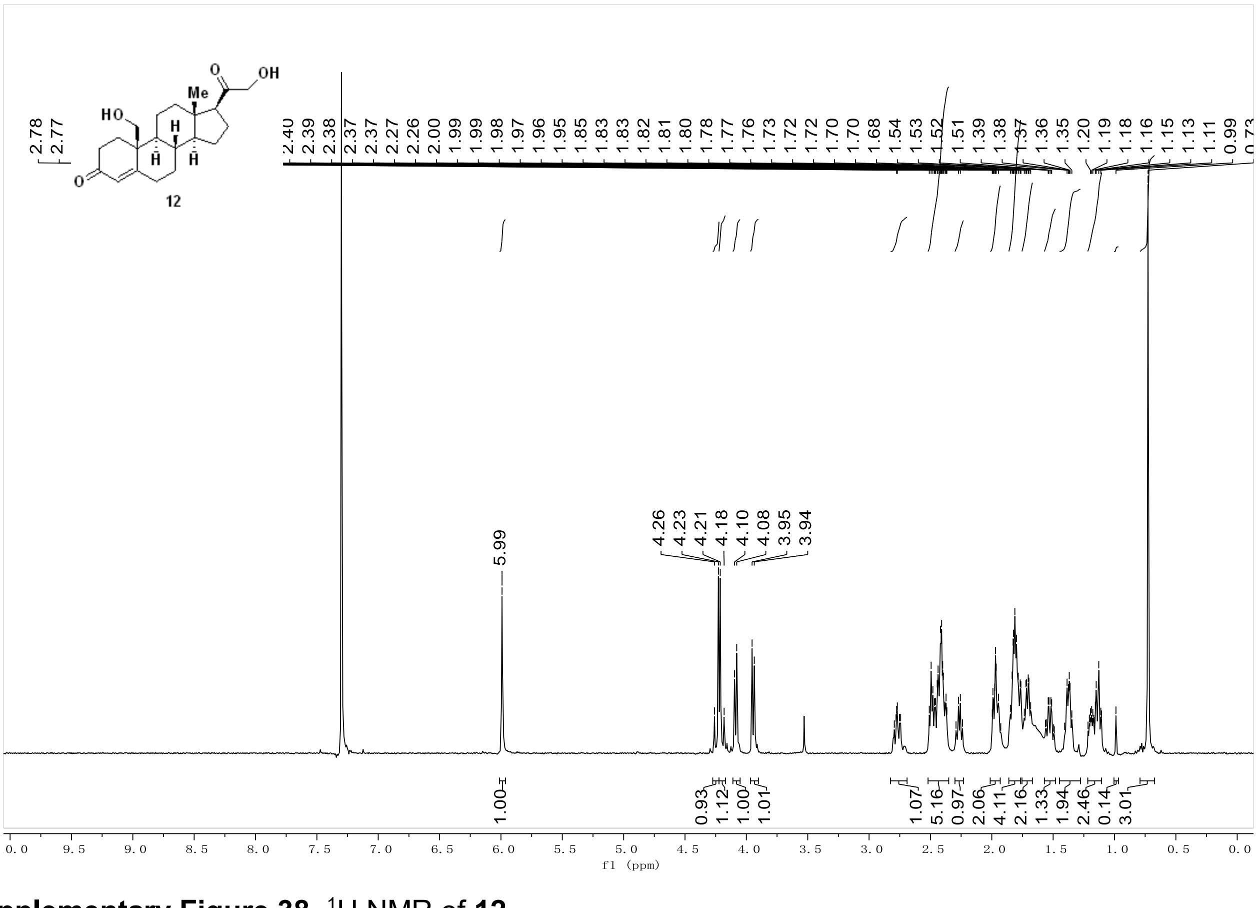

Supplementary Figure 38. ^{1}H NMR of **12**

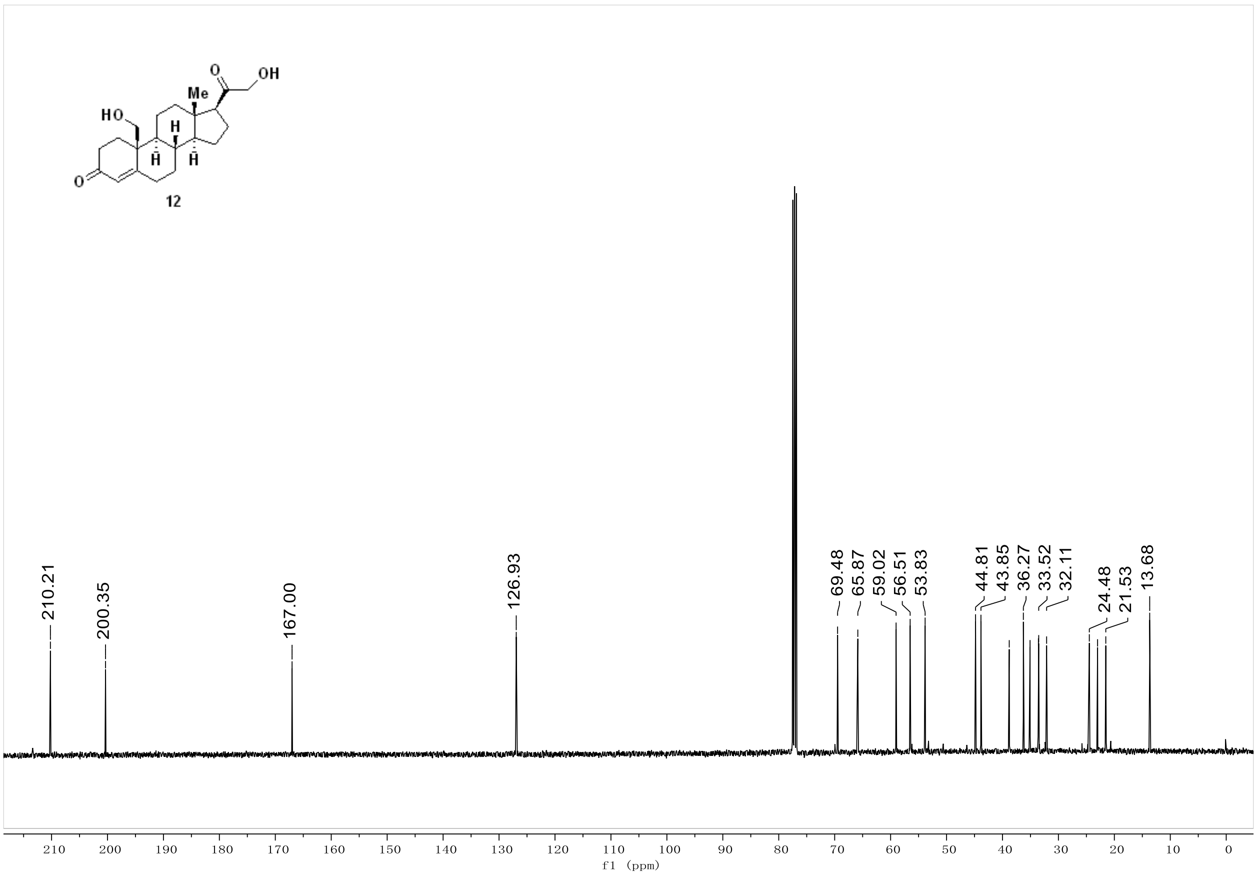

Supplementary Figure 39. ^{13}C NMR of **12**

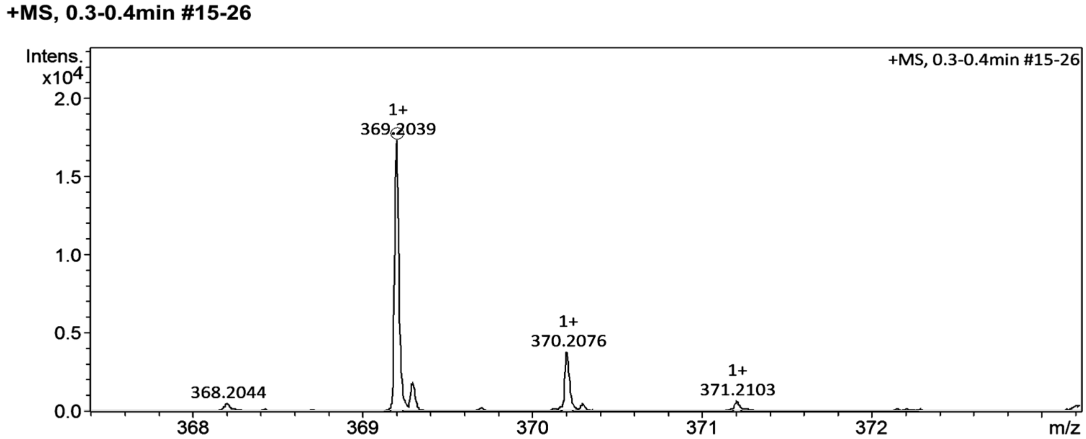

Supplementary Figure 40. HRMS of **12**

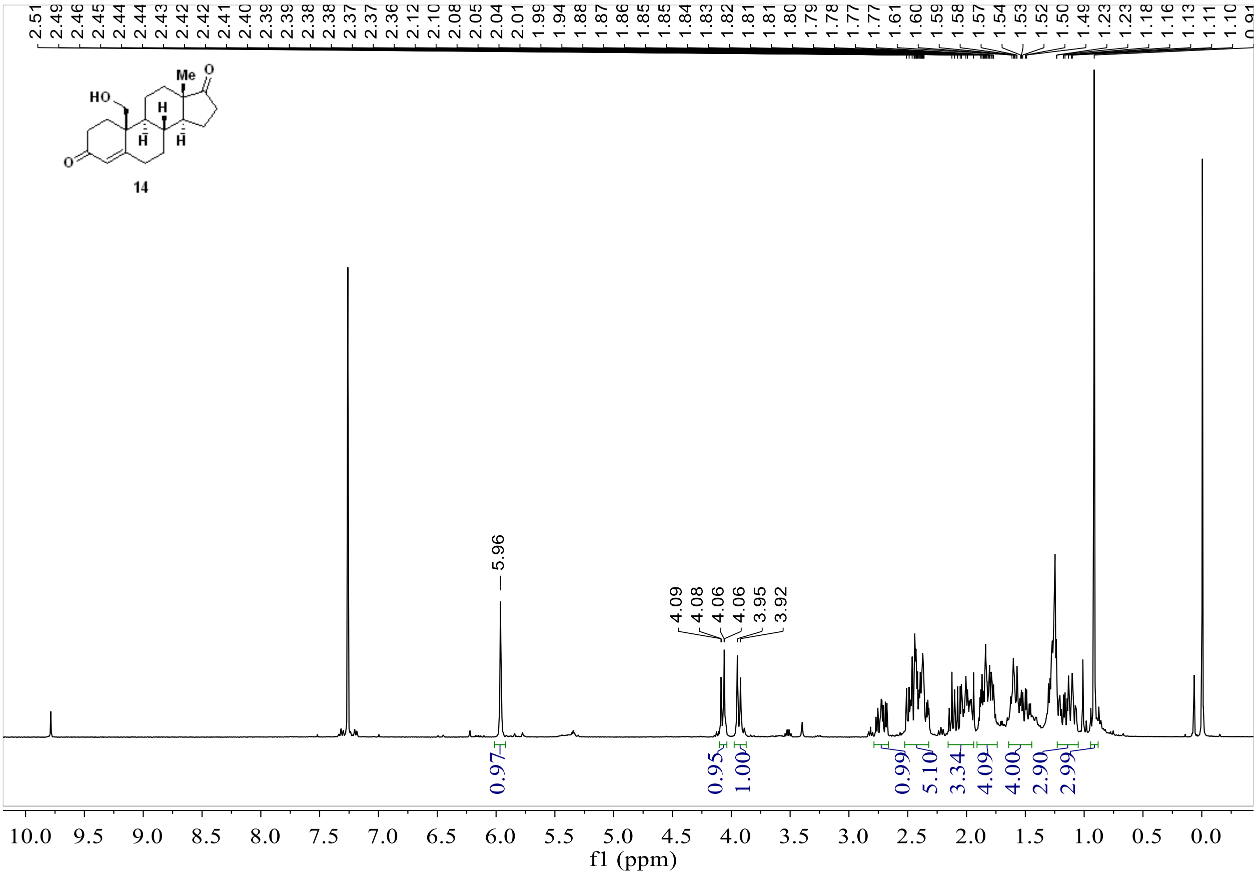

Supplementary Figure 41. ^{1}H NMR of **14**

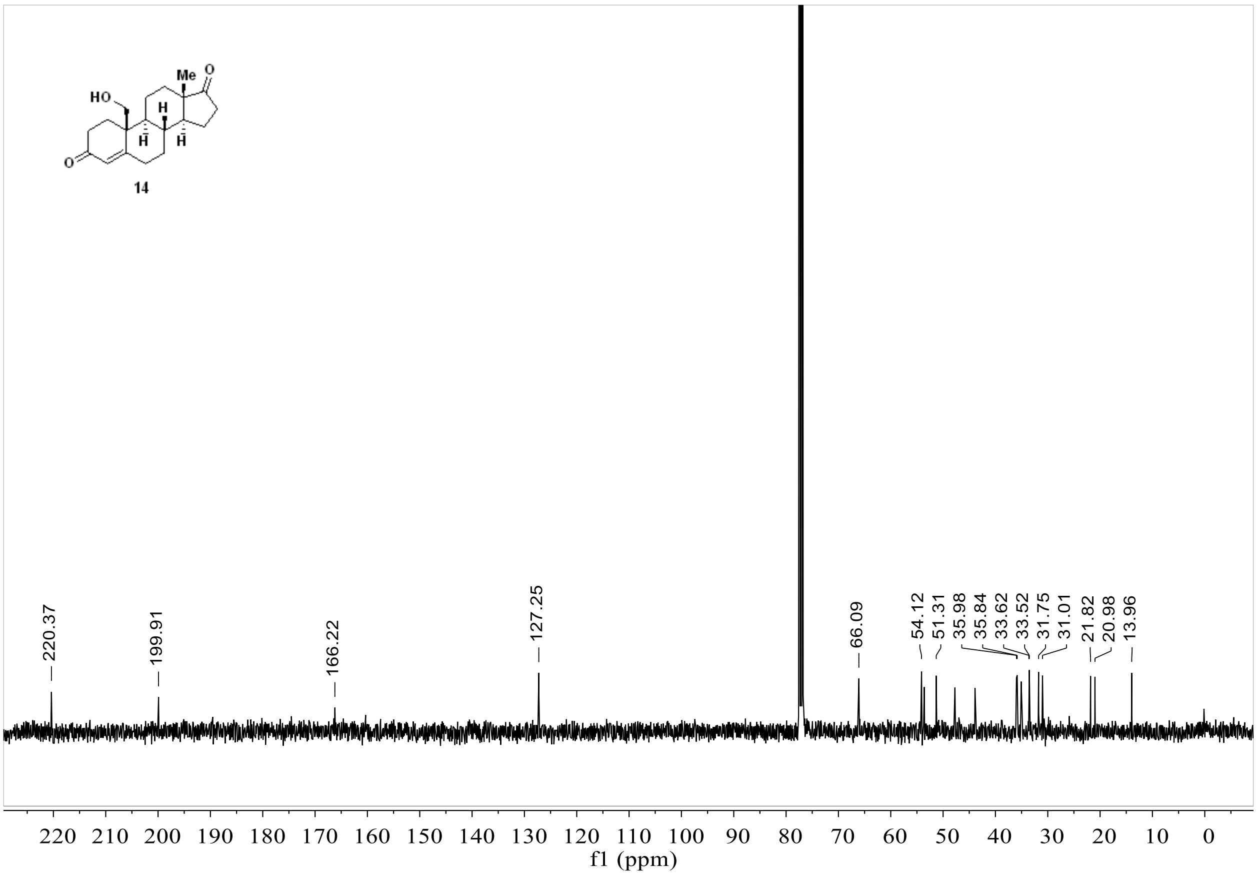

Supplementary Figure 42. ^{13}C NMR of **14**

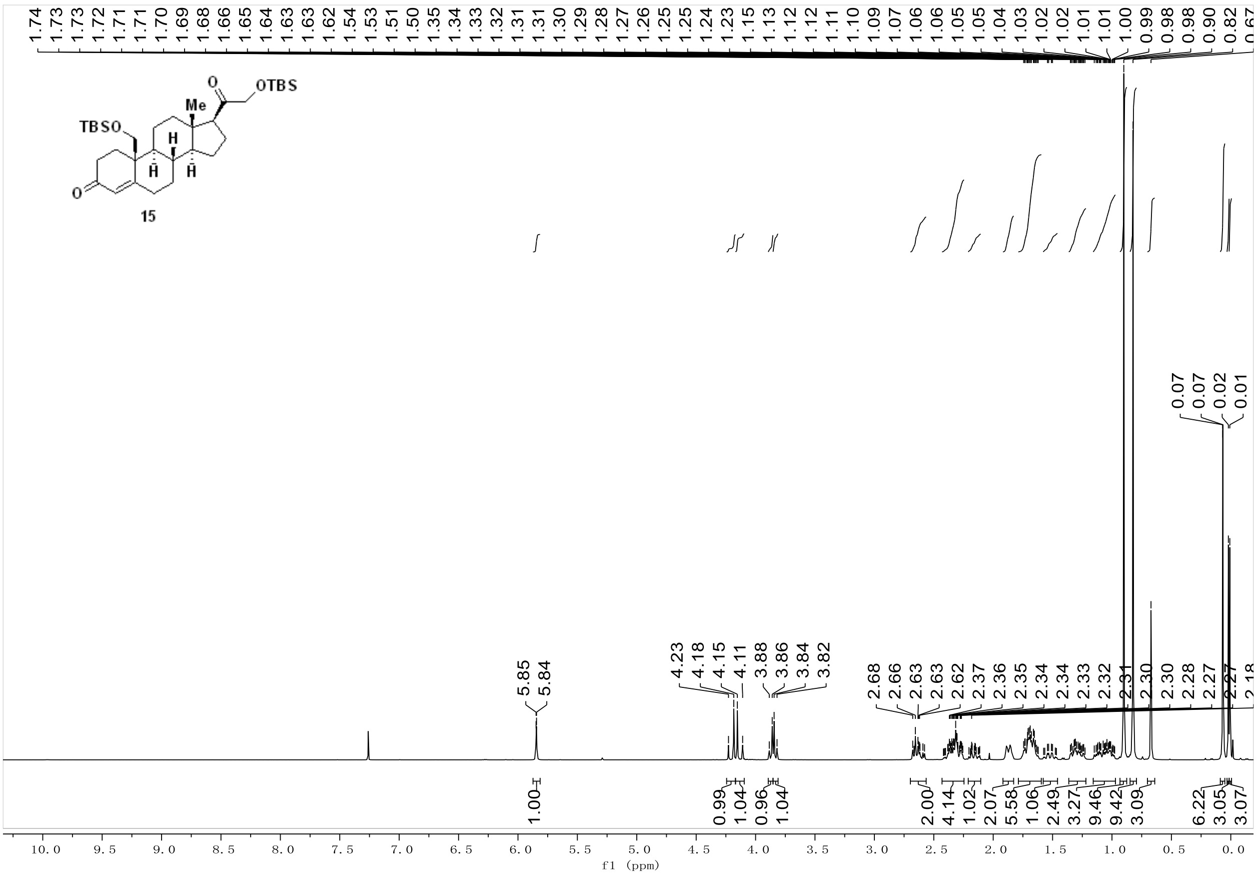

Supplementary Figure 43. ^{1}H NMR of **15**

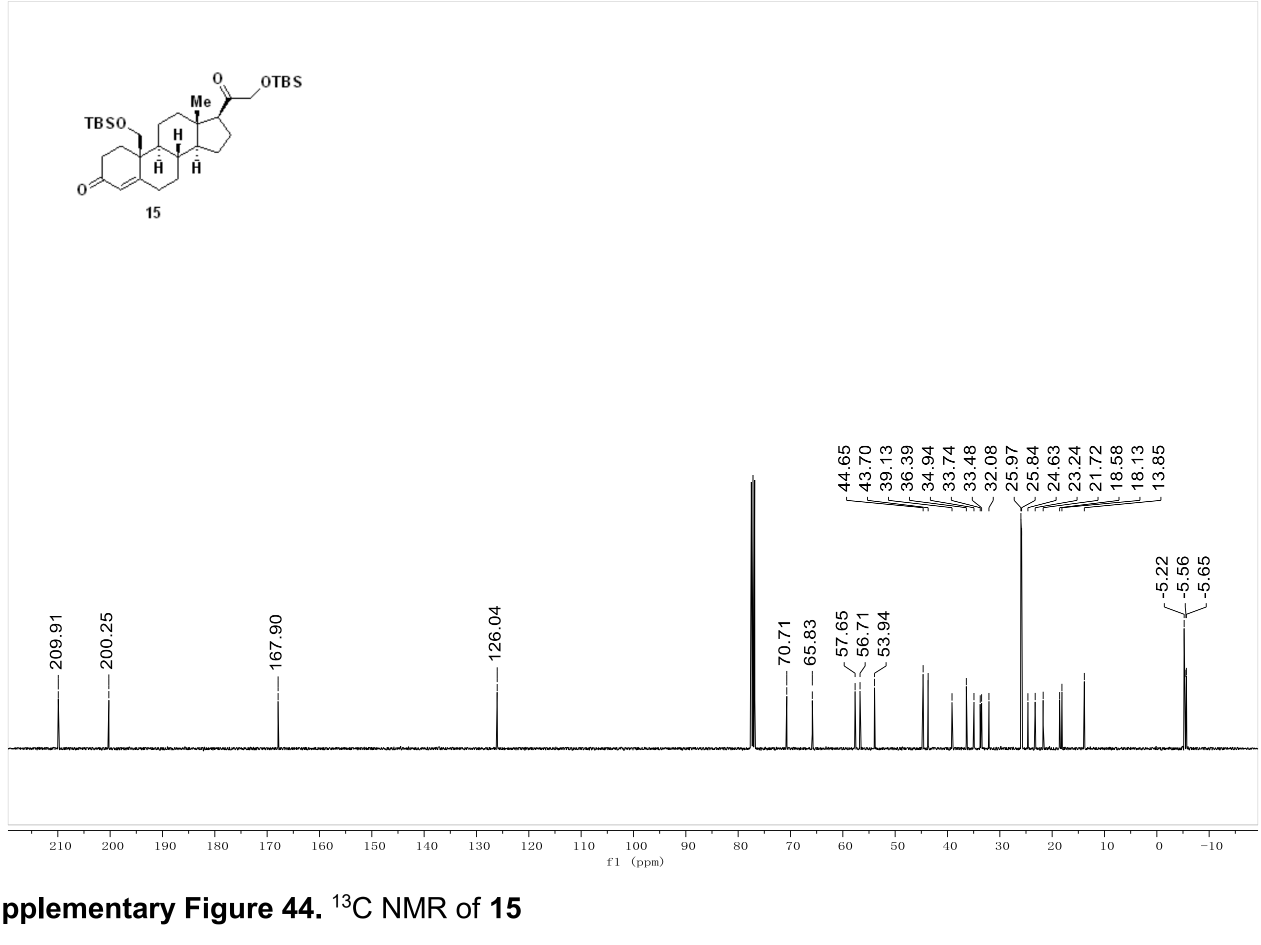

Supplementary Figure 44. ^{13}C NMR of **15**

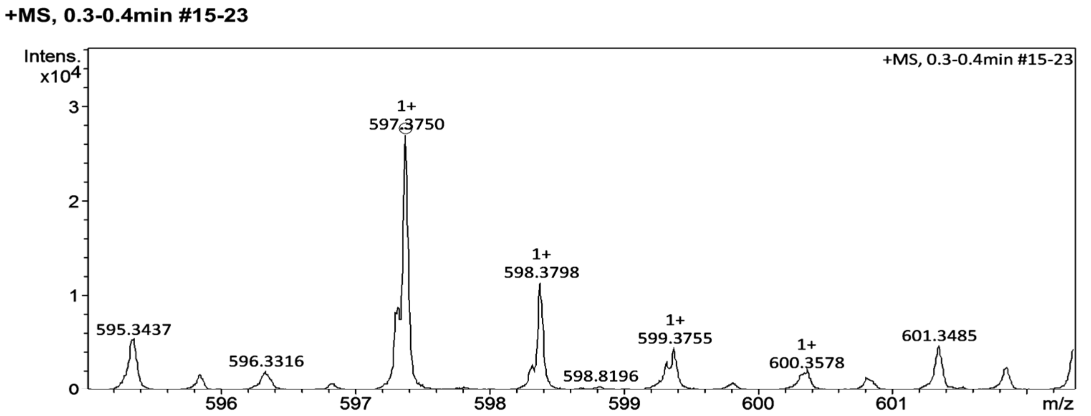

Supplementary Figure 45. HRMS of **15**

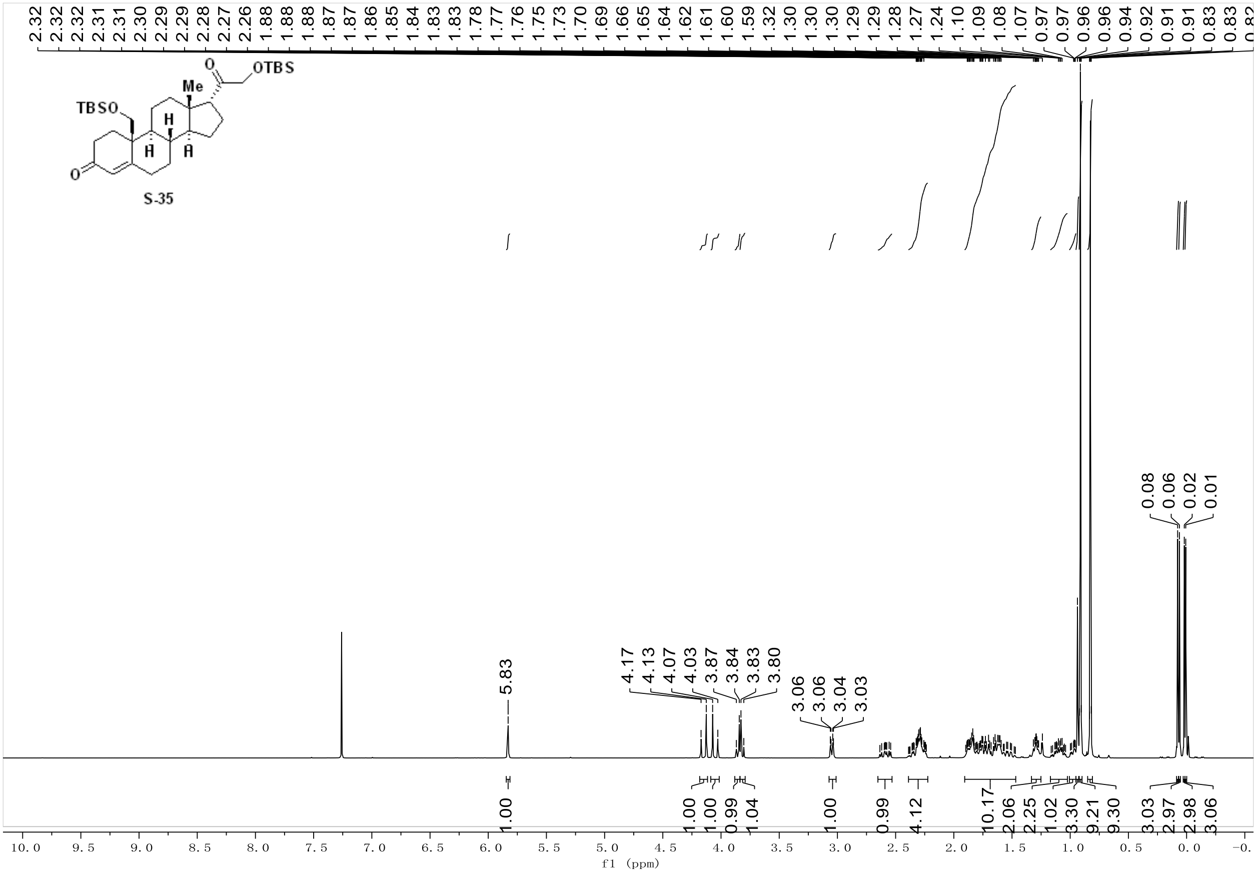

Supplementary Figure 46. ^{1}H NMR of **S-35**

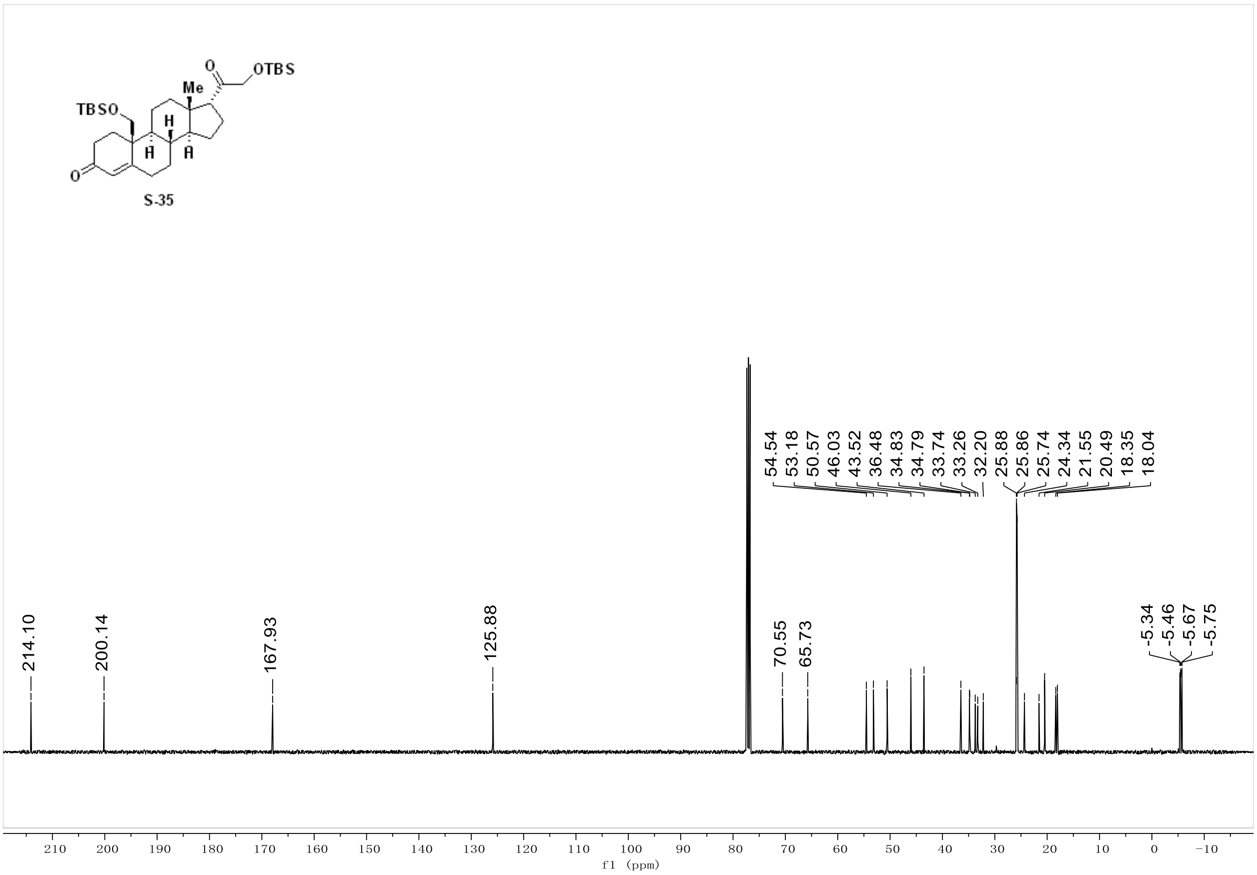

Supplementary Figure 47. ^{13}C NMR of **S-35**

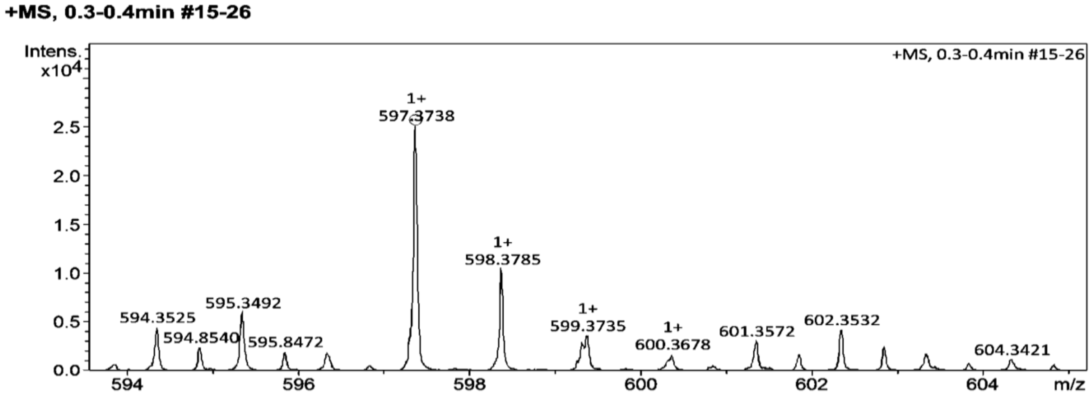

Supplementary Figure 48. HRMS of **S-35**

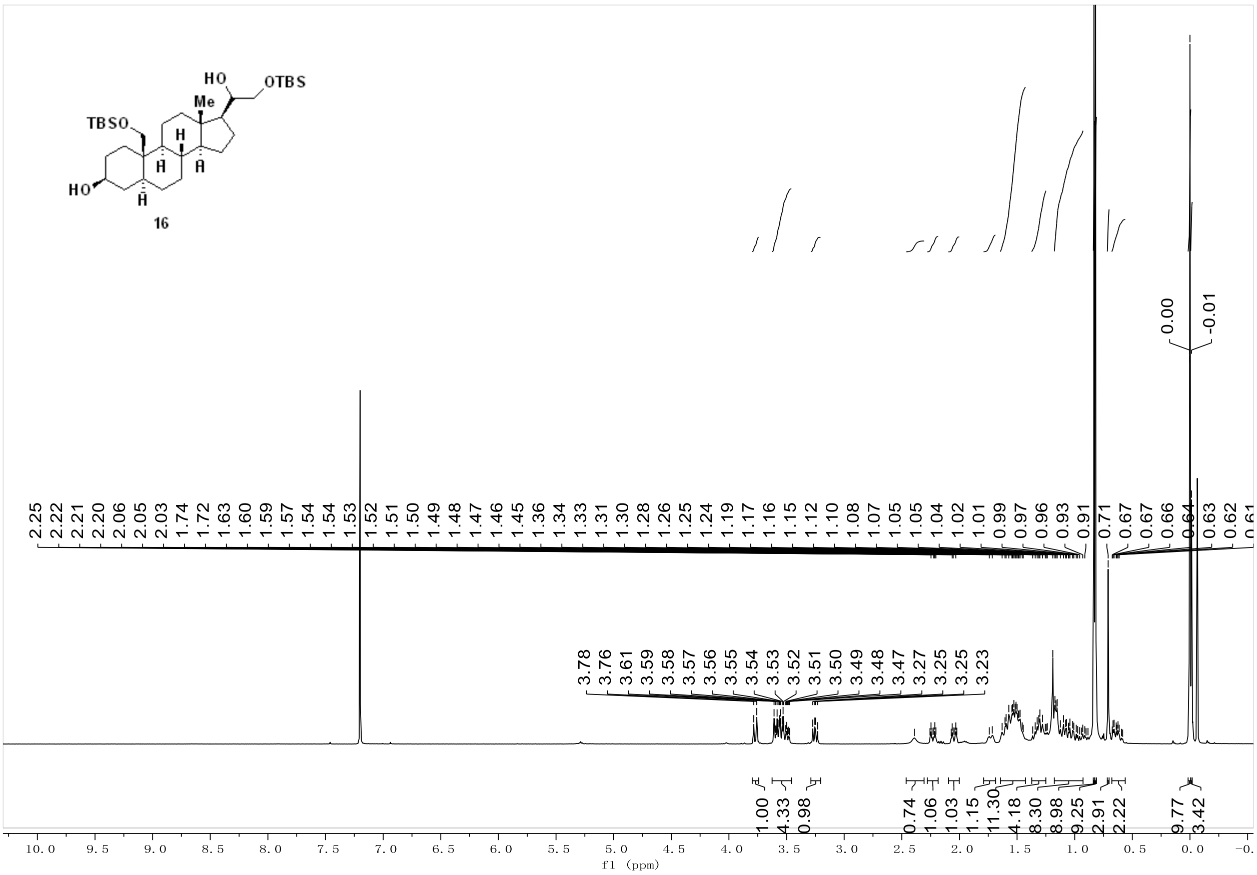

Supplementary Figure 49. ^{1}H NMR of **16**

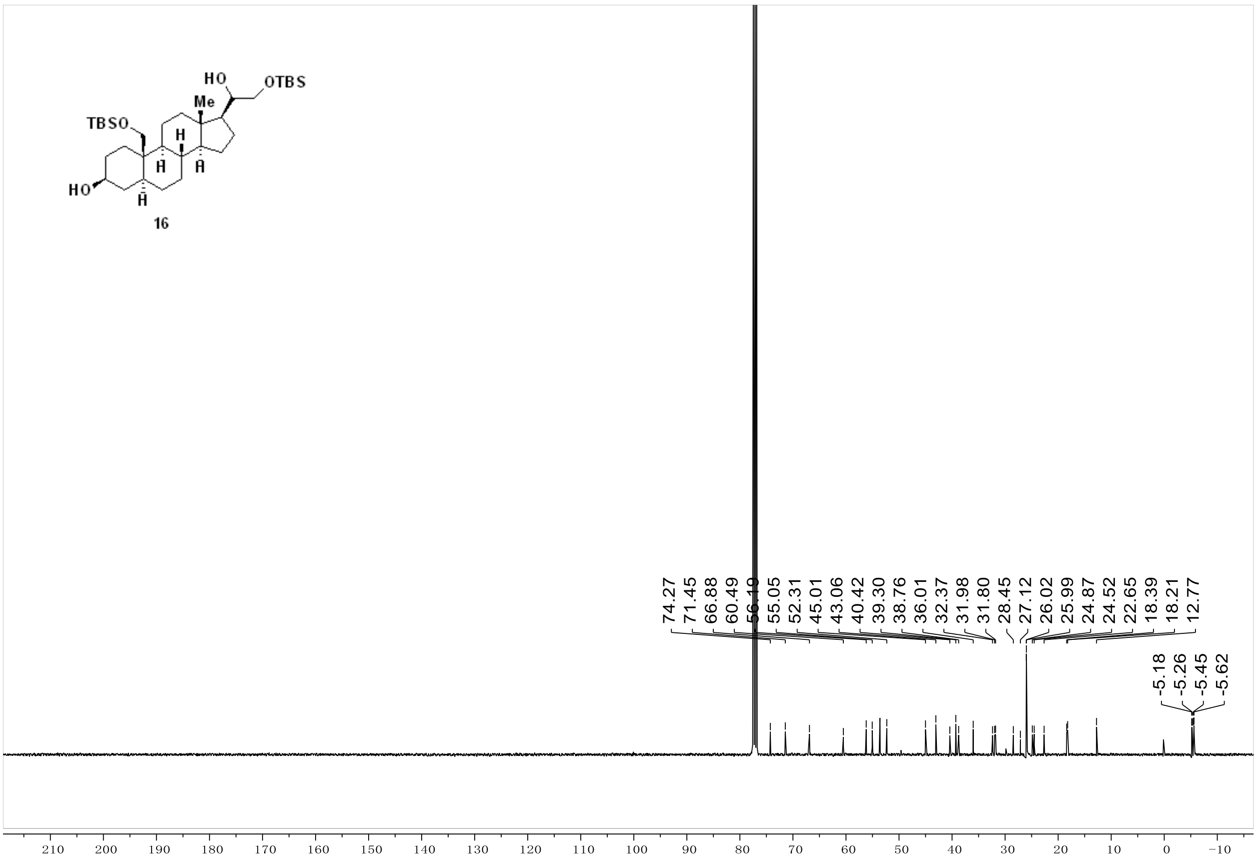

Supplementary Figure 50. ^{13}C NMR of **16**

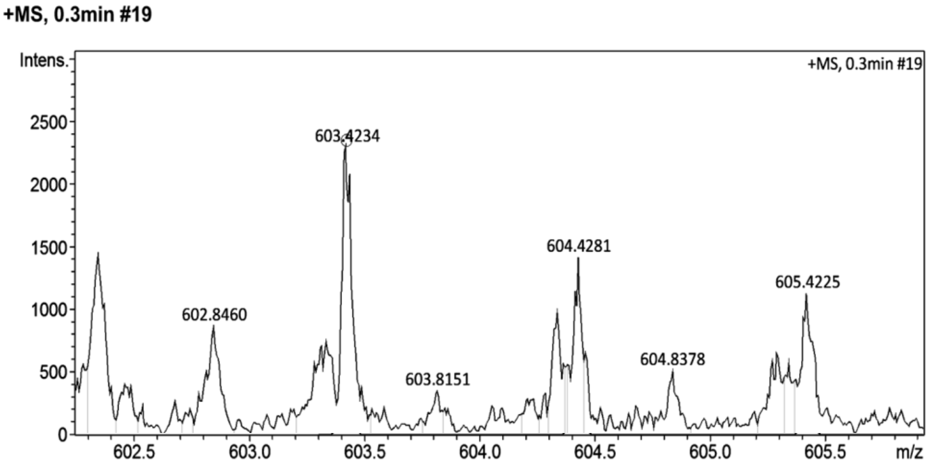

Supplementary Figure 51. HRMS of **16**

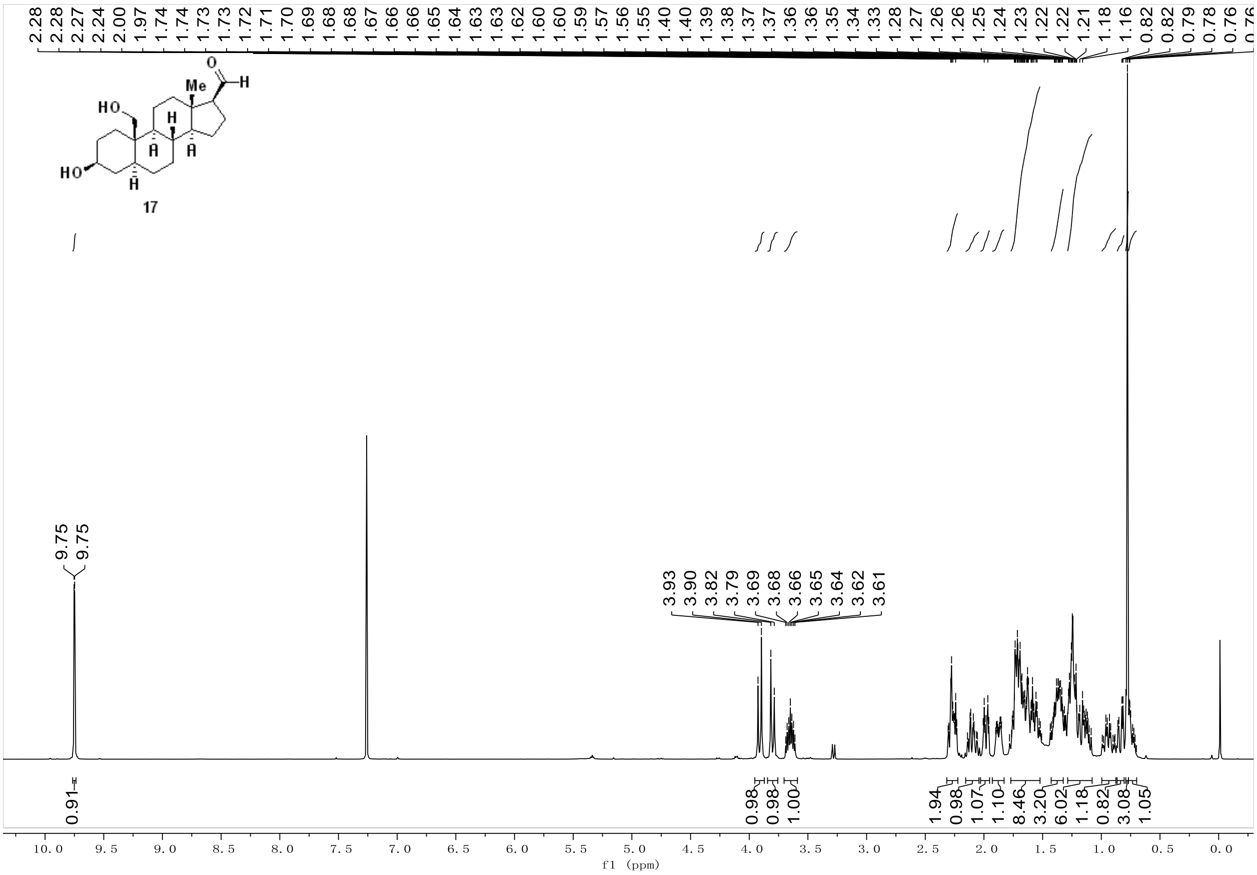

Supplementary Figure 52. ^{1}H NMR of **17**

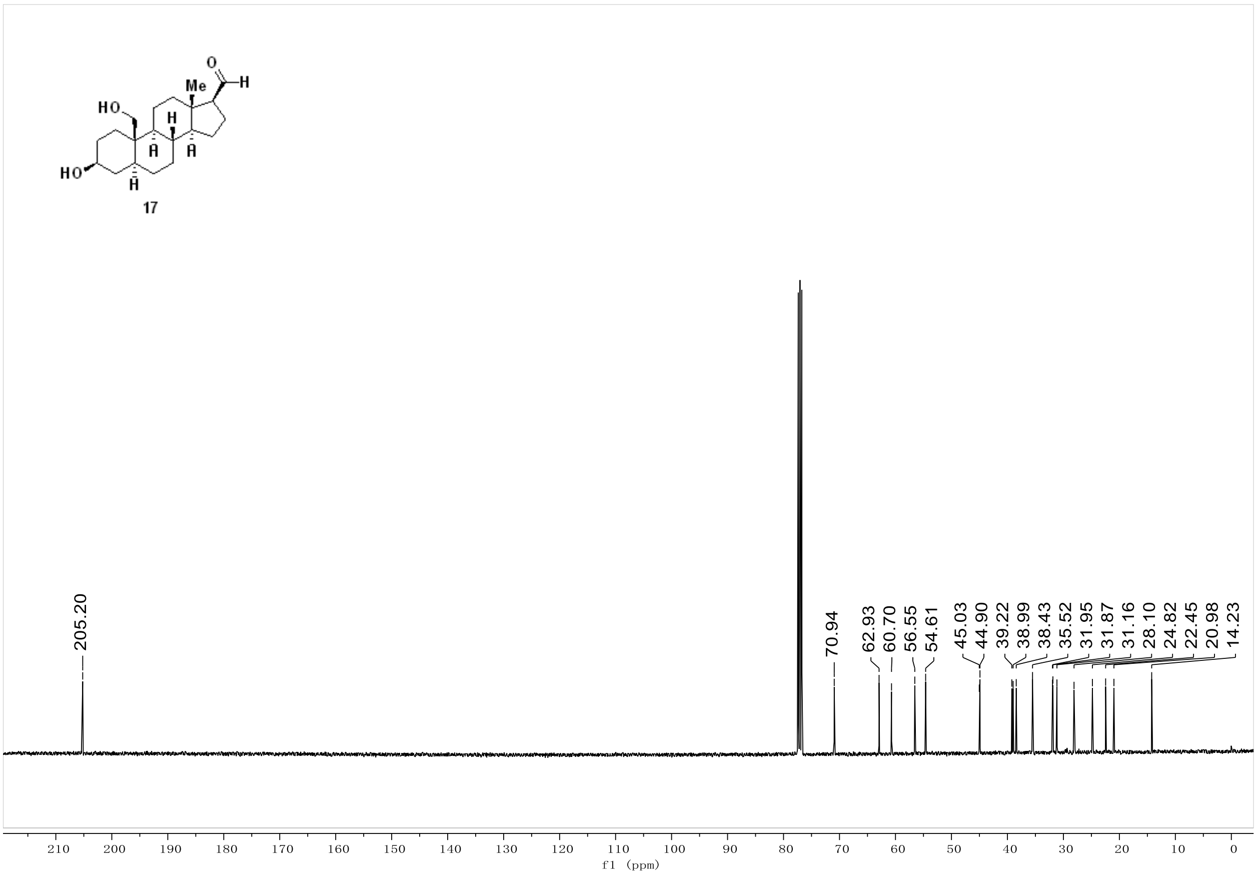

Supplementary Figure 53. ^{13}C NMR of **17**

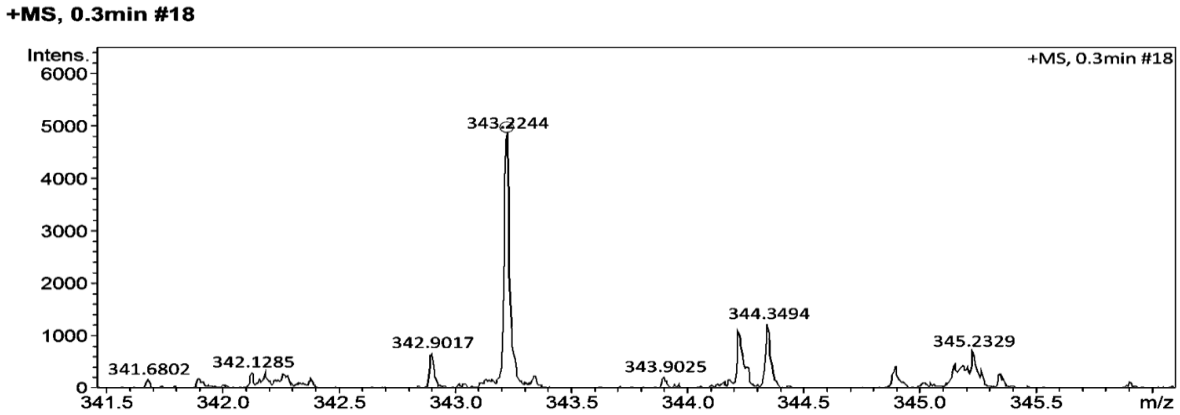

Supplementary Figure 54. HRMS of **17**

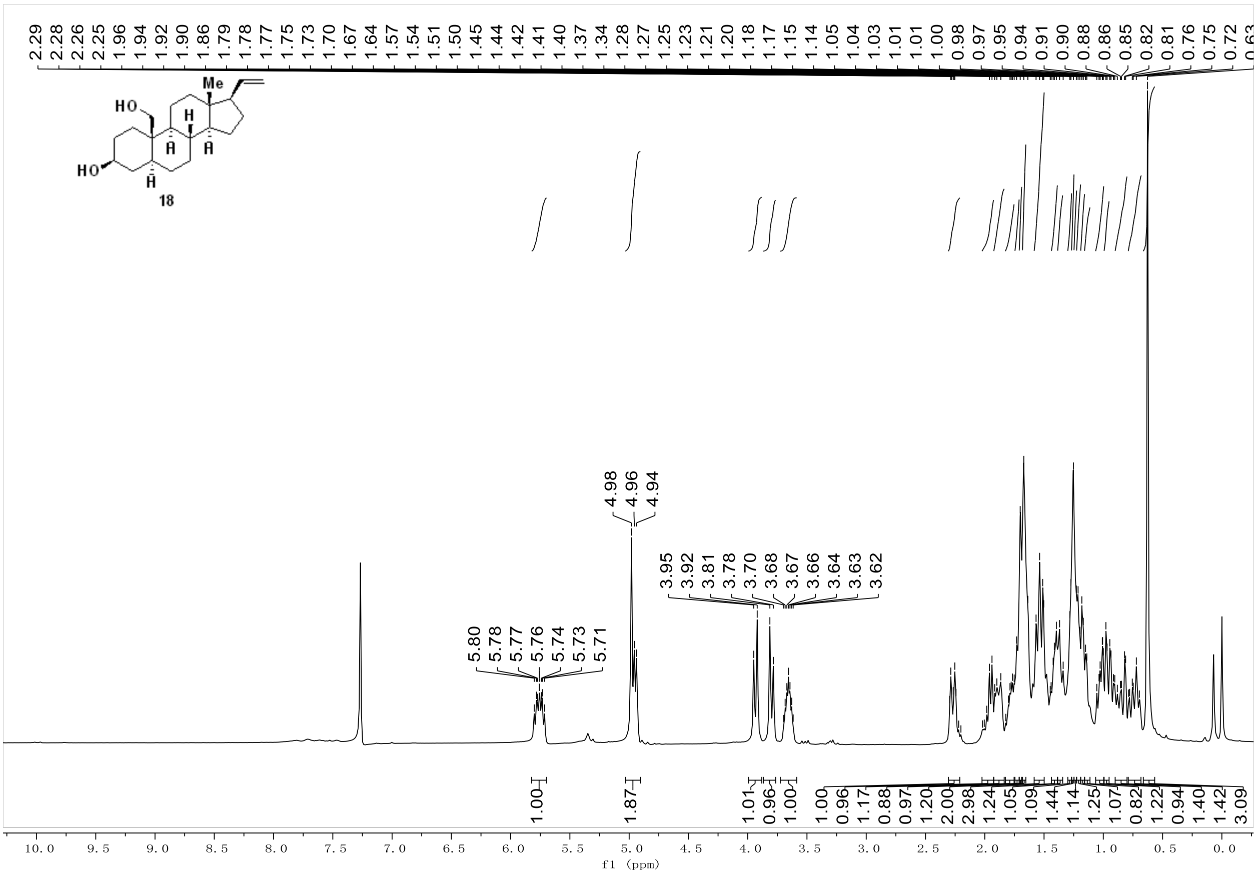

Supplementary Figure 55. ^{1}H NMR of **18**

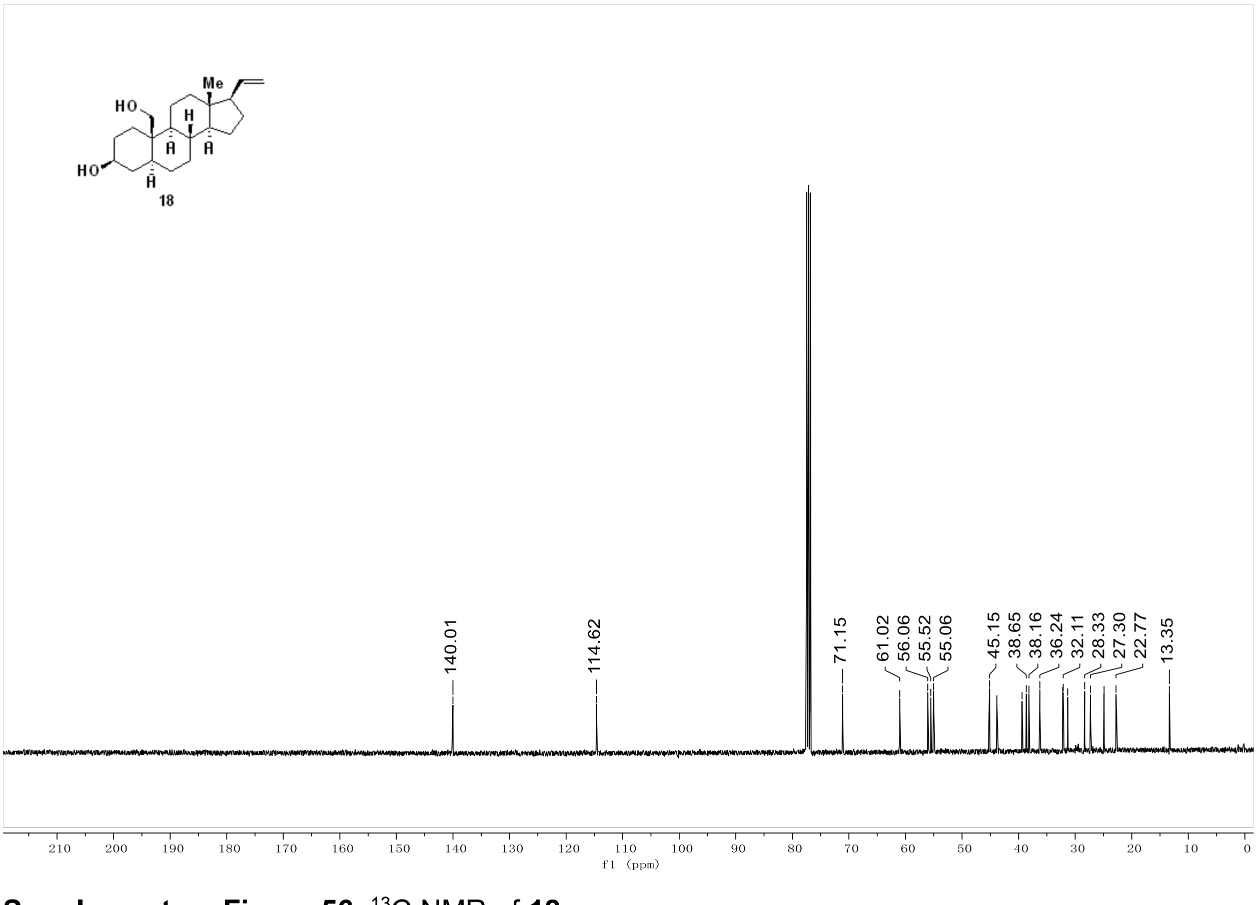

Supplementary Figure 56. ^{13}C NMR of **18**

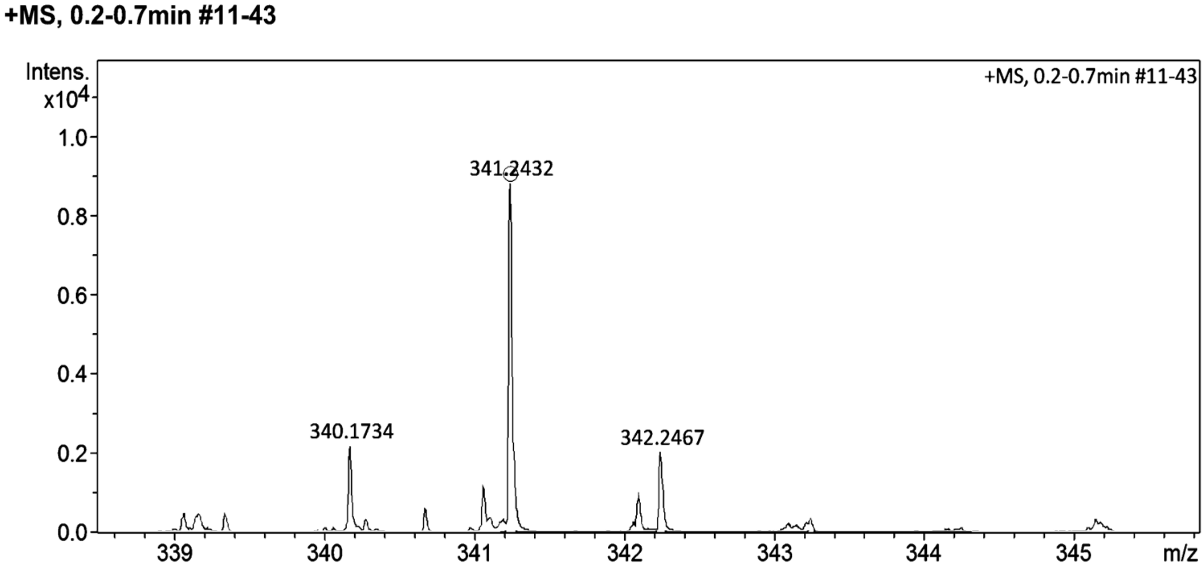

Supplementary Figure 57. HRMS of **18**

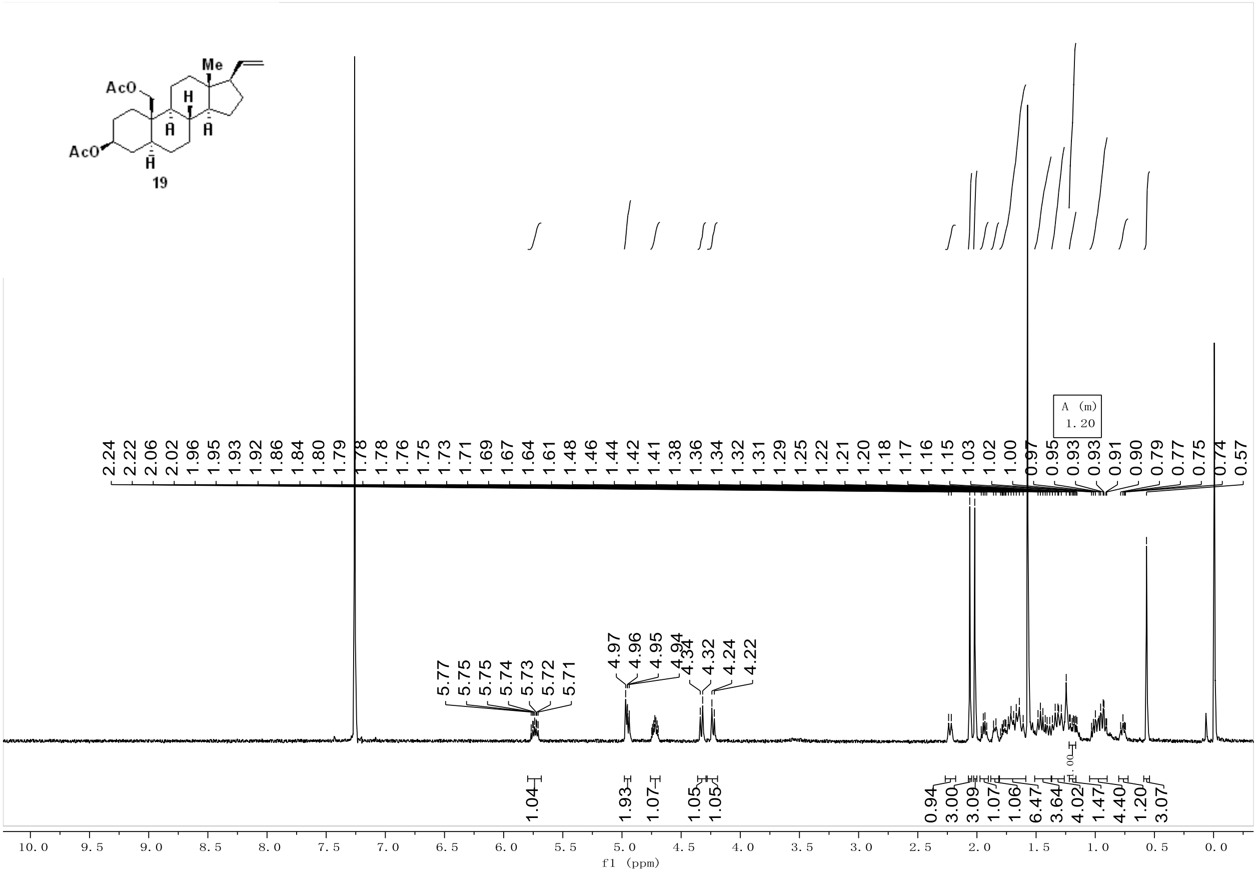

Supplementary Figure 58. ^{1}H NMR of **19**

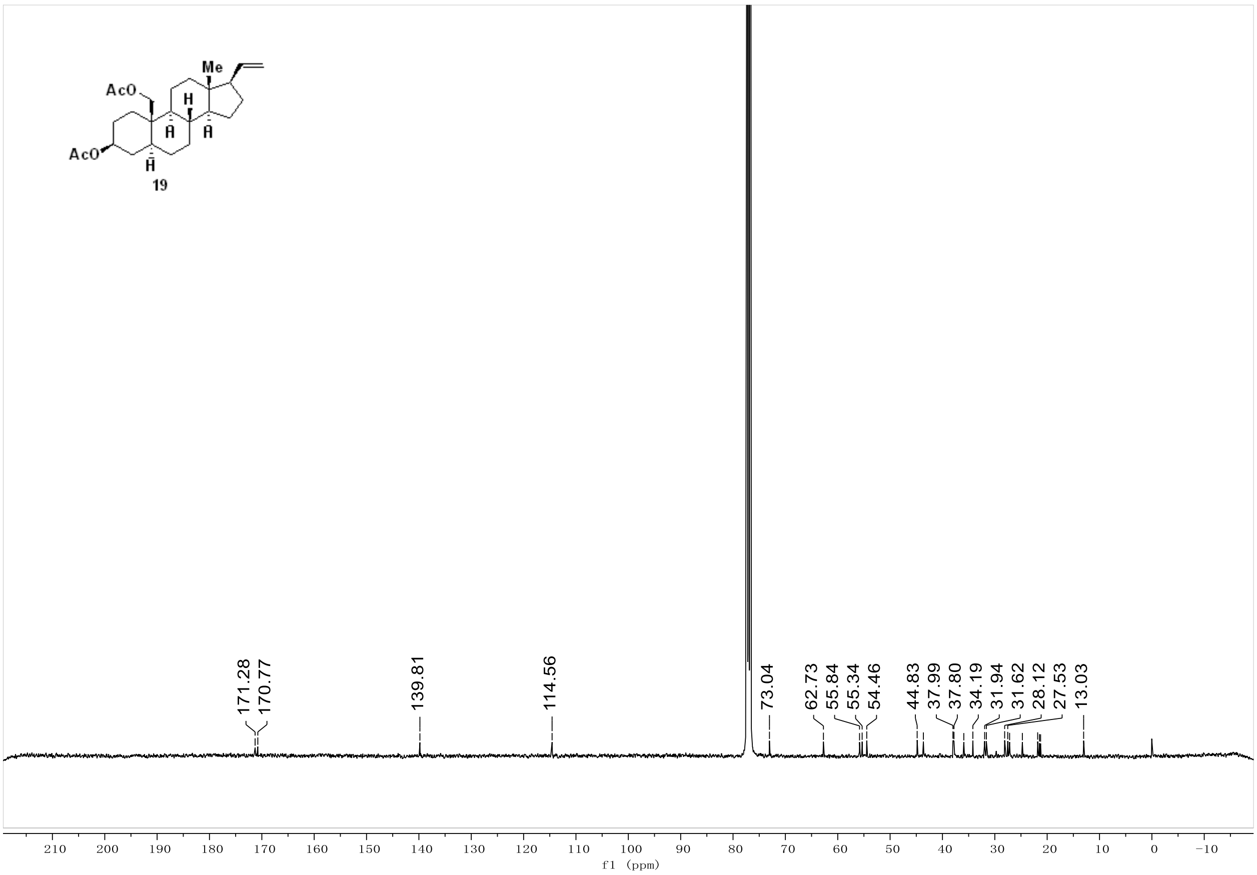

Supplementary Figure 59. ^{13}C NMR of **19**

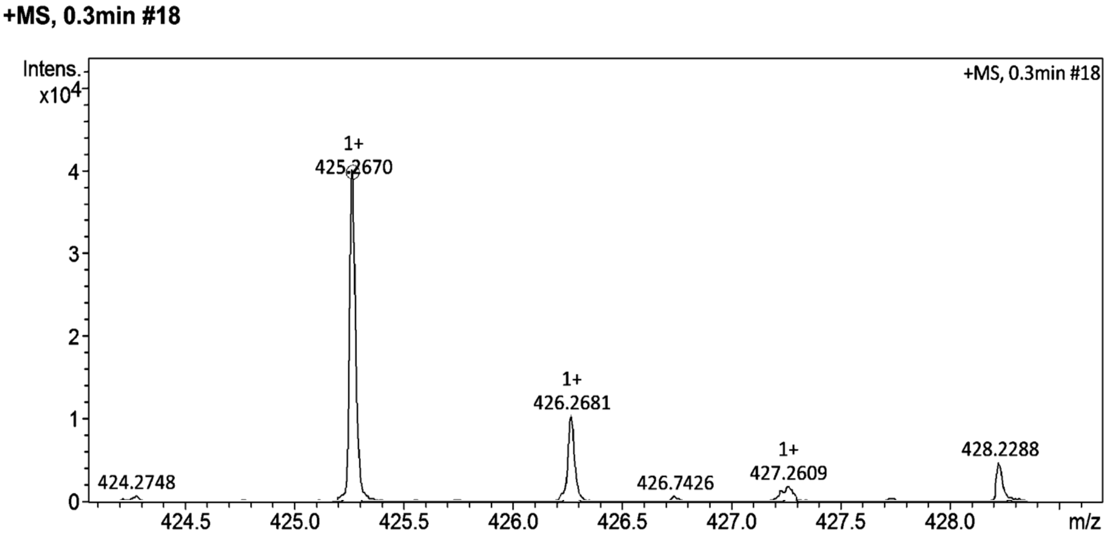

Supplementary Figure 60. HRMS of **19**

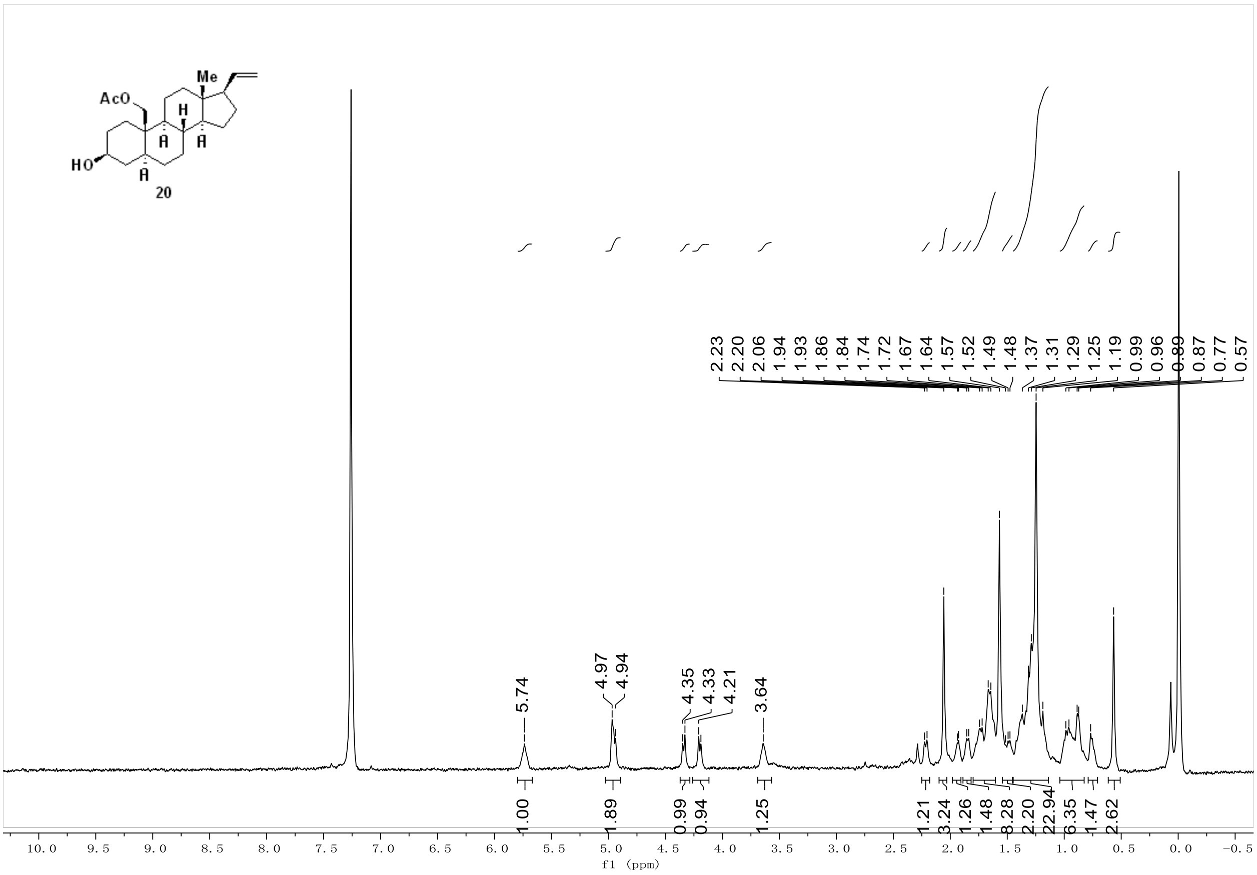

Supplementary Figure 61. ^{1}H NMR of **20**

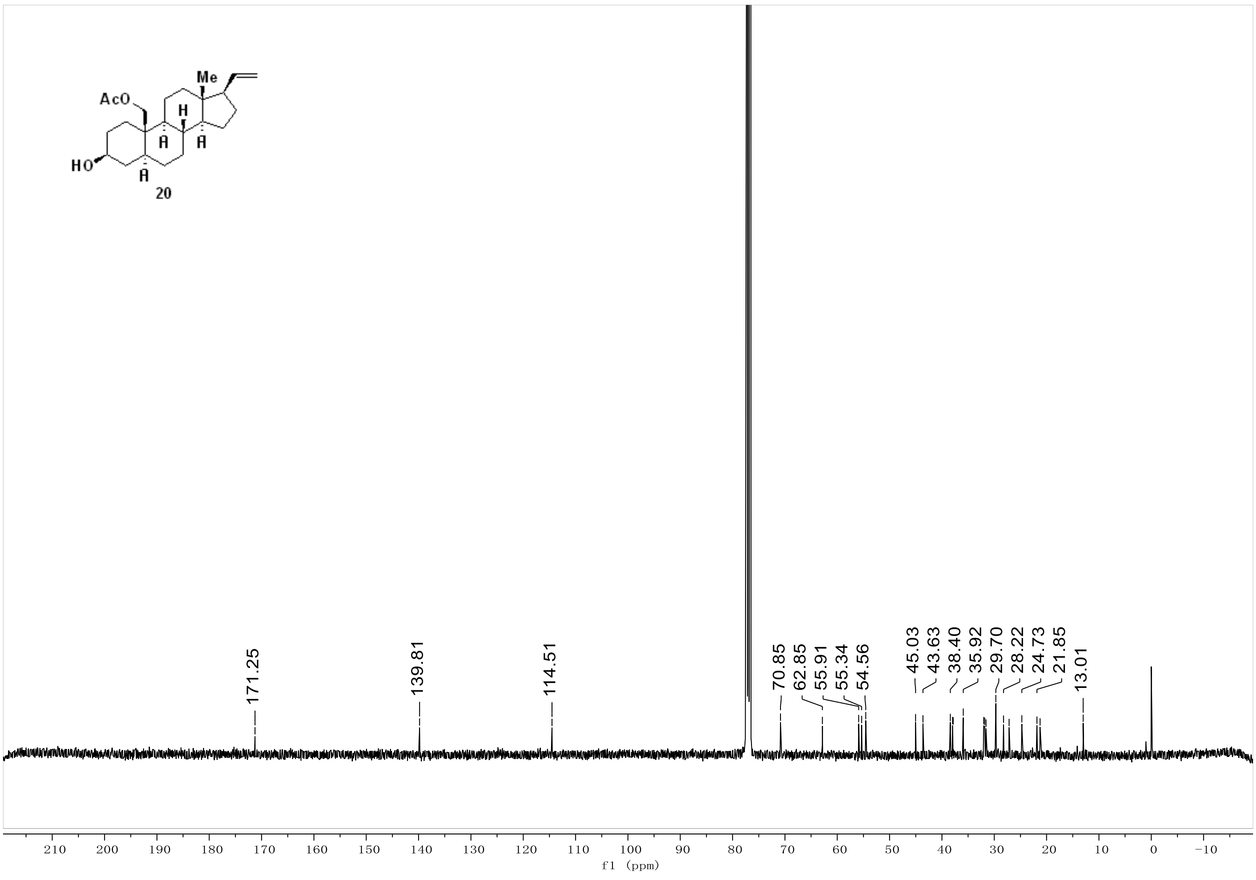

Supplementary Figure 62. ^{13}C NMR of **20**

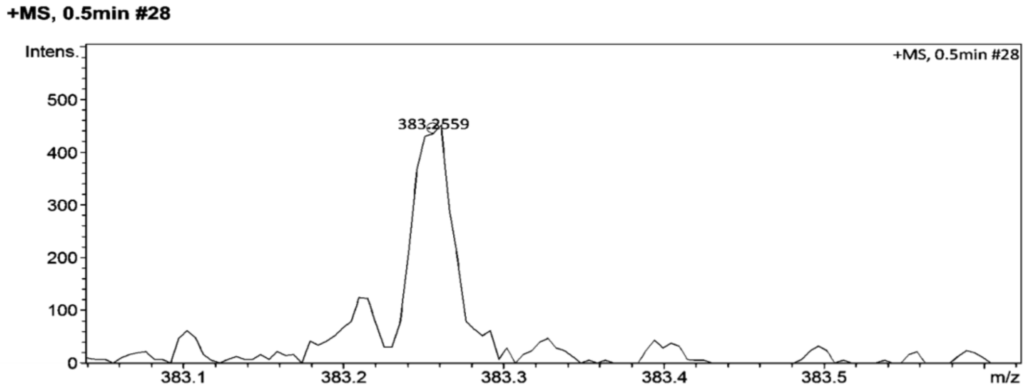

Supplementary Figure 63. HRMS of **20**

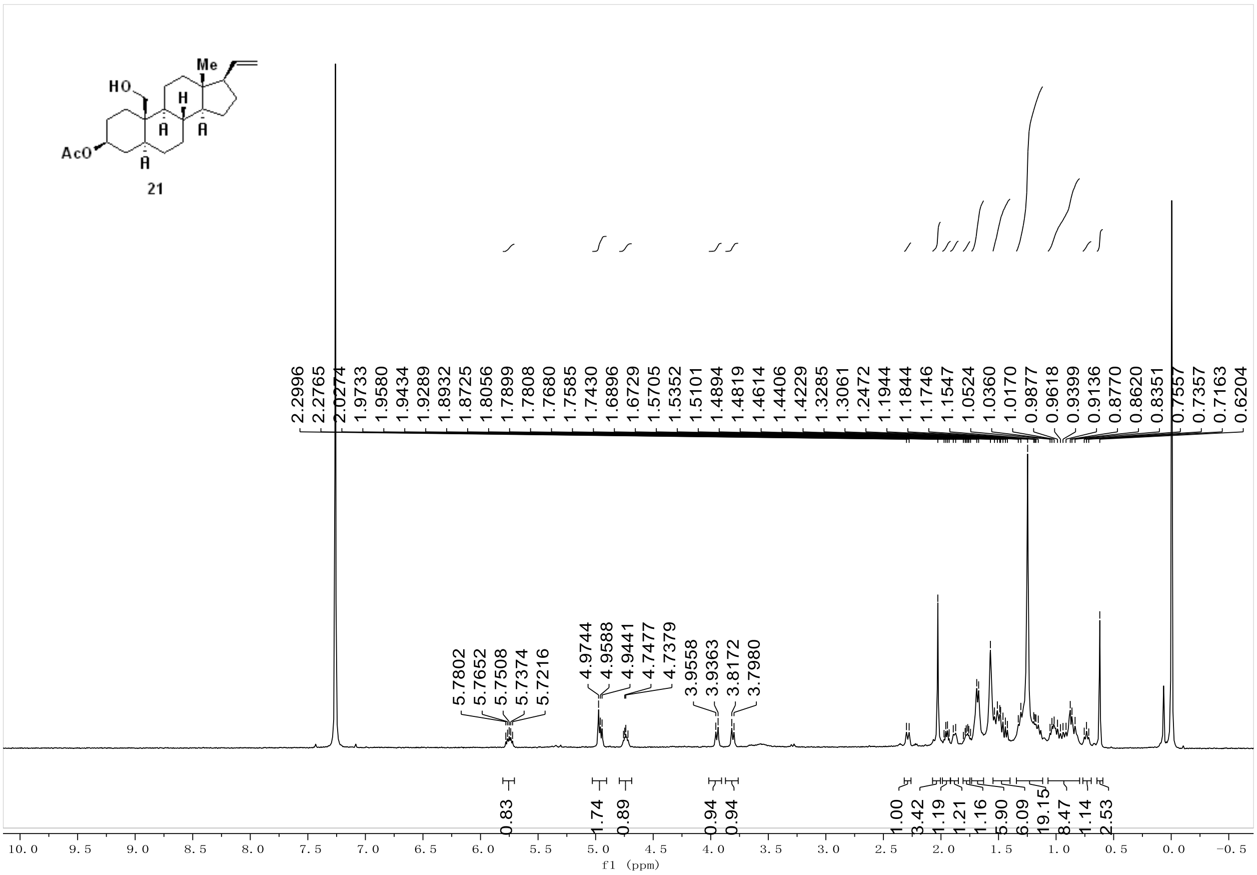

Supplementary Figure 64. ^{1}H NMR of **21**

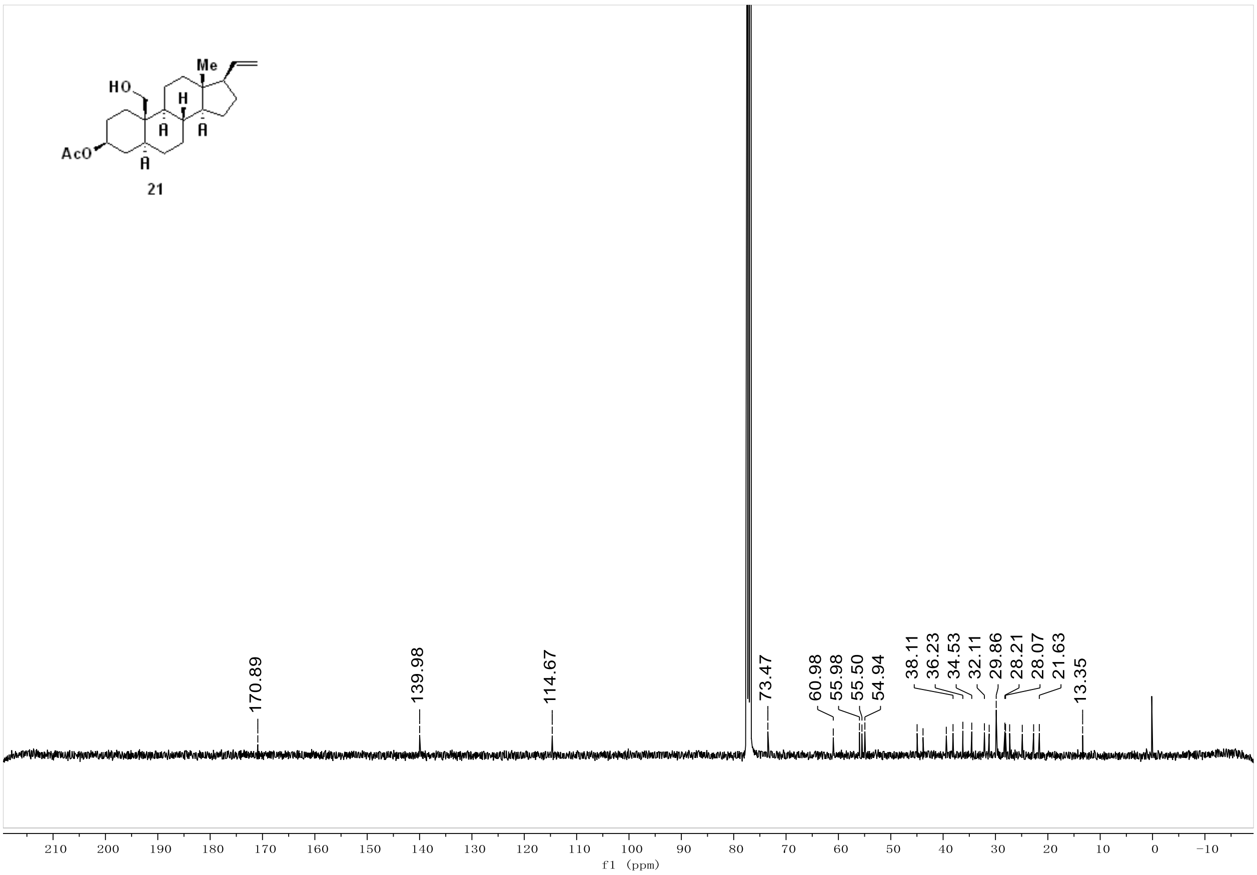

Supplementary Figure 65. ^{13}C NMR of **21**

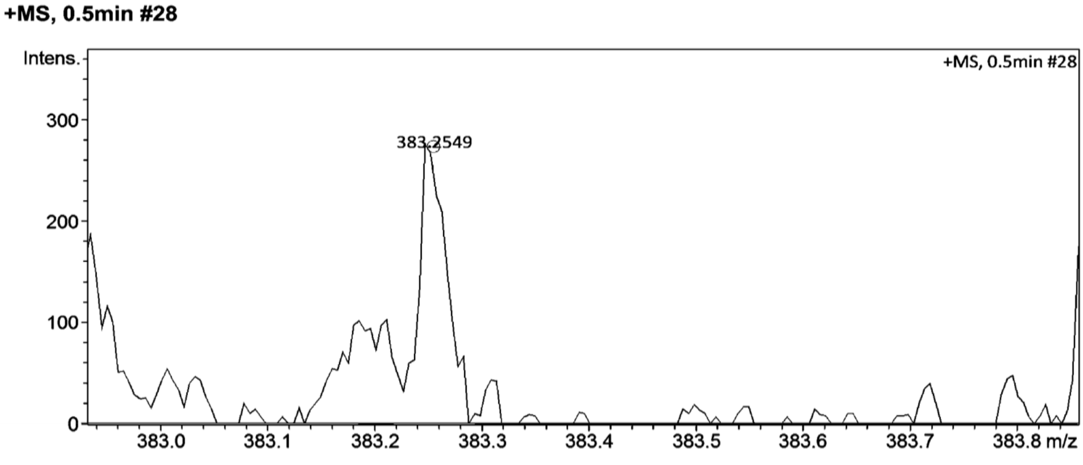

Supplementary Figure 66. HRMS of **21**

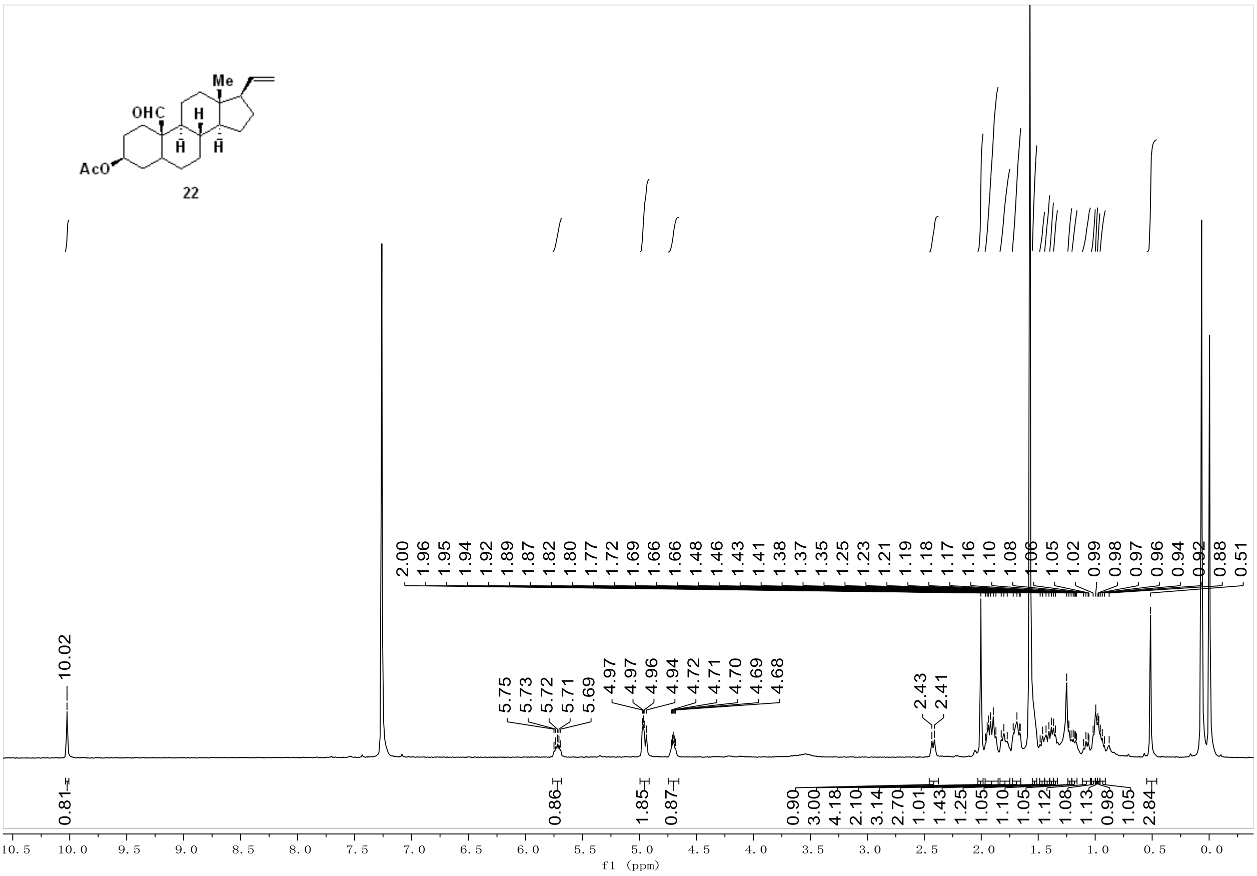

Supplementary Figure 67. ^{1}H NMR of **22**

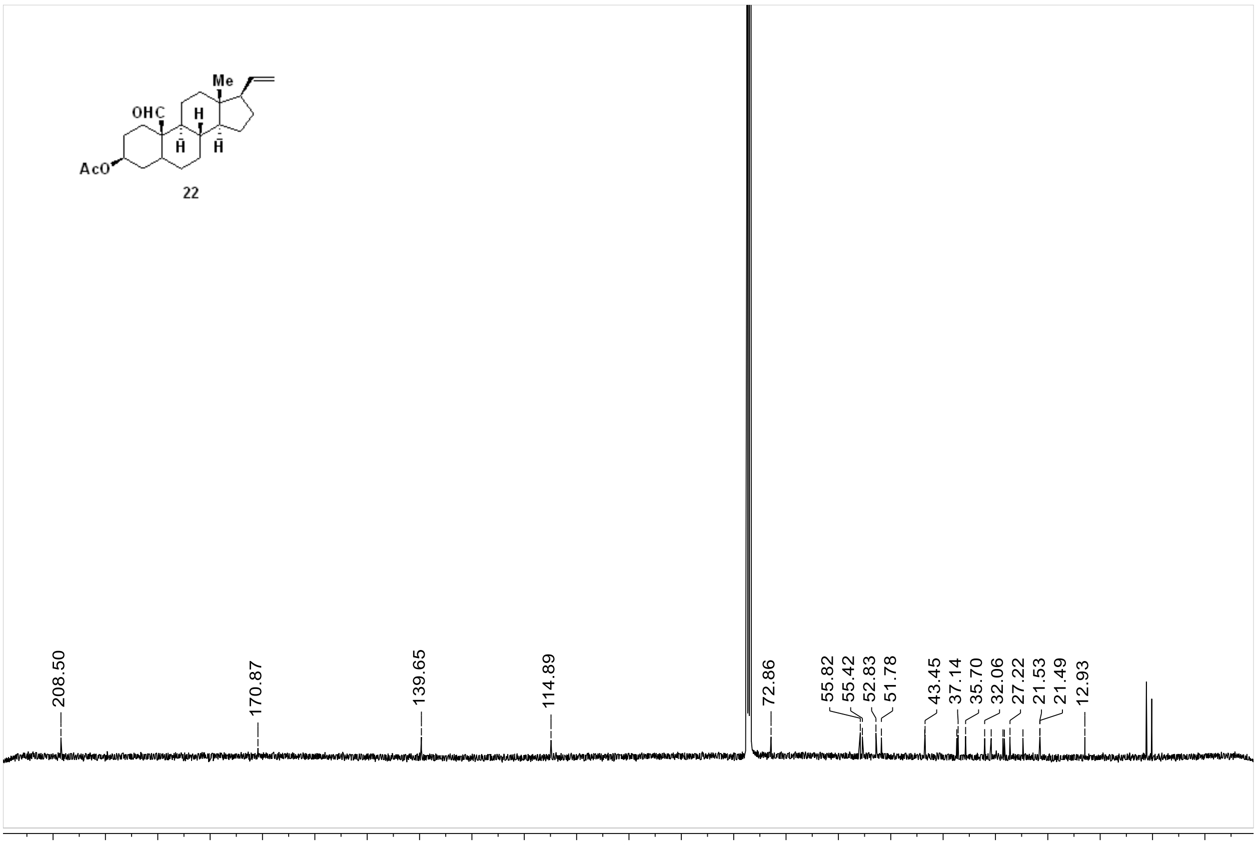

Supplementary Figure 68. ^{13}C NMR of **22**

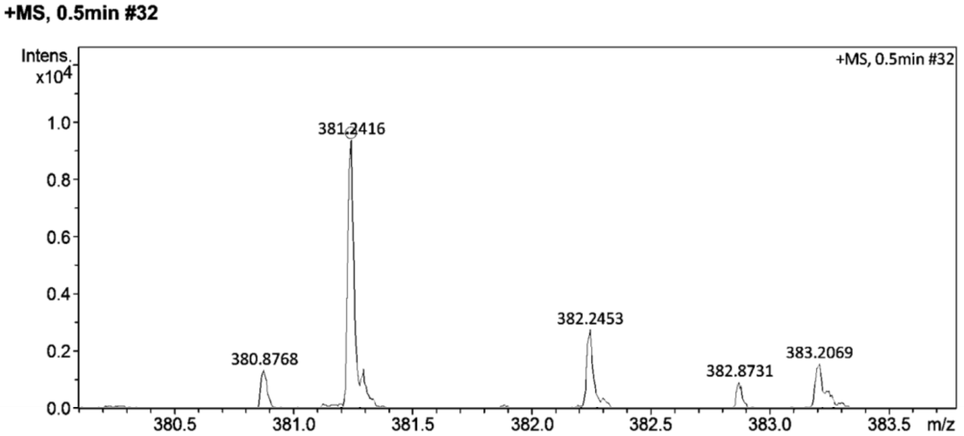

Supplementary Figure 69. HRMS of **22**

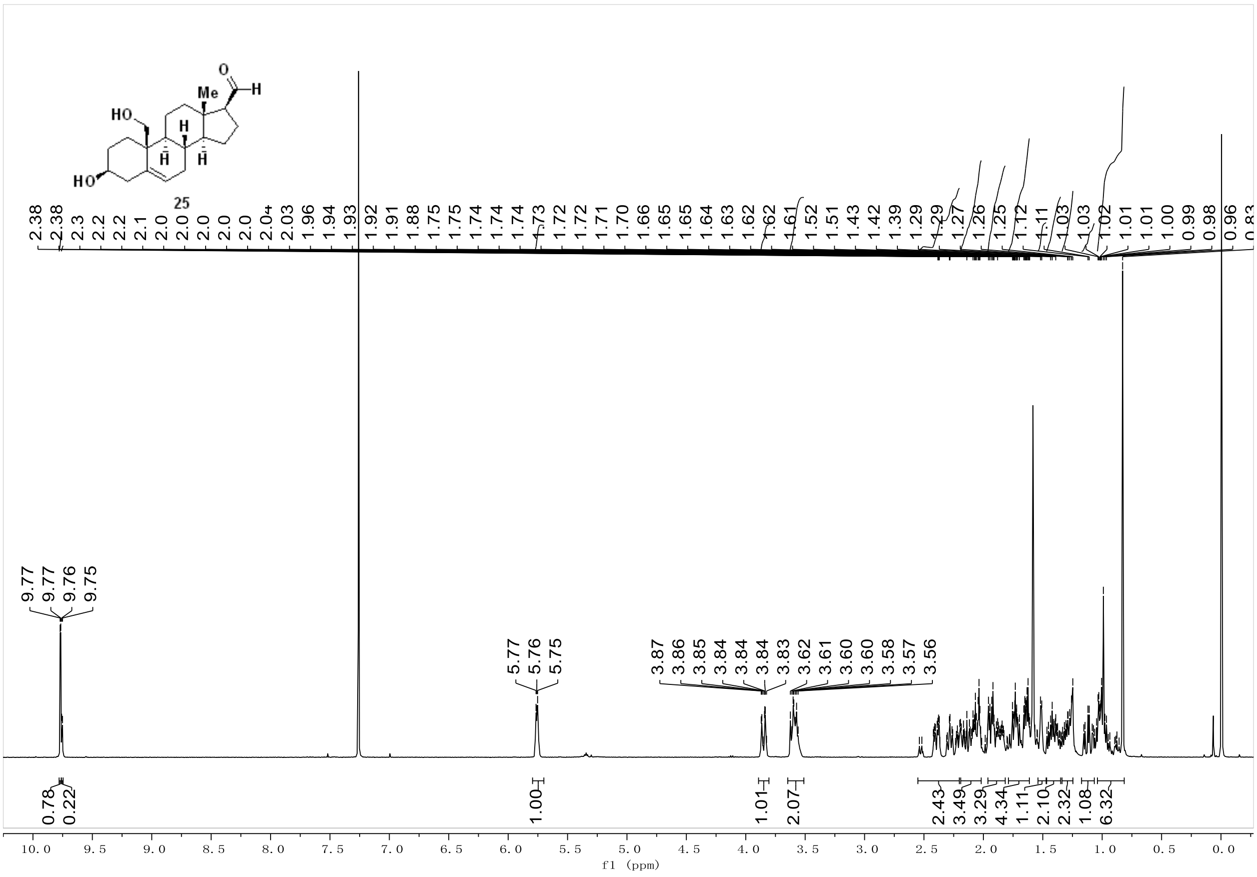

Supplementary Figure 70. ^{1}H NMR of **25**

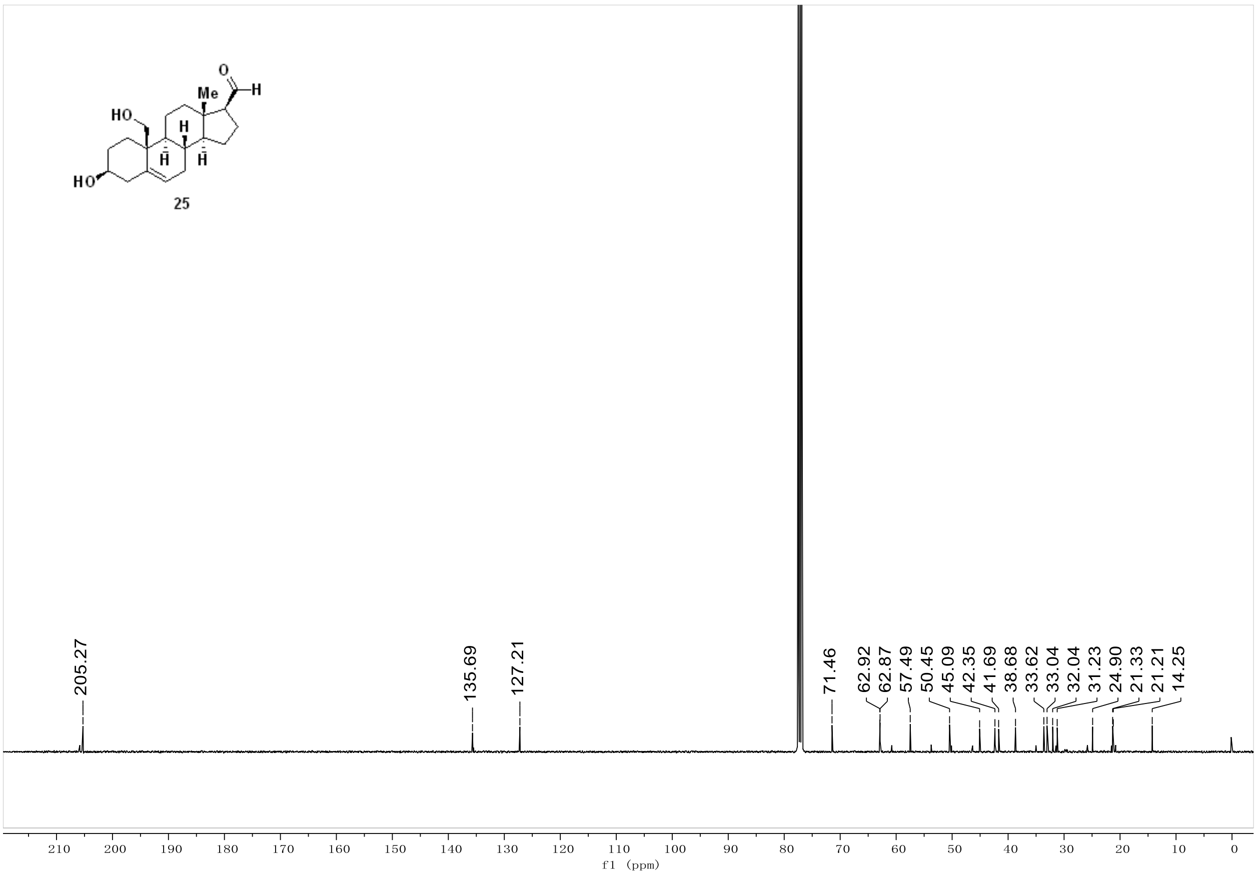

Supplementary Figure 71. ^{13}C NMR of **25**

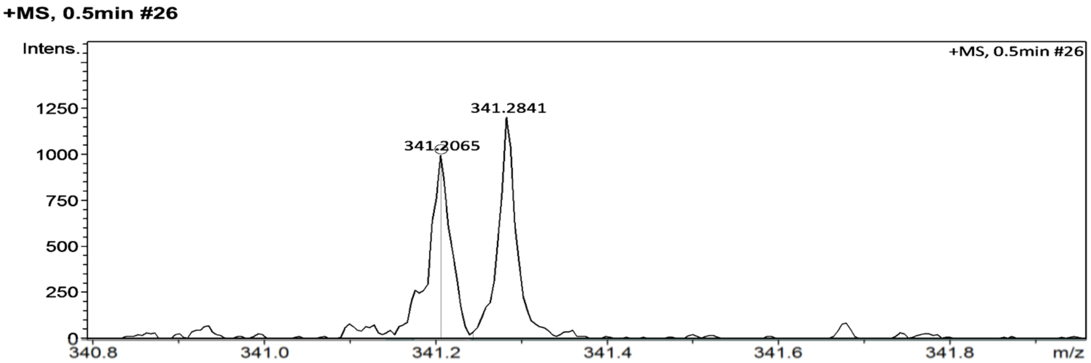

Supplementary Figure 72. HRMS of **26**

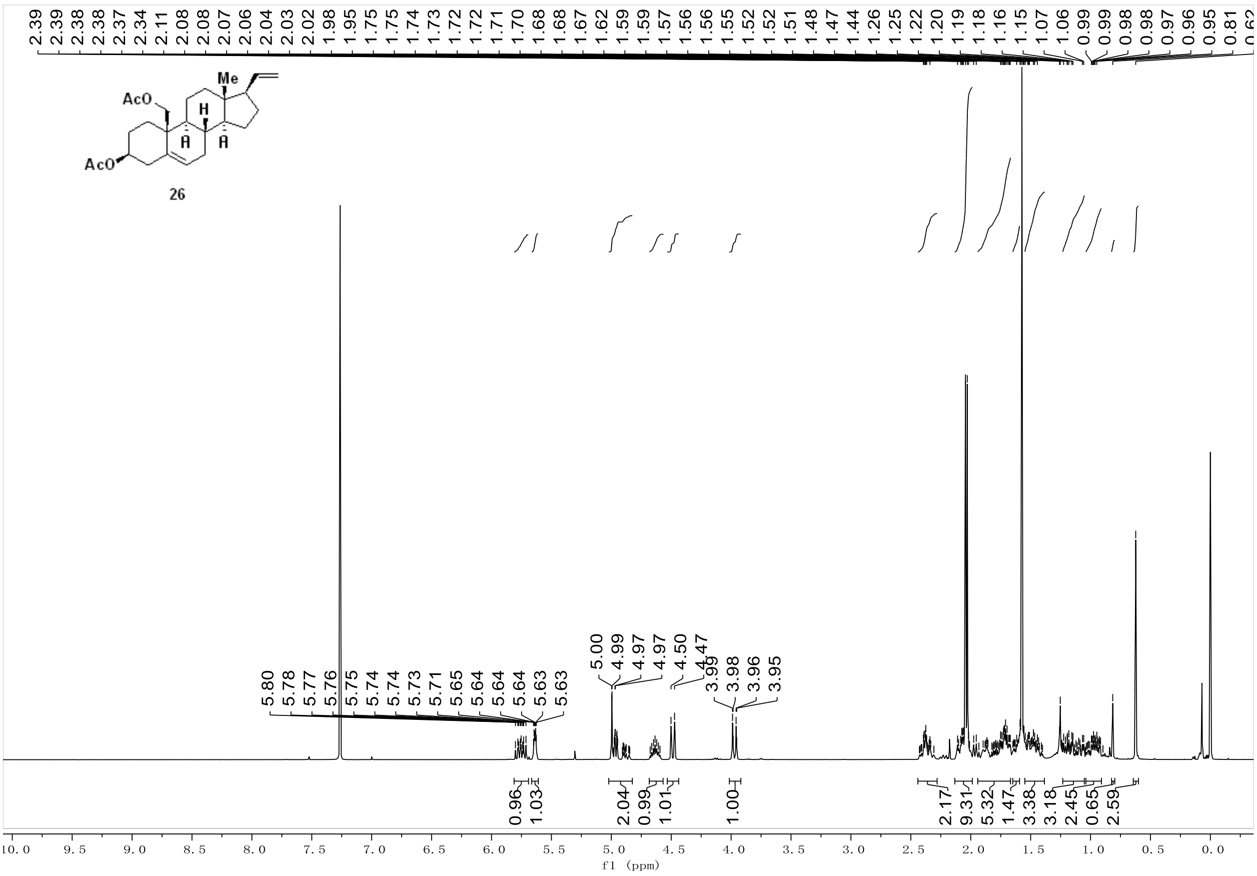

Supplementary Figure 73. ^{1}H NMR of **26**

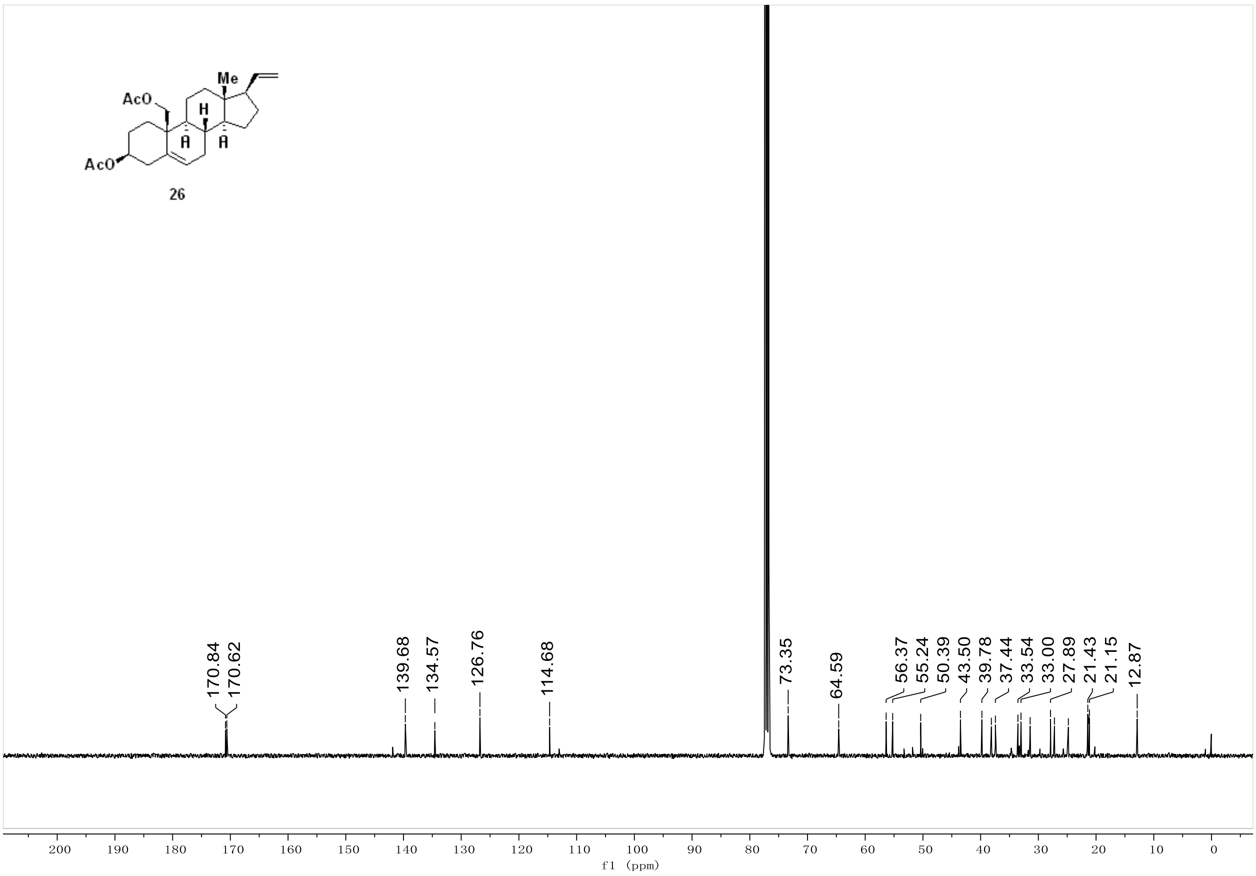

Supplementary Figure 74. ^{13}C NMR of **26**

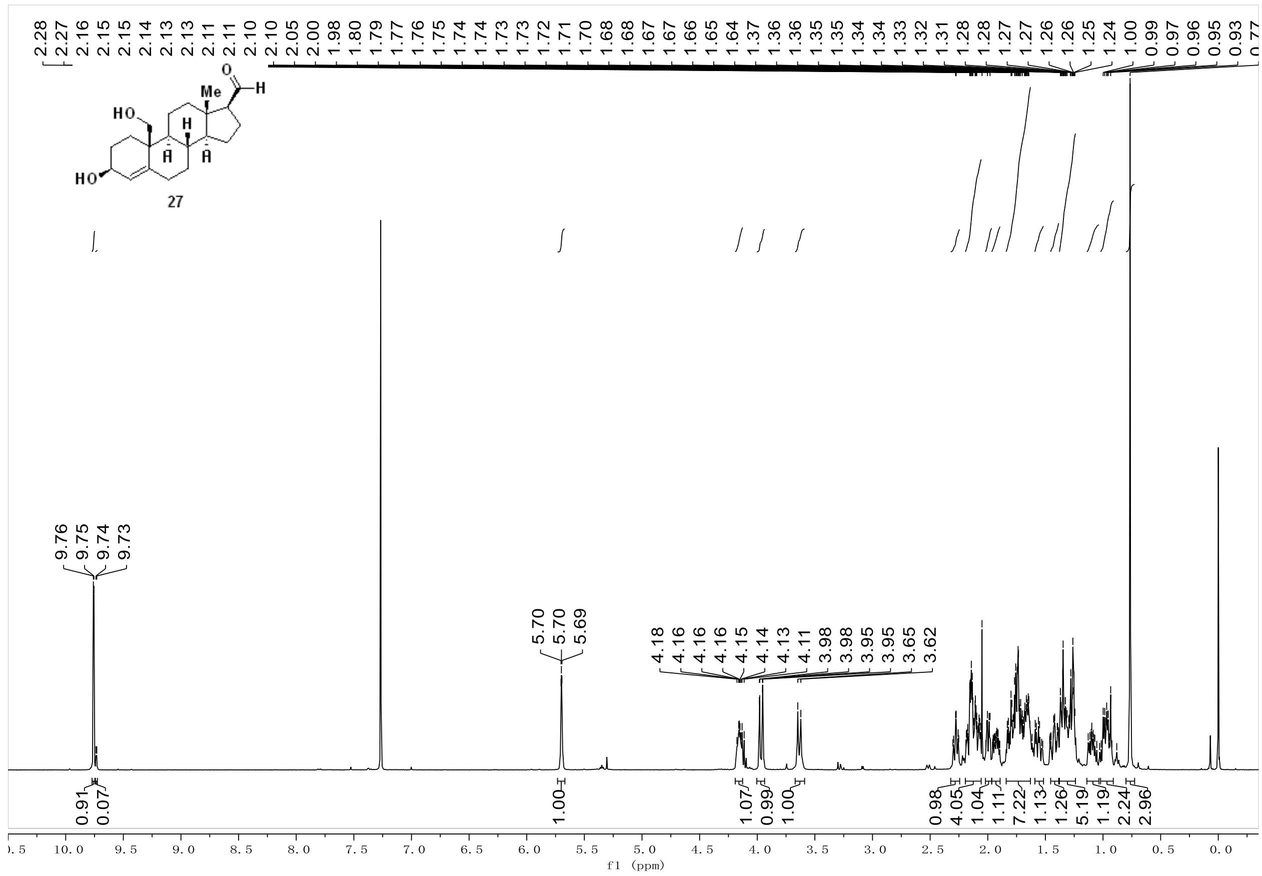

Supplementary Figure 75. ^{1}H NMR of **27**

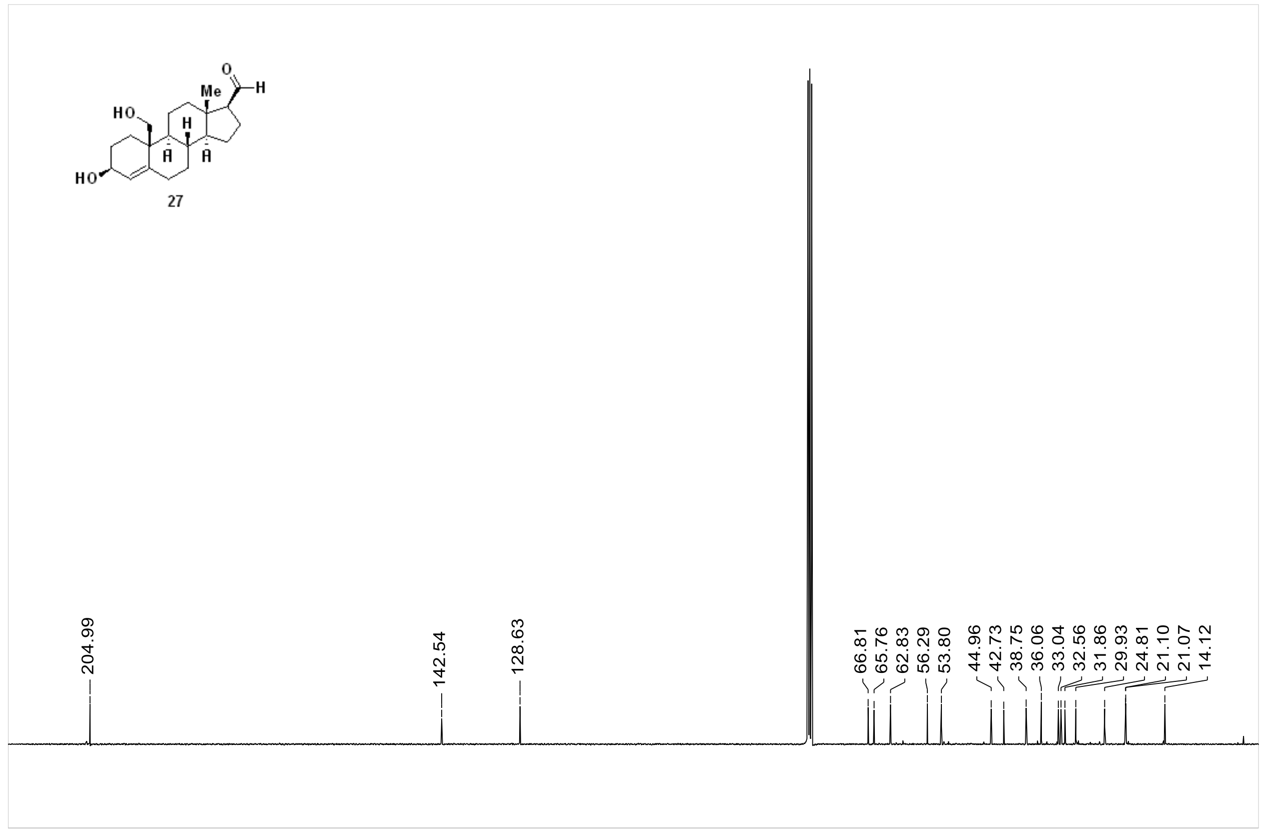

Supplementary Figure 76. ^{13}C NMR of **27**

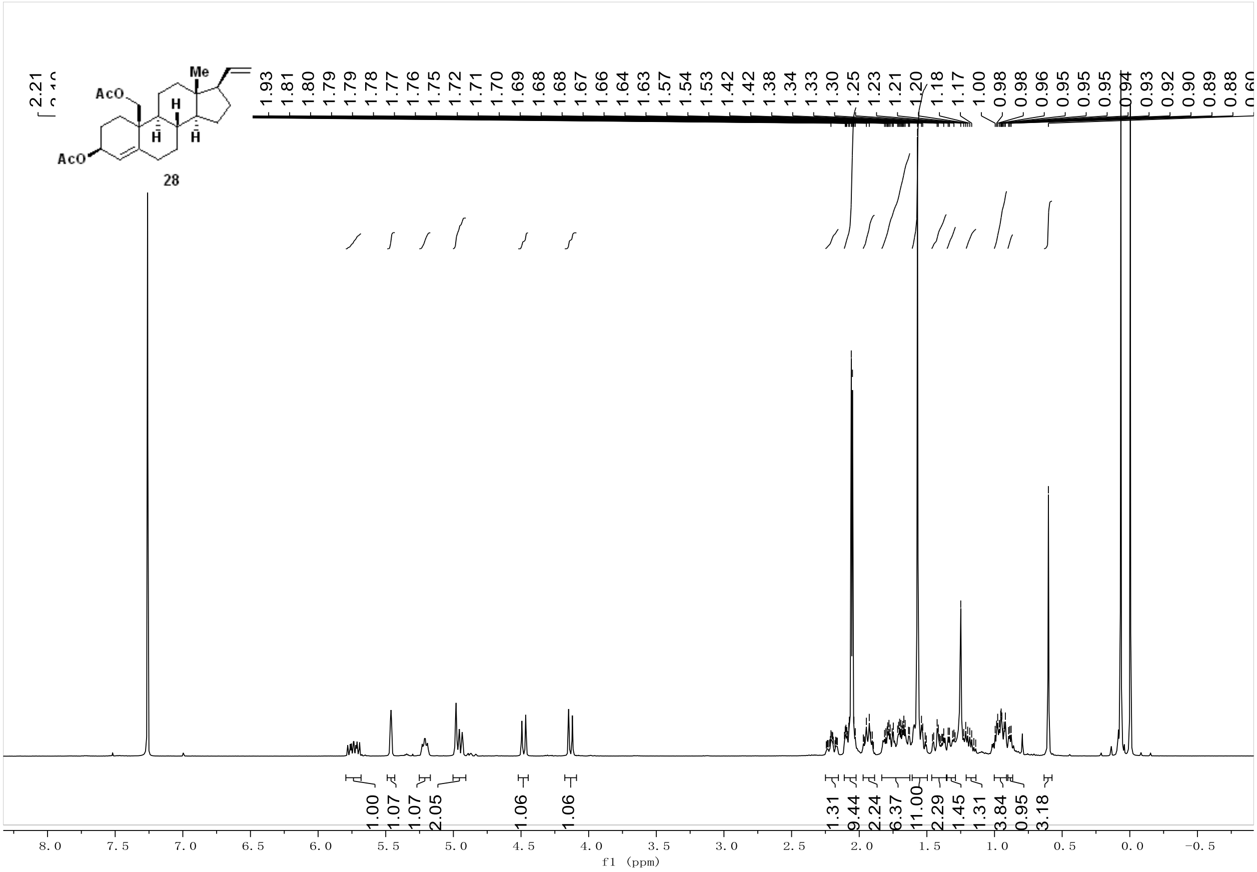

Supplementary Figure 77. ^{1}H NMR of **28**

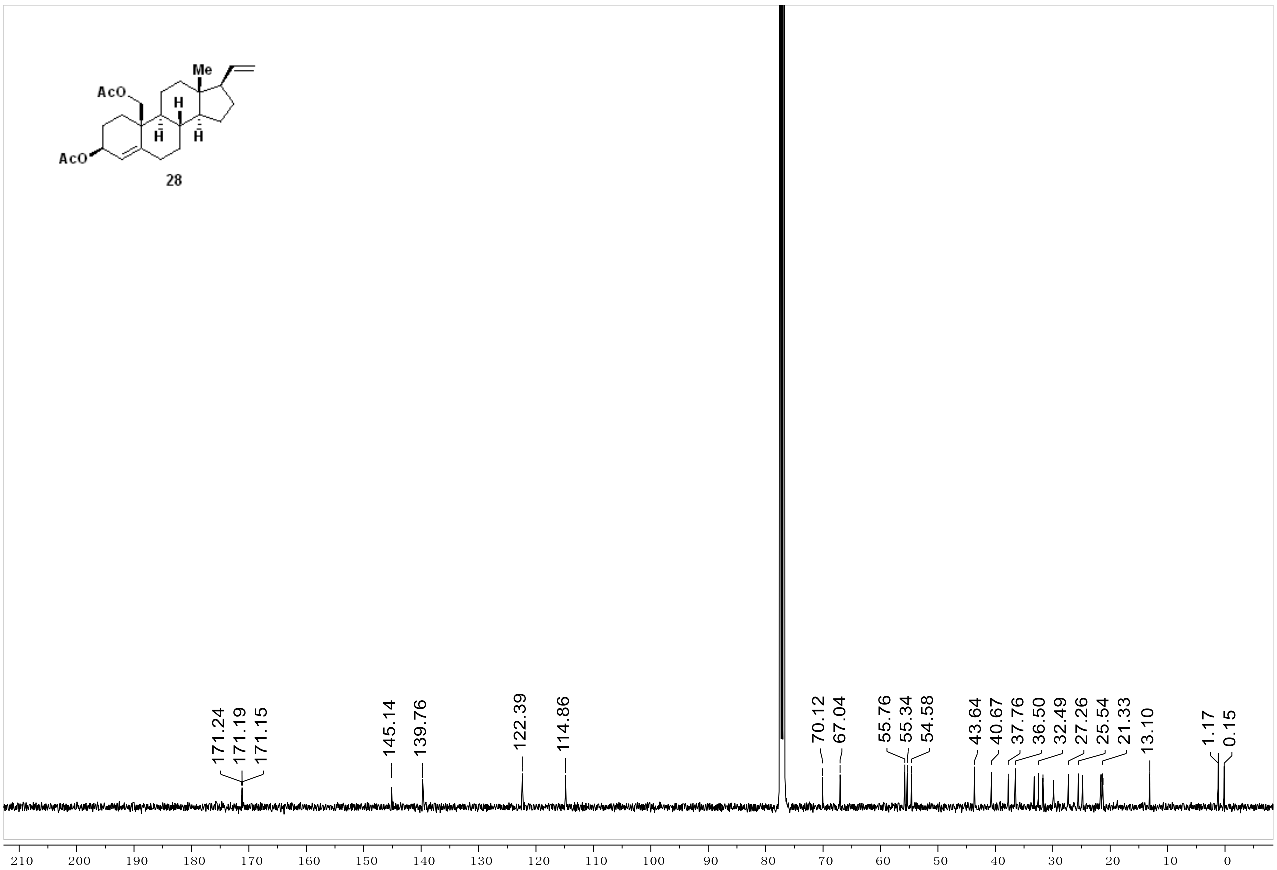

Supplementary Figure 78. ^{13}C NMR of **28**

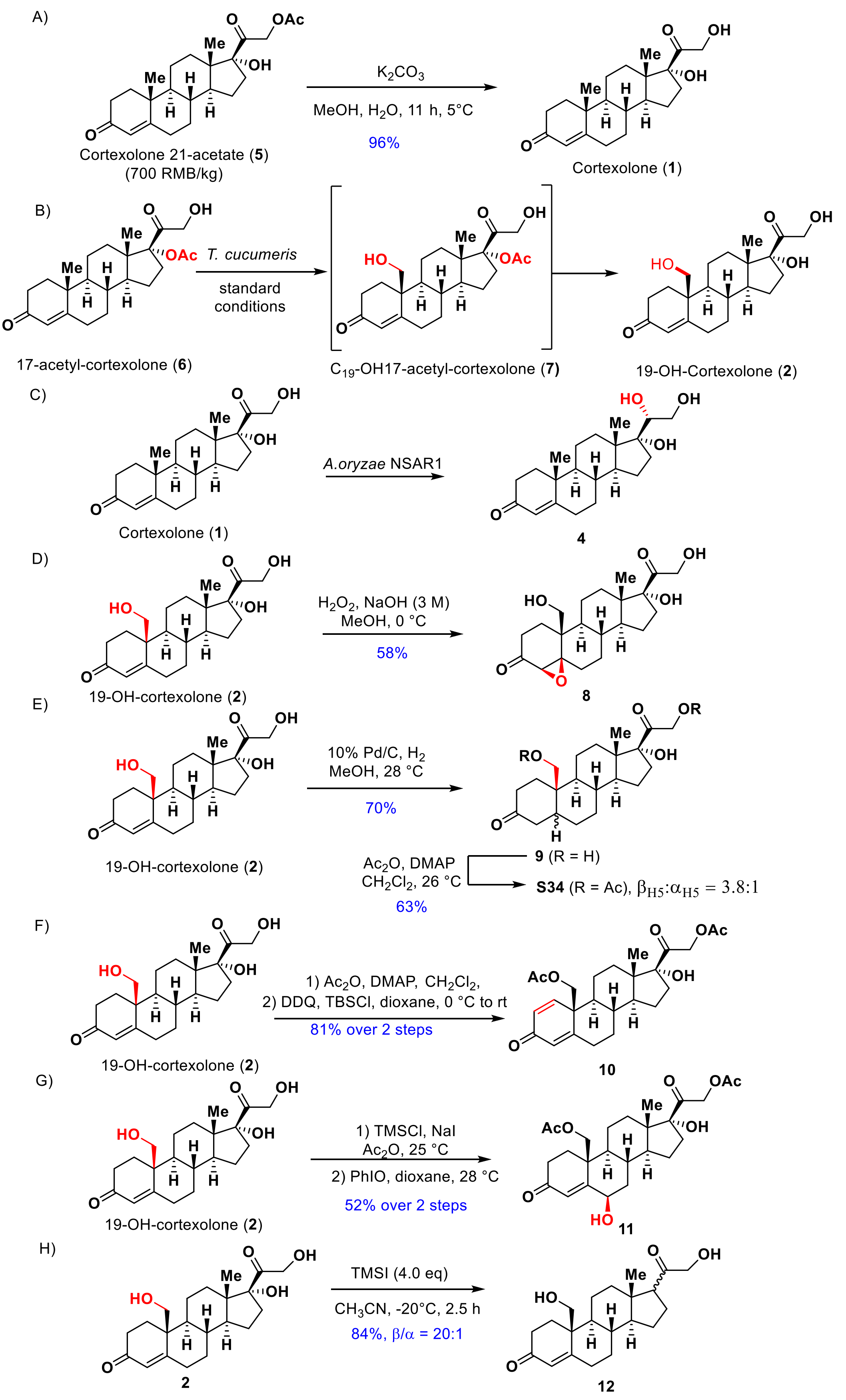

Supplementary Figure 79. Each synthetic step of **1**, **2**, **4** and **8-12.**

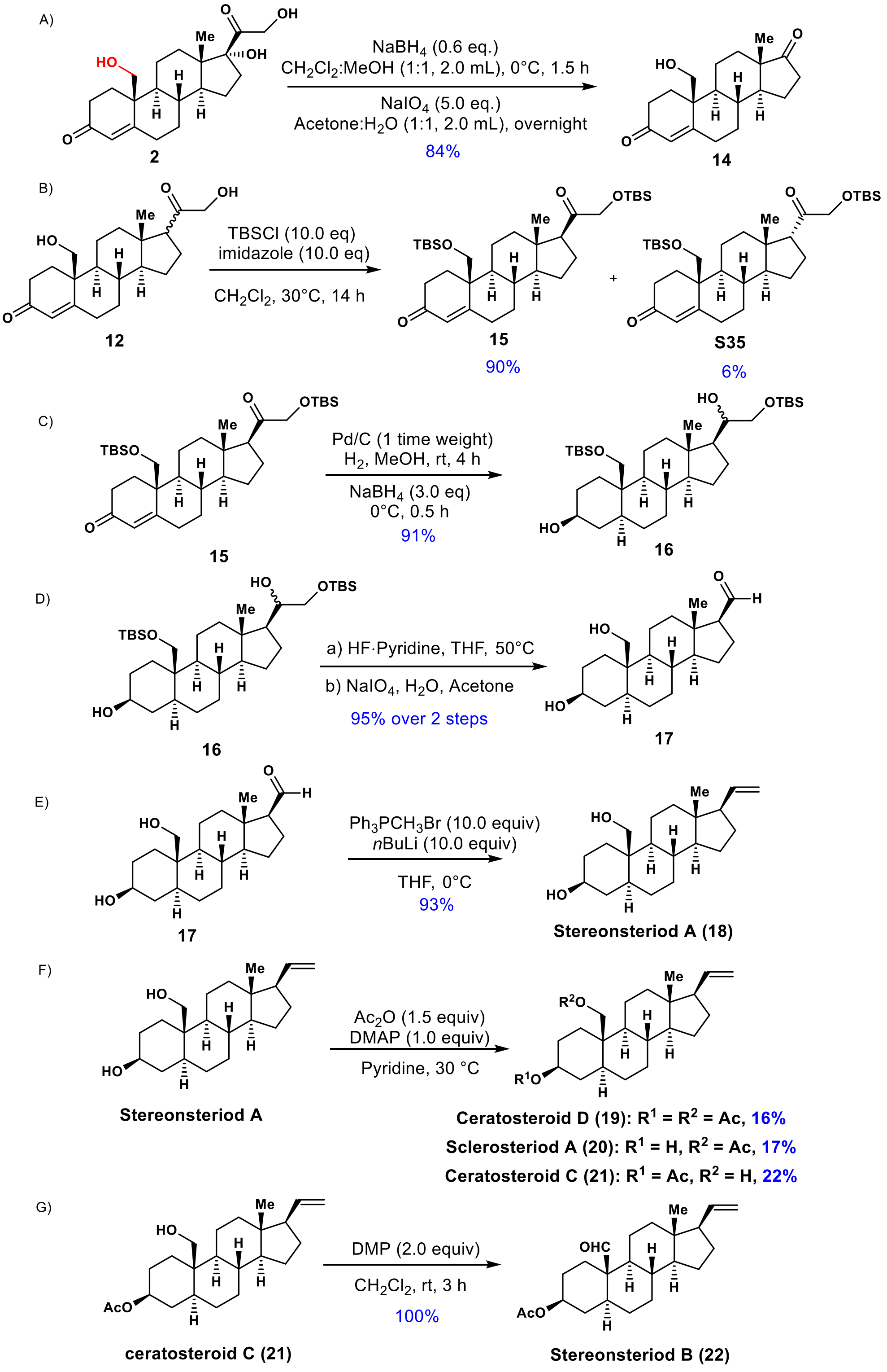

Supplementary Figure 80. Each synthetic step of **14-22.**

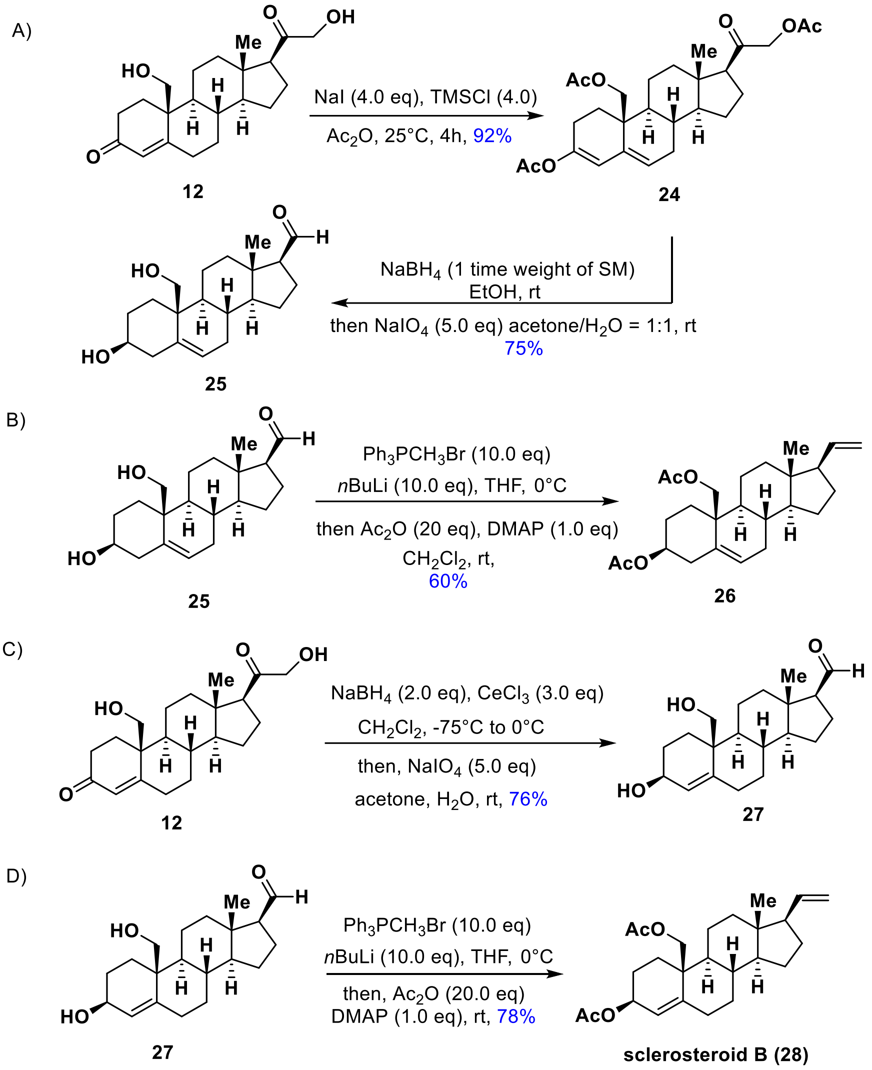

Supplementary Figure 81. Each synthetic step of **25-28.**

Supplementary Tables

Supplementary Table 1. Top 10 putative P450 genes in the *T. cucumeris* transcriptome ranked by FPKM values

	Gene	Acession No.	FPKM
1	*tcP450R*	MK309346	115.83
2	*tcP450-1*	MK309347	39.23
3	*tcP450-2*	MK309348	30.61
4	*tcP450-3*	MK309349	28.19
5	*tcP450-4*	MK309350	22.89
6	*tcP450-5*	MK309351	22.67
7	*tcP450-6*	MK309352	19.60
8	*tcP450-7*	MK309353	18.48
9	*tcP450-8*	MK309354	18.05
10	*tcP450-9*	MK309355	16.96

Supplementary Table 2. Primers used for construction of expression plasmids

primer	5' to 3' Sequence
tcP450-1-F	TAAACCCCACAGCAAGCTCCGAATTCGCCACCATGTCCAACTCAACTCTCGTTTCT
tcP450-1-R	GATCCCCGGGTACCGAGCTCGAATTCTTATTCCTCCACGAGTGTGACTTTC
Pamy-gt-F	TGGAGGATAGCAACCGACAAC
Tamy-gt-R	AAACTCACTGTCCAATGCCAGATT

Supplementary Table 3. Strains and plasmids

Strain/Plasmid	Characteristic(s)	Source/Reference
E. coli		
DH 5*α*	Host for general cloning	Invitrogen
Fungal		
T. cucumeris NBRC 6298	C19-OH cortexolone producing	NITE Biological Resource Center.
A. oryzae NSAR1	Heterologous expression strain	Prof. Ikuro. Abe (University of Tokyo)
mWHU2487	*Aspergillus oryzae* NSAR1 that has been transferred pWHU2490	This study
mWHU2488	*Aspergillus oryzae* NSAR1 that has been transferred pTAex3	This study
**Plasmids**		
pTAex3	Contains *argB* gene and both regions of the promoter and terminator of the *amyB* gene used for gene expression in *A. oryzae* NSAR1	Ikuro. Abe (university of Tokyo)
pWHU2490	pTAex3 derivative, for expression of tcP450-1 in *A. oryzae* NSAR1	This study

Supplementary Table 4. NMR data of Stereonsteriod A comparison between the synthetic sample and natural one[7,8]

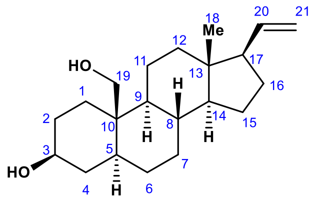

Stereonsteriod A

Position	Natural Stereonsteriod A (300 M, $CDCl_3$)	Synthetic Stereonsteriod A (400 M, $CDCl_3$)	Δ (Exp-Ref)/ppm
1	2.28 dt (13.5, 3.3)	2.27 dt (14.0, 3.9)	-0.01
3	3.65 m	3.66 tt (10.8, 4.9)	-0.01
17	1.97 m	1.95 m	-0.02
18	0.64 s	0.63 s	-0.01
19	3.81 d (11.4)	3.80 d (11.7)	-0.01
	3.95 d (11.4)	3.93 d (11.6)	-0.02
20	5.78 ddd (16.2, 10.5, 7.8)	5.76 ddd (17.6, 10.9, 7.7)	-0.02
21	4.97 d (16.2)	4.96 d (17.4)	-0.01
	4.98 d (10.5)	4.97 d (9.8)	-0.01

Position	Natural Stereonsteriod A (75 M, $CDCl_3$)	Synthetic Stereonsteriod A (101 M, $CDCl_3$)	Δ (Exp-Ref)/ppm
1	31.3	31.3	0
2	32.2	32.2	0
3	71.1	71.1	0
4	38.1	38.2	0.1
5	45.1	45.2	0.1
6	28.3	28.3	0
7	32.1	32.1	0
8	36.2	36.2	0
9	55.0	55.1	0.1
10	39.4	39.4	0
11	22.7	22.8	0.1
12	38.6	38.7	0.1
13	43.8	43.8	0
14	56.0	56.1	0.1
15	24.8	24.9	0.1
16	27.2	27.3	0.1
17	55.5	55.5	0
18	13.3	13.4	0.1
19	61.0	61.0	0
20	139.9	140.0	0.1
21	114.5	114.6	0.1

Supplementary Table 5. NMR data of Sclerosteriod A comparison between the synthetic sample and natural one[9]

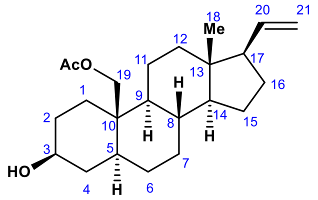

Sclerosteriod A

Position	Natural Sclerosteriod A (300 M, $CDCl_3$)	Synthetic Sclerosteriod A (600 M, $CDCl_3$)	Δ (Exp-Ref) (ppm)
1	2.21 dt (13.6, 3.6)	2.21 m	0
3	3.64 m	3.64 tt (10.6, 4.9)	0
18	0.57 s	0.57 s	0
19	4.21 d (12.0)	4.20 d (12.1)	-0.01
	4.35 d (12.0)	4.34 d (12.1)	-0.01
20	5.74 ddd (16.8, 10.8, 7.6)	5.74 ddd (16.4, 10.9, 7.7)	0
21	4.95 d (16.8)	4.95 m	0
	4.96 d (10.8)	4.96 m	0
OAc	2.06 s	2.06 s	0

Position	Natural Sclerosteriod A (75 M, $CDCl_3$)	Synthetic Sclerosteriod A (101 M, $CDCl_3$)	Δ (Exp-Ref) (ppm)
1	31.9	31.8	-0.1
2	31.6	31.6	0
3	70.8	70.9	0.1
4	38.4	38.4	0
5	45.1	45.0	-0.1
6	28.2	28.2	0
7	32.0	32.0	0
8	35.9	35.9	0
9	54.6	54.6	0
10	38.0	38.0	0
11	21.8	21.9	0.1
12	37.9	37.8	-0.1
13	43.6	43.6	0
14	55.9	55.9	0
15	24.7	24.7	0
16	27.2	27.2	0
17	55.3	55.3	0
18	13.0	13.0	0
19	62.9	62.9	0
20	139.8	139.8	0
21	114.5	114.5	0
OAc	171.2	171.3	0.1
	21.2	21.2	0

Supplementary Table 6. NMR data of Ceratosteriod C comparison between the synthetic sample and natural one[10]

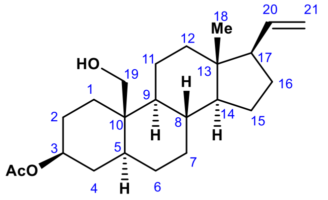

Ceratosteriod C

Position	Natural Ceratosteriod C (300 M, $CDCl_3$)	Synthetic Ceratosteriod C (600 M, $CDCl_3$)	Δ (Exp-Ref) (ppm)
1	2.32 dt (13.2, 3.3)	2.29 m	-0.03
3	4.73 m	4.74 tt (11.0, 5.0)	0.01
18	0.63 s	0.62 s	-0.01
19	3.82 d (11.4)	3.81 d (11.5)	-0.01
	3.96 d (11.4)	3.95 d (11.7)	-0.01
20	5.76 ddd (16.5, 11.1, 7.5)	5.75 ddd (16.3, 11.1, 7.7)	-0.01
21	4.96 d (16.5)	4.95 d (18.1)	-0.01
	4.97 d (11.1)	4.97 d (9.1)	0
OAc	2.00 s	2.02 s	0.02

Position	Natural Ceratosteriod C (75 M, $CDCl_3$)	Synthetic Ceratosteriod C (101 M, $CDCl_3$)	Δ (Exp-Ref) (ppm)
1	32.1	32.1	0
2	28.0	28.1	0.1
3	73.4	73.5	0.1
4	34.5	34.5	0
5	44.9	45.0	0.1
6	28.2	28.2	0
7	31.2	31.2	0
8	36.2	36.2	0
9	56.0	56.0	0
10	39.3	39.4	0.1
11	22.6	22.7	0.1
12	38.1	38.1	0
13	43.9	43.8	-0.1
14	54.9	54.9	0
15	24.8	24.9	0.1
16	27.2	27.3	0.1
17	55.4	55.5	0.1
18	13.2	13.3	0.1
19	60.9	61.0	0.1
20	139.9	140.0	0.1
21	114.6	114.7	0.1
OAc	171.1	170.9	-0.2
	21.5	21.6	0.1

Supplementary Table 7. NMR data of Ceratosteriod D comparison between the synthetic sample and natural one[10]

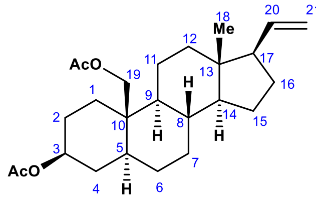

Ceratosteriod D

Position	Natural Ceratosteriod D (300 M, $CDCl_3$)	Synthetic Ceratosteriod D (400 M, $CDCl_3$)	Δ (Exp-Ref) (ppm)
1	2.23 dt (13.6, 3.4)	2.23 d (12.1)	0
3	4.73 m	4.72 tt (11.0, 4.9)	-0.01
18	0.57 s	0.57 s	0
19	4.24 d (12.0)	4.33 d (12.1)	-0.01
	4.34 d (12.0)	4.33 d (12.1)	-0.01
20	5.74 ddd (16.2, 10.8, 7.8)	5.74 ddd (16.3, 11.0, 7.8)	0
21	4.95 d (16.2)	4.98-4.93 m	0
	4.96 d (10.8)	4.98-4.93 m	0
OAc	2.02 s	2.02 s	0
	2.06 s	2.06 s	0

Position	Natural Ceratosteriod D (75 M, $CDCl_3$)	Synthetic Ceratosteriod D (101 M, $CDCl_3$)	Δ (Exp-Ref) (ppm)
1	32.0	32.1	0.1
2	27.6	27.7	0.1
3	73.1	73.2	0.1
4	34.3	34.3	0
5	44.9	45.0	0.1
6	28.2	28.2	0
7	31.7	31.7	0
8	36.0	36.0	0
9	54.6	54.6	0
10	38.1	38.1	0
11	21.9	21.9	0
12	37.9	37.9	0
13	43.7	43.8	0.1
14	55.9	56.0	0.1
15	24.8	24.9	0.1
16	27.2	27.3	0.1
17	55.4	55.5	0.1
18	13.1	13.2	0.1
19	62.8	62.9	0.1
20	139.8	139.9	0.1
21	114.6	114.7	0.1
OAc	170.7	170.9	0.2
	171.2	171.4	0.2
	21.2	21.4	0.2
	21.5	21.6	0.1

Supplementary Table 8. NMR data of Stereonsteriod B comparison between the synthetic sample and natural one[7]

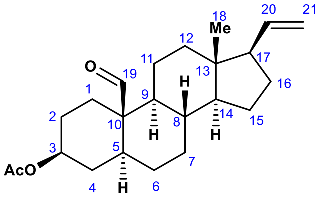

Stereonsteriod B

Position	Natural Stereonsteriod B (300 M, $CDCl_3$)	Synthetic Stereonsteriod B (600 M, $CDCl_3$)	Δ (Exp-Ref) (ppm)
1	2.43 dt (13.2, 3.3)	2.42 d (13.5)	-0.01
3	4.72 m	4.70 tt (10.5, 4.7)	-0.02
18	0.53 s	0.51 s	-0.02
19	10.03 s	10.02 s	-0.01
20	5.73 ddd (17.1, 10.5, 7.8)	5.72 ddd (17.5, 10.5, 7.9)	-0.01
21	4.96 d (17.1)	4.95 d (18.0)	-0.01
	4.97 d (10.5)	4.97 d (8.6)	0
OAc	2.00 s	2.00 s	0

Position	Natural Stereonsteriod B (75 M, $CDCl_3$)	Synthetic Stereonsteriod B (101 M, $CDCl_3$)	Δ (Exp-Ref) (ppm)
1	30.8	30.7	-0.1
2	28.5	28.4	-0.1
3	72.8	72.7	-0.1
4	35.6	35.6	0
5	43.4	43.3	-0.1
6	28.3	28.2	-0.1
7	32.0	31.9	-0.1
8	37.1	37.0	-0.1
9	52.8	52.7	-0.1
10	51.7	51.6	-0.1
11	21.4	21.4	0
12	37.4	37.2	-0.2
13	43.4	43.3	-0.1
14	55.8	55.7	-0.1
15	24.7	24.6	-0.1
16	27.1	27.1	0
17	55.3	55.3	0
18	12.8	12.8	0
19	208.3	208.3	0
20	139.5	139.5	0
21	114.8	114.7	-0.1
OAc	170.8	170.7	-0.1
	21.3	21.3	0

Supplementary Table 9. NMR data of Sclerosteriod B comparison between the synthetic sample and natural one[9]

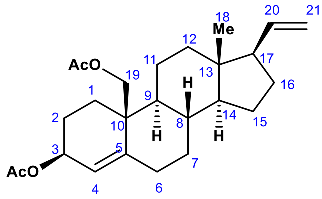

Sclerosteriod B

Position	Natural Sclerosteriod B (300 M, $CDCl_3$)	Synthetic Sclerosteriod B (600 M, $CDCl_3$)	Δ (Exp-Ref) (ppm)
1	2.03 m	2.03 m	0
3	5.22 m	5.21 m	-0.01
4	5.47 br s	5.46 s	-0.01
18	0.61 s	0.60 s	-0.01
19	4.14 d (11.2)	4.13 d (11.3)	-0.01
	4.49 d (11.2)	4.48 d (11.2)	-0.01
20	5.74 ddd (17.4, 10.4, 7.2)	5.74 ddd (16.7, 10.6, 7.7)	0
21	4.95 d (17.4)	4.96 m	0.01
	4.96 d (10.4)	4.97 m	0.01
OAc	2.05 s	2.05 s	0
	2.06 s	2.06 s	0

Position	Natural Sclerosteriod B (75 M, $CDCl_3$)	Synthetic Sclerosteriod B (101 M, $CDCl_3$)	Δ (Exp-Ref) (ppm)
1	31.5	31.7	0.2
2	25.4	25.5	0.1
3	70.0	70.1	0.1
4	122.2	122.4	0.2
5	145.0	145.1	0.1
6	36.4	36.5	0.1
7	32.3	32.5	0.2
8	33.1	33.2	0.1
9	54.4	54.6	0.2
10	40.5	40.7	0.2
11	21.1	21.2	0.1
12	37.6	37.8	0.2
13	43.5	43.6	0.1
14	55.6	55.8	0.2
15	24.6	24.8	0.2
16	27.1	27.3	0.2
17	55.2	55.3	0.1
18	12.9	13.1	0.2
19	66.9	67.0	0.1
20	139.6	139.8	0.2
21	114.7	114.9	0.2
OAc	171.0	171.1	0.1
	171.1	171.2	0.1
	21.2	21.3	0.1
	21.4	21.6	0.2

Supplementary Table 10. Crystal data and structure refinement for **23** (CCDC 1853184)

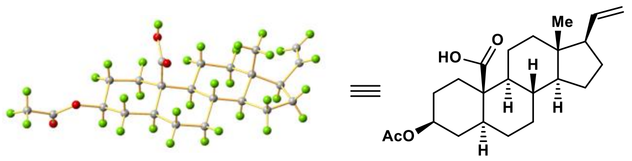

Identification code	CCDC 1853184	
Empirical formula	C23 H34 O4	
Formula weight	374.50	
Temperature	100(2) K	
Wavelength	0.71073 Å	
Crystal system	Triclinic	
Space group	P1	
Unit cell dimensions	a = 10.483(6) Å	a= 62.232(8)°.
	b = 11.031(6) Å	b= 86.296(9)°.
	c = 11.391(7) Å	g = 63.877(7)°.
Volume	1030.0(10) Å3	
Z	2	
Density (calculated)	1.208 Mg/m^3	
Absorption coefficient	0.081 mm^{-1}	
F(000)	408	
Crystal size	0.150 x 0.150 x 0.100 mm^3	
Theta range for data collection	2.198 to 25.499°.	
Index ranges	-12<=h<=12, -13<=k<=13, -12<=l<=13	
Reflections collected	7412	
Independent reflections	6011 [R(int) = 0.0478]	
Completeness to theta = 25.242°	98.5 %	
Absorption correction	None	
Refinement method	Full-matrix least-squares on F2	
Data / restraints / parameters	6011 / 45 / 513	
Goodness-of-fit on F^2	0.968	
Final R indices [I>2sigma(I)]	R1 = 0.1002, wR2 = 0.2674	
R indices (all data)	R1 = 0.1508, wR2 = 0.3217	
Absolute structure parameter	0.1(10)	
Extinction coefficient	n/a	
Largest diff. peak and hole	0.601 and -0.386 e.Å-3	

Supplementary Methods

Thanatephorus cucumeris NBRC 6298 was obtained from NITE Biological Resource Center. It is cultured either on PDA (200 g L^{-1} potato, 20 g L^{-1} dextrose and 15 g L^{-1} agar) or YD liquid medium (25 g L^{-1} dextrose and 20 g L^{-1} yeast extract)[11] for spores and mycelia growth, respectively. *Aspergillus oryzae* NSAR1 and corresponding plasmids pAdeA and pTAex3 were obtained from Prof. Ikuro Abe. *A. oryzae* is cultured either on DPY (dextrin-polypeptone-yeast extract: 2 % dextrin, 1 % polypeptone and 0.5 % yeast extract) medium or Czapek-Dox medium (218.6 g L^{-1} D-sorbitol, 3 g L^{-1} $NaNO_3$, 1 g L^{-1} KH_2PO_4, 0.5 g L^{-1} $MgSO_4{\cdot}7H_2O$, 0.5 g L^{-1} KCl, 0.01 g L^{-1} $FeSO_4{\cdot}7H_2O$, 30 g L^{-1} sucrose and 20 g L^{-1} Agar) supplemented with appropriate nutrients.[12-14] DNA isolation and manipulation in *Escherichia coli* were performed according to standard methods.[15] PCR amplifications were carried out on an authorized thermal cycler (Veriti 96 Well, ABI) using PrimeSTAR Max DNA polymerase (TaKaRa, Japan). TransScript RT/RI Enzyme Mix (TransGen, China) was used to generate cDNA through reverse transcription from mRNA. Primer synthesis and DNA sequencing were performed at Genewiz Biotech Co., Ltd. (China). Restriction enzymes were purchased from TaKaRa Biotechnology Co., Ltd. (China). Strains and plasmids used in this study are listed in Supplementary Table 3. HPLC analysis was carried out on an SHIMADZU LC-20A Prominence HPLC system. ORFs were identified using the Frame Plot 4.0 beta program (http://nocardia.nih.go.jp/fp4/). The corresponding deduced proteins were compared with other known proteins in the databases by using available BLAST methods (http://www.ncbi.nlm.nih.gov/blast/).

All reactions dealing with moisture-sensitive compound were performed by standard Schlenk techniques in oven-dried reaction vessels under Argon atmosphere. Unless otherwise noted, all solvents were dried by JC Meyer Solvent Drying System. Most reagents were purchased from commercial sources and used without further purification, unless otherwise stated. Reactions were monitored by thin layer chromatography (TLC) carried out on 0.2 mm commercial silica gel plates, using UV light as the visualizing agent or basic solution of $KMnO_4$ or Phosphomolybdic acid and heat as a developing agent. ^{1}H NMR spectra were recorded on Bruker 400 MHz or 600 MHz instrument and ^{13}C NMR spectra were recorded on Bruker 100 MHz instrument and were calibrated using residual undeuterated solvent as an internal reference ($CDCl_3$ @ 7.26 ppm ^{1}H NMR, 77.16 ppm ^{13}C NMR). The following abbreviations were used to explain multiplicities: s= singlet, d= doublet, t= triplet, q= quartet, dd= doublet of doublets, dt= doublet of triplets, td= triplet of doublets, ddd= doublet of doublet of doublets, m= multiplet, br= broad. High resolution mass spectra (HRMS) were recorded on DIONEX UltiMate 3000 & Bruker Compact TOF mass spectrometer.

Supplementary Notes

Supplementary Note 1: Cortexolone (**1**, Supplementary Figure 79A) synthesis and characterization.

To a suspension of **RSA** (5.0 g, 12.9 mmol) in MeOH (60 mL) and H_2O (40 mL) was added K_2CO_3 (3.56 g, 25.7mmol, 2.0 equiv) at 5 °C. After stirring for 11 h, MeOH was removed *in vaccuo*, then the mixture was filtered and washed with H_2O (50 mL). The solid was dried to afford Cortexolone **1** (4.27 g, 96%).

Physical state: white solid;

R_f = 0.7 (silica gel, 1:1 CH_2Cl_2: EtOAc);

^{1}H NMR (400 MHz, $CDCl_3$) δ 5.75–5.72 (m, 1H), 4.67 (dd, J = 19.9, 4.9 Hz, 1H), 4.31 (dd, J = 19.9, 4.8 Hz, 1H), 3.10 (t, J = 4.9 Hz, 1H), 2.69 (ddd, J = 14.8, 11.5, 3.1 Hz, 1H), 2.46–2.25 (m, 4H), 2.03 (ddd, J = 13.4, 5.0, 3.2 Hz, 1H), 1.85 (dddd, J = 16.4, 12.2, 7.5, 3.0 Hz, 2H), 1.78–1.56 (m, 7H), 1.47–1.33 (m, 3H), 1.18 (s, 3H), 1.16–1.04 (m, 1H), 0.97 (ddd, J = 11.9, 10.4, 4.1 Hz, 1H), 0.71 (s, 3H).

Supplementary Note 2: 19-OH-cortexolone **(2**, Supplementary Figure 79B**)** and 19-OH-17-acetyl-cortexolone (**7**) synthesis and characterization.

T. cucumeris was inoculated into the liquid medium and cultured at 30 °C and 200 rpm for 2 d. After that, 4 mL seed culture was transferred to a 250 mL Erlenmeyer flask containing 100 mL of YD liquid medium and continued to incubation under the same culture conditions for 2 d. 17-acetyl-cortexolone **6** (25 mg) was treated with fresh cultured *T. cucumeris* liquid medium (0.25 g L^{-1}). After bioconvertion for 24 h, all of the substrate **6** has been converted into **2** and **7** (**2**:**7** = 0.18: 1). After three days, compound **7** almost converted into **2** (20 mg, 80% yield). Following this way, we further scaled up the biotransformation. Substrates **6** (7.5 g) and $FeSO_4$ were evenly added into 30 3 L-Erlenmeyer flasks each containing 1 L of *T. cucumeris* culture on the 3rd day (the final concentration for **6** and $FeSO_4$ are 0.25 g L^{-1} and 1.5 mM respectively). The cultures were then fermented under the same conditions for 3 d. After fermentation, the culture was added with ethyl acetate and extracted under sonication for 15 min for three times. Organic phase was combined and dried *in vacuo*. the resulting residue was purified by silica gel column chromatography (1:1 CH_2Cl_2: EtOAc) to afford the desired reduction product **2** (5.6 g, 80.3%). (The ^{1}H NMR and ^{13}C NMR matched the literature reported data[1])

For **7**:

Physical state: white solid;

R_f = 0.25 (silica gel, 1:1 CH_2Cl_2: EtOAc);

HRMS (*m*/*z*): calcd for $C_{23}H_{33}O_6$, $[M+H]^+$ 405.2277; found, 405.2274;

^{1}H NMR (400 MHz, $CDCl_3$): δ 5.94 (s, 1H), 4.26 (d, J = 2.7 Hz, 2H), 4.04 (dd, J = 10.6, 1.2 Hz, 1H), 3.90 (d, J = 10.7 Hz, 1H), 2.90–2.66 (m, 2H), 2.46–2.33 (m, 3H), 2.08 (s, 3H), 1.92–1.64 (m, 8H), 1.57–1.42 (m, 2H), 1.41–1.33 (m, 1H), 1.27–1.03 (m, 3H), 0.94–0.89 (m, 1H), 0.67 (s, 3H).

^{13}C NMR (101 MHz, $CDCl_3$): δ 206.3, 200.2, 171.0, 166.7, 127.0, 94.0, 67.1, 66.0, 53.4, 51.3, 47.8, 43.80, 36.3, 35.1, 33.6, 33.5, 32.1, 30.9, 30.8, 23.9, 21.3, 21.1, 14.5.

For **2**:

Physical state: white solid;

R_f = 0.2 (silica gel, 1:1 petroleum ether: EtOAc);
$[\alpha]_D^{25}$ = +132 (*c* 0.33, EtOH)
HRMS (*m*/*z*): calcd for $C_{21}H_{30}O_5Na$, $[M+Na]^+$ 385.1985; found, 385.1995;
^{1}H NMR (400 MHz, CD_3OD) *δ* 5.88 (d, *J* = 1.6 Hz, 1H), 4.62 (d, *J* = 19.3 Hz, 1H), 4.28 (d, *J* = 19.3 Hz, 1H), 4.02 (dd, *J* = 11.1, 1.2 Hz, 1H), 3.85 (d, *J* = 11.1 Hz, 1H), 2.82–2.63 (m, 2H), 2.55 (tdd, *J* = 14.1, 5.2, 1.9 Hz, 1H), 2.45–2.23 (m, 3H), 1.99–1.91 (m, 1H), 1.86–1.71 (m, 5H), 1.59–1.33 (m, 5H), 1.17–1.02 (m, 2H), 0.67 (s, 3H).
^{13}C NMR (101 MHz, CD_3OD) *δ* 213.3, 203.2, 171.7, 126.7, 90.3, 67.8, 66.0, 55.3, 52.3, 45.3, 37.5, 35.8, 35.0, 34.7, 34.5, 33.8, 32.1, 30.8, 24.5, 22.3, 15.4.

Supplementary Note 3: C_{20}-OH-cortexolone (**4**, Supplementary Figure 79C) synthesis and characterization.
Following the Cortexolone bioconversion by *A.oryzae* NSAR1 procedure, cortexolone **1** (25 mg) was treated with fresh cultured *A. oryzae* NSAR1 liquid medium (0.25 g L^{-1}). After work-up and HPLC purification, C_{20}-OH-cortexolone **4** (10.6 mg, 42% yield) was obtained as a white solid.
Physical state: yellow solid;
R_f = 0.4 (silica gel, 1:2 CH_2Cl_2: EtOAc);
$[\alpha]_D^{25}$ = +45.0 (*c* 0.1, $CHCl_3$/MeOH 1:1)
HRMS (*m*/*z*): $C_{21}H_{33}O_4$, $[M+H]^+$ 349.2379; found, 349.2385;
^{1}H NMR (400 MHz, MeOD) *δ* 5.68 (s, 1H), 3.74 (dd, *J* = 7.3, 3.2 Hz, 1H), 3.66–3.55 (m, 2H), 2.53–2.39 (m, 2H), 2.33–2.22 (m, 2H), 2.06 (ddd, *J* = 13.4, 5.1, 3.1 Hz, 1H), 1.91–1.75 (m, 3H), 1.73–1.54 (m, 6H), 1.52–1.40 (m, 2H), 1.31–1.24 (m, 1H), 1.21 (s, 3H), 1.10–0.98 (m, 1H), 0.96–0.89 (m, 1H), 0.86 (s, 3H).
^{13}C NMR (101 MHz, MeOD) *δ* 202.5, 175.5, 124.1, 86.0, 76.6, 65.2, 55.3, 50.5, 40.0, 37.0, 36.8, 34.7, 34.5, 34.0, 33.5, 33.1, 24.9, 21.8, 17.7, 15.2.

Supplementary Note 4: Compound **8** (Supplementary Figure 79D) synthesis and characterization.
To a solution of 19-OH-cortexolone **2** (10 mg, 30 μmol, 1.0 equiv) in MeOH (1.0 mL) was added aq. NaOH (3.0 M, 10 μL, 0.03 mmol, 1.0 equiv) and H_2O_2 (30% in H_2O, 20 μL, 0.15 mmol, 5.0 equiv) at 0 °C. After stirring for 4 h. The reaction mixture was diluted with CH_2Cl_2 (5 mL), washed with H_2O (5 mL) and brine (5 mL). The combined extracts were dried over anhydrous Na_2SO_4, filtered and concentrated. The crude product was purified by silica gel column chromatography (1:1 CH_2Cl_2: EtOAc) to afford epoxide **8** (6.6 mg, 17.4 μmol, 58%) as a white solid.
Physical state: white solid;
R_f = 0.3 (silica gel, 1:2 CH_2Cl_2: EtOAc);
$[\alpha]_D^{25}$ = +40.1 (*c* 0.46, CH_3OH)
HRMS (*m*/*z*): calcd for $C_{21}H_{30}O_6Na$, $[M+Na]^+$ 401.1935; found, 401.1944;
^{1}H NMR (400 MHz, Methanol-*d*$_4$) *δ* 4.72–4.52 (m, 2H), 4.28 (d, *J* = 19.2 Hz, 1H), 4.02 (d, *J* = 11.3 Hz,

1H), 3.76 (d, J = 11.4 Hz, 1H), 2.68 (ddd, J = 14.3, 11.4, 2.7 Hz, 1H), 2.36–2.21 (m, 2H), 2.20–2.10 (m, 1H), 1.96–1.90 (m, 1H), 1.86–1.73 (m, 3H), 1.68–1.60 (m, 2H), 1.56–1.47 (m, 2H), 1.44–1.37 m, 1H), 1.37–1.30 (m, 4H), 1.23–1.09 (m, 3H), 0.66 (s, 3H).

^{13}C NMR (101 MHz, MeOD) δ 211.9, 207.1, 89.0, 68.8, 66.4, 63.5, 59.7, 50.8, 46.1, 41.4, 35.0, 33.5, 31.6, 30.5, 30.5, 29.7, 23.2, 21.7, 20.7, 14.1.

Supplementary Note 5: Compound **S34** (Supplementary Figure 79E) synthesis and characterization.
To a solution of 19-OH-cortexolone **2** (10.0 mg, 30.0 μmol, 1.0 equiv) in MeOH (1.5 mL) was added 10% Pd/C (5.0 mg) at 28 °C under H_2 atmosphere. After stirring for 1 h. The mixture was filtered over a pad of Celite@ and concentrated *in vacuo*. Since the reduction selectivity could not be confirmed by ^{1}H NMR, further derivatization was required. The reductive product **9** (7.0mg, 19.2 μmol, 70%) was dissolved in CH_2Cl_2 (2.0 mL). Then, Ac_2O (10.0 mg, 5.0 equiv) and DMAP (3.0 mg, 0.02 mmol, 1.0 equiv) was added. The solution was stirred under argon for 12 h. Then, the reaction mixture was diluted with CH_2Cl_2 (5 mL), washed with saturated $NaHCO_3$ (3 x 5 mL) and brine (5 mL). The combined extracts were dried over anhydrous Na_2SO_4, filtered and concentrated. the crude product was purified by silica gel column chromatography (2:1 petroleum ether: EtOAc) to afford **S34** (7.7 mg, 63%) as a white solid.
Physical state: white solid;
R_f = 0.6 (silica gel, 1:1 petroleum ether: EtOAc);
$[\alpha]_D^{25}$ = +35.5 (c 0.48, $CHCl_3$)
HRMS (m/z): calcd for $C_{25}H_{36}O_7Na$, $[M+Na]^+$ 471.2353; found, 471.2358;
^{1}H NMR (400 MHz, $CDCl_3$) δ 5.08 (dd, J = 17.5, 12.9 Hz, 1H), 4.83 (d, J = 17.4 Hz, 0.19H_α), 4.80 (d, J = 17.4 Hz, 0.64H_β), 4.46–4.37 (m, 1H), 4.08 (d, J = 11.3 Hz, 1H).
^{13}C NMR (101 MHz, $CDCl_3$) δ 212.2, 205.1, 171.2, 170.7, 90.0, 67.8, 66.3, 51.4, 48.5, 42.0, 40.4, 37.9, 36.9, 36.6, 35.5, 34.9, 31.0, 30.7, 29.7, 29.3, 25.9, 25.5, 23.5, 21.0, 20.9, 20.6, 14.8.

Supplementary Note 6: Compound **10** (Supplementary Figure 79F) synthesis and characterization.
To a solution of 19-OH-cortexolone **2** (6.0 mg, 16.6 μmol, 1.0 equiv) in CH_2Cl_2 (1.0 mL) was added Ac_2O (7.8 μL, 0.08 mmol, 5.0 equiv) and DMAP (2.0 mg, 0.017 mmol, 1.0 equiv) at 29 °C. After stirring for 4 h. Reaction mixture was diluted with CH_2Cl_2 (5 mL), washed with saturated $NaHCO_3$ (3 x 5 mL) and brine (5 mL). The organic phase was dried over anhydrous Na_2SO_4, filtered and concentrated. Without further purification, crude product was dissolved in dioxane (1.0 mL). Then, TBSCl (0.6 mg, 4.0 μmol, 0.1 equiv) and DDQ (12.0 mg, 0.052 mmol, 1.3 equiv) were added to mixture at 0 °C under argon atmosphere. The mixture was reflux for 5 h, then diluted with CH_2Cl_2 (5 mL), washed with saturated $NaHCO_3$ (3 x 5 mL) and brine (5 mL). The combined extracts were dried over anhydrous Na_2SO_4, filtered and concentrated. the crude product was purified by silica gel column chromatography (3:1 petroleum ether: EtOAc) to afford **10** (6.5 mg, 14.6 μmol, 81%) as a colorless oil.
Physical state: colorless oil;

R_f = 0.5 (silica gel, 1:1 petroleum ether: EtOAc);

$[\alpha]_D^{25}$ = +23.7 (*c* 0.18, $CHCl_3$)

HRMS (*m*/*z*): calcd for $C_{25}H_{34}O_8Na$, $[M+Na]^+$ 485.2146; found, 485.2145;

^{1}H NMR (400 MHz, $CDCl_3$) δ 7.08 (d, *J* = 10.2 Hz, 1H), 6.35 (dd, *J* = 10.2, 1.9 Hz, 1H), 6.18 (d, *J* = 1.6 Hz, 1H), 5.06 (d, *J* = 17.5 Hz, 1H), 4.81 (d, *J* = 17.5 Hz, 1H), 4.62 (d, *J* = 11.0 Hz, 1H), 4.39 (d, *J* = 10.9 Hz, 1H), 2.77–2.68 (m, 1H), 2.56–2.48 (m, 1H), 2.45–2.38 (m, 1H), 2.16 (s, 3H), 2.07–1.98 (m, 1H), 1.97–1.92 (m, 1H), 1.91 (s, 3H), 1.83–1.62 (m, 4H), 1.55–1.46 (m, 1H), 1.44–1.34 (m, 1H), 1.30–1.09 (m, 4H), 0.73 (s, 3H).

^{13}C NMR (101 MHz, $CDCl_3$) δ 205.2, 186.4, 170.9, 170.8, 164.5, 151.9, 130.4, 126.6, 89.8, 68.0, 63.7, 52.6, 50.6, 48.5, 47.9, 36.2, 34.9, 33.9, 32.8, 30.4, 23.8, 23.1, 20.8, 20.7, 14.8.

Supplementary Note 7: Compound **11** (Supplementary Figure 79G) synthesis and characterization.
Argon was bubbled for 20 min through stirred Ac_2O (1.0 mL) containing 19-OH-cortexolone **2** (5.0 mg, 14.5 μmol, 1.0 equiv) and NaI (8.7 mg, 60 μmol, 4.0 equiv). The mixture was cooled to 0 °C, and TMSCl (7.6 μL, 60 μmol, 4.0 equiv) was added dropwise, then, the mixture was allowed to warm to 25 °C and stirring for 4 h under an argon atmosphere. The reaction mixture was then poured into saturated aqueous $NaHCO_3$ (10 mL). After the mixture was stirred for 10 min, the product was extracted using EtOAc (3 × 15 mL). The combined extracts were washed with 10% aqueous $Na_2S_2O_3$ (2 × 10 mL), and brine (5 mL) and dried over anhydrous Na_2SO_4. The solvent was removed to afford acetylized product which was used without further purification or characterization. Then, the acetylized product was dissolved in dioxane (1.0 mL), and PhIO (5.1 mg, 25 μmol, 1.7 equiv) was added. The solution was stirred under argon for 7 h. Then, the mixture was diluted with EtOAc (10 mL) and washed with H_2O (10 mL) and brine (10 mL). The combined extracts were dried over anhydrous Na_2SO_4, filtered and concentrated. The crude product was purified by silica gel column chromatography (2:1 petroleum ether: EtOAc) to afford **11** (3.5 mg, 7.5 μmol, 52% for two steps) as a white solid.

Physical state: white solid;

R_f = 0.4 (silica gel, 1:1 petroleum ether: EtOAc);

$[\alpha]_D^{25}$ = +59.4 (*c* 0.51, $CHCl_3$)

HRMS (*m*/*z*): calcd for, $C_{25}H_{30}O_7Na$, $[M+Na]^+$ 467.2040; found, 467.2045;

^{1}H NMR (400 MHz, $CDCl_3$) δ 6.00 (s, 1H), 5.08 (d, *J* = 17.5 Hz, 1H), 4.81 (d, *J* = 17.4 Hz, 1H), 4.75 (dd, *J* = 11.1, 1.4 Hz, 1H), 4.41–4.35 (m, 2H), 2.79–2.70 (m, 1H), 2.68–2.57 (m, 1H), 2.45–2.32 (m, 3H), 2.17 (s, 3H), 2.14–2.04 (m, 2H), 2.02 (s, 3H), 1.87–1.71 (m, 5H), 1.58–1.37 (m, 4H), 1.15–1.07 (m, 1H), 0.75 (s, 3H).

^{13}C NMR (101 MHz, $CDCl_3$) δ 205.0, 199.9, 170.9, 170.7, 162.0, 128.9, 89.8, 72.7, 68.4, 67.8, 53.6, 50.7, 48.3, 41.1, 38.7, 34.79, 34.76, 34.5, 30.4, 30.3, 29.7, 23.5, 21.2, 21.1, 20.6, 14.7

Supplementary Note 8: Compound **12** (Supplementary Figure 79H) synthesis and characterization.

To a solution of **2** (100 mg, 0.28 mmol) in anhydrous acetonitrile (70 mL) was dropwise added TMSI (221 mg, 1.1 mmol, 4.0 equiv) at -20 °C under argon atmosphere. After stirring for 2.5 h, the reaction mixture was quenched with saturated $Na_2S_2O_3$ solution. Most of acetonitrile was removed under reduced pressure, then, the mixture was diluted with CH_2Cl_2 (50 mL), washed with saturated $NaHCO_3$ (2 x 25 mL) and brine (15 mL). The combined extracts were dried over anhydrous Na_2SO_4, filtered and concentrated *in vacuo*. The crude product was purified by silica gel chromatography (40:1 CH_2Cl_2: MeOH) to give **12** (80 mg, 84%, β/α = 20:1) as a white solid. (The ^{1}H NMR and ^{13}C NMR matched the literature reported data[5])

Physical state: white solid;

R_f = 0.4 (silica gel, 15:1 CH_2Cl_2: MeOH);

$[\alpha]_D^{25}$ = +156 (*c* 0.33, $CHCl_3$)

HRMS (*m/z*): calcd for $C_{21}H_{30}O_4Na$, $[M+Na]^+$ 369.2036; found, 369.2039;

^{1}H NMR (600 MHz, $CDCl_3$): *δ* 5.99 (s, 1H), 4.24 (d, *J* = 19.2 Hz, 1H), 4.20 (d, *J* = 19.0 Hz, 1H), 4.09 (d, *J* = 10.7 Hz, 1H), 3.94 (d, *J* = 10.8 Hz, 1H), 2.83–2.69 (m, 1H), 2.52–2.35 (m, 5H), 2.29–2.24 (m, 1H), 2.01–1.93 (m, 2H), 1.85–1.76 (m, 4H), 1.73–1.68 (m, 2H), 1.56–1.49 (m, 1H), 1.45–1.28 (m, 2H), 1.22–1.11 (m, 2H), 0.99 (s, α-0.14H), 0.73 (s, β-3H).

^{13}C NMR (101 MHz, $CDCl_3$): *δ* 210.2, 200.4, 167.0, 126.9, 69.5, 65.9, 59.0, 56.5, 53.8, 44.8, 43.9, 38.8, 36.3, 35.1, 33.6, 33.5, 32.1, 24.5, 23.0, 21.5, 13.7.

Supplementary Note 9: Compound **14** (Supplementary Figure 80A) synthesis and characterization.

To a solution of **2** (10 mg, 0.03 mmol) in 1:1 CH_2Cl_2:EtOH (2.0 mL) at 0 ºC was added $NaBH_4$ (0.7 mg, 0.6 equiv). After 1.5 h, A mixture of acetone ans water (1:1, 2.0 mL) were added, the mixture was warmed to ambient temperature, followed by addition of $NaIO_4$ (32 mg, 5 equiv). The resulting white slurry was stirred overnight, filtered through Celite® and evaporated until the majority of CH_2Cl_2, EtOH and acetone had been removed to provide crude product. The crude product was purified by silica gel chromatography (2:1 petroleum ether: EtOAc) to give **14** (7.0 mg, 84%).

Physical state: white solid;

R_f = 0.35 (silica gel, 2:1 petroleum ether: EtOAc);

^{1}H NMR (400 MHz, $CDCl_3$): *δ* 5.96 (s, 1H), 4.07 (dd, *J* = 10.7, 1.3 Hz, 1H), 3.94 (d, *J* = 10.7 Hz, 1H), 2.72 (ddd, *J* = 16.9, 14.1, 5.9 Hz, 1H), 2.52–2.32 (m, 5H), 2.16–1.94 (m, 3H), 1.91–1.74 (m, 4H), 1.64–1.44 (m, 3H), 1.23–1.05 (m, 3H), 0.91 (s, 3H).

^{13}C NMR (101 MHz, $CDCl_3$): *δ* 220.4, 199.9, 166.2, 127.3, 66.1, 54.1, 51.3, 47.7, 43.9, 36.0, 35.8, 35.1, 33.6, 33.5, 31.8, 31.0, 21.8, 21.0, 14.0.

Supplementary Note 10: Compound **15** (Supplementary Figure 80B) **and S33** synthesis and characterization.

To a solution of **12** (80 mg, 0.23mmol, 1.0 equiv) in anhydrous CH_2Cl_2 (5 mL) were added imidazole (156

mg, 2.3 mmol, 10.0 equiv) and TBSCl (347 mg, 2.3 mmol, 10.0 equiv) at 30 °C under argon atmosphere. After stirring for 14 h, the mixture was diluted with CH_2Cl_2 (15 mL), washed with saturated $NaHCO_3$ (2 x 15 mL), 1 M HCl (2 x 15 mL) and brine (15 mL). The combined extracts were dried over anhydrous Na_2SO_4, filtered and concentrated *in vacuo*. The crude product was purified by silica gel chromatography (15:1 petroleum ether: EtOAc) to give **15** (120 mg, 90%) as a colorless oil and **S35** (8.5 mg, 6%) as a colorless oil.

For **15**:

Physical state: colorless oil;

R_f = 0.5 (silica gel, 5:1 petroleum ether: EtOAc);

$[\alpha]_D^{25}$ = +109.1 (c = 1.0, $CHCl_3$);

HRMS (m/z): calcd for $C_{33}H_{58}O_4Si_2Na$, $[M+Na]^+$ 597.3766; found, 597.3750;

^1H NMR (400 MHz, $CDCl_3$): δ 5.85 (s, 1H), 4.21 (d, J = 17.8 Hz, 1H), 4.13 (d, J = 17.8 Hz, 1H), 3.87 (d, J = 9.9 Hz, 1H), 3.83 (d, J = 9.8 Hz, 1H), 2.70–2.56 (m, 2H), 2.43–2.25 (m, 4H), 2.21–2.10 (m, 1H), 1.92–1.83 (m, 2H), 1.74–1.62 (m, 6H), 1.52 (qd, J = 13.1, 3.8 Hz, 1H), 1.35–1.23 (m, 2H), 1.16–0.97 (m, 3H), 0.90 (s, 9H), 0.82 (s, 9H), 0.67 (s, 3H), 0.09–0.05 (m, 6H), 0.02 (s, 3H), 0.01 (s, 3H).

^{13}C NMR (101 MHz, $CDCl_3$): δ 209.9, 200.3, 167.9, 126.0, 70.7, 65.8, 57.7, 56.7, 53.9, 44.7, 43.7, 39.1, 36.4, 34.9, 33.7, 33.5, 32.1, 26.0, 25.8, 24.6, 23.2, 21.7, 18.6, 18.1, 13.9, -5.2, -5.6, -5.7.

For **S33**:

Physical state: colorless oil;

R_f = 0.53 (silica gel, 5:1 petroleum ether: EtOAc);

$[\alpha]_D^{25}$ = +45.7 (c = 1.9, $CHCl_3$);

HRMS (m/z): calcd for $C_{33}H_{58}O_4Si_2Na$, $[M+Na]^+$ 597.3766; found, 597.3738;

^1H NMR (400 MHz, $CDCl_3$): δ 5.83 (s, 1H), 4.15 (d, J = 17.9 Hz, 1H), 4.05 (d, J = 17.9 Hz, 1H), 3.86 (d, J = 9.9 Hz, 1H), 3.82 (d, J = 9.8 Hz, 1H), 3.05 (dd, J = 8.3, 2.4 Hz, 1H), 2.63–2.54 (m, 1H), 2.39–2.22 (m, 4H), 1.91–1.47 (m, 9H), 1.32–1.27 (m, 2H), 1.17–1.02 (m, 2H), 1.01–0.95 (m, 1H), 0.94 (s, 3H), 0.91 (s, 9H), 0.83 (s, 9H), 0.08 (s, 3H), 0.06 (s, 3H), 0.02 (s, 3H), 0.01 (s, 3H).

^{13}C NMR (101 MHz, $CDCl_3$): δ 214.1, 200.1, 167.9, 125.9, 70.6, 65.7, 54.5, 53.2, 50.6, 46.0, 43.5, 36.5, 34.8, 34.8, 33.7, 33.3, 32.2, 25.88, 25.86, 25.7, 24.3, 21.6, 20.5, 18.4, 18.0, -5.3, -5.5, -5.7, -5.8.

Supplementary Note 11: Compound **16** (Supplementary Figure 80C) synthesis and characterization.

To a solution of **15** (5.5 mg, 9.6 μmol, 1.0 equiv) in MeOH (1.0 mL) was added 10% Pd/C (5.5 mg, one-time weight of starting material) at room temperature under H_2 atmosphere. After stirring for 4 h, $NaBH_4$ (1.9 mg, 0.05 mmol, 5.2 equiv) was added at 0 °C. When the starting material was completely consumed, the mixture was filtered through a pad of Celite@ and concentrated, the resulting residue was purified by silica gel column chromatography (8:1 petroleum ether: EtOAc) to afford the desired reduction product **16** (5.0 mg, 8.7 μmol, 91%) as a white solid.

Physical state: white solid;

R_f = 0.4 (silica gel, 5:1 petroleum ether: EtOAc);
$[\alpha]_D^{25}$ = +2.6 (c =0.6, $CHCl_3$);
HRMS (m/z): calcd for $C_{33}H_{64}O_4Si_2Na$, $[M+Na]^+$ 603.4235; found, 603.4234;
^{1}H NMR (400 MHz, $CDCl_3$): δ 3.77 (d, J = 10.5 Hz, 1H), 3.63–3.46 (m, 4H), 3.25 (dd, J = 9.5, 7.2 Hz, 1H), 2.39 (s, 1H), 2.23 (dt, J = 13.5, 3.7 Hz, 1H), 2.05 (dt, J = 12.7, 3.4 Hz, 1H), 1.73 (d, J = 10.4 Hz, 1H), 1.64–1.43 (m, 11H), 1.37–1.25 (m, 4H), 1.18–0.93 (m, 8H), 0.83 (s, 9H), 0.82 (s, 9H), 0.71 (s, 3H), 0.63 (ddd, J = 19.7, 11.4, 3.8 Hz, 2H), 0.00 (d, J = 1.0 Hz, 10H), -0.01 (s, 3H).
^{13}C NMR (101 MHz, $CDCl_3$): δ 74.3, 71.5, 66.9, 60.5, 56.2, 55.1, 52.3, 45.0, 43.1, 40.4, 39.3, 38.8, 36.0, 32.4, 32.0, 31.8, 28.5, 27.1, 26.02, 25.99, 24.9, 24.5, 22.7, 18.4, 18.2, 12.8, -5.2, -5.3, -5.5, -5.6.

Supplementary Note 12: Compound **17** (Supplementary Figure 80D) synthesis and characterization.
To a solution of **16** (82 mg, 0.14 mmol, 1.0 equiv) in anhydrous THF (5.0 mL) was added HF·Pyridine (1 M, 0.5 mL) at 50 °C under argon atmosphere. After stirring for 4.5 h, the mixture was poured into saturated $NaHCO_3$ (20 mL) and extracted with CH_2Cl_2 (3 x 15 mL). Then, the organic phase was washed with 1 M HCl (3 x 15 mL), dried over anhydrous Na_2SO_4, filtered and concentrated. Without purification, the desilyl product was dissolved in acetone (3 mL) and H_2O (3 mL). Then, $NaIO_4$ (150 mg, 0.7 mmol, 5.0 equiv) was added to the mixture at rt. After stirring for 3 h, the mixture was diluted with CH_2Cl_2 (15 mL), washed with H_2O (3 x 15 mL) and brine (15 mL). The combined extracts were dried over anhydrous Na_2SO_4, filtered and concentrated. The crude product was purified by silica gel column chromatography (20:1 CH_2Cl_2: MeOH) to afford the desired aldehyde **17** (43 mg, 0.13 mmol, 95% for two steps) as a white solide.
Physical state: white solid;
R_f = 0.5 (silica gel, 6:1 CH_2Cl_2: MeOH);
$[\alpha]_D^{25}$ = +46.3 (c =0.6, $CHCl_3$);
HRMS (m/z): calcd for $C_{20}H_{32}O_3Na$, $[M+Na]^+$ 343.2244; found, 343.2244;
^{1}H NMR (400 MHz, $CDCl_3$): δ 9.75 (d, J = 2.2 Hz, 1H), 3.91 (d, J = 11.6 Hz, 1H), 3.80 (d, J = 11.6 Hz, 1H), 3.65 (tt, J = 10.7, 4.9 Hz, 1H), 2.32–2.22 (m, 2H), 2.16–2.04 (m, 1H), 1.98 (dt, J = 12.5, 3.4 Hz, 1H), 1.93–1.83 (m, 1H), 1.77–1.52 (m, 6H), 1.43–1.32 (m, 3H), 1.29–1.08 (m, 6H), 0.99–0.90 (m, 1H), 0.84 (dd, J = 13.8, 3.6 Hz, 1H), 0.78 (s, 3H), 0.77–0.70 (m, 1H).
^{13}C NMR (101 MHz, $CDCl_3$): δ 205.3, 71.1, 63.0, 60.8, 56.7, 54.7, 45.1, 45.0, 39.3, 39.1, 38.5, 35.6, 32.1, 32.0, 31.3, 28.2, 24.9, 22.6, 21.1, 14.3.

Supplementary Note 13: Stereonsteriod A (18, Supplementary Figure 80E**)** synthesis and characterization.
To a solution of Ph_3PCH_3Br (179 mg, 0.5 mmol, 10.0 equiv) in anhydrous THF (1.0 mL) was added nBuLi (1.5 M in hexane, 0.32 mL, 10.0 equiv) at 0 °C under argon. After stirring for 30 min, a solution of **17** (16 mg, 0.05 mmol, 1.0 equiv) in anhydrous THF (0.5 mL) was dropwise added to the mixture. Then, reaction

was quenched with saturated NH_4Cl solution after stirring for another 3 h, diluted with EtOAc (15 mL) and washed with H_2O (3 x 15 mL). The organic phase was dried over anhydrous Na_2SO_4, filtered and concentrated. The crude product was purified by silica gel column chromatography (20:1 CH_2Cl_2: MeOH) to afford **Stereonsteriod A** (15.4 mg, 0.043 mmol, 93%) as a colorless gum.

Physical state: colorless gum;

R_f = 0.4 (silica gel, 2:1 petroleum ether: EtOAc);

$[\alpha]_D^{25}$ = +3.6 (*c* =0.2, $CHCl_3$);

HRMS (*m*/*z*): calcd for $C_{21}H_{34}O_2Na$, $[M+Na]^+$ 341.2451; found, 341.2432;

^1H NMR (400 MHz, $CDCl_3$): δ 5.76 (ddd, *J* = 17.6, 10.9, 7.7 Hz, 1H), 4.98 (s, 1H), 4.95 (d, *J* = 7.7 Hz, 1H), 3.93 (d, *J* = 11.6 Hz, 1H), 3.80 (d, *J* = 11.7 Hz, 1H), 3.66 (tt, *J* = 10.8, 4.9 Hz, 1H), 2.27 (dt, *J* = 14.0, 3.9 Hz, 1H), 1.96–1.86 (m, 2H), 1.82–1.67 (m, 5H), 1.58–1.48 (m, 3H), 1.44–1.35 (m, 2H), 1.28–1.14 (m, 5H), 1.04–0.94 (m, 2H), 0.86–0.68 (m, 2H), 0.63 (s, 3H).

^{13}C NMR (101 MHz, $CDCl_3$): δ 140.0, 114.6, 71.2, 61.0, 56.1, 55.5, 55.1, 45.2, 43.8, 39.4, 38.7, 38.2, 36.2, 32.2, 32.1, 31.3, 28.3, 27.3, 24.9, 22.8, 13.4.

Supplementary Note 14: Acetylation of **Stereonsteriod A** (Supplementary Figure 80F)

To a solution of **Stereonsteriod A** (7.5 mg, 23.5 μmol, 1.0 equiv) in pyridine (1 mL) was added Ac_2O (3.6 mg, 35.3 μmol, 1.5 equiv) and DMAP (2.8 mg, 23.5 μmol, 1.0 equiv) at room temperature under an argon atmosphere. After stirred overnight, the mixture was quenched by H_2O (5 mL) and subsequently extracted with CH_2Cl_2 (3 x 10 mL). The organic phase was dried over anhydrous Na_2SO_4, filtered and concentrated. The crude product was purified by silica gel column chromatography (15:1 to 8:1 to 3:1 to 1:1 petroleum ether: EtOAc) to afford **Ceratosteriod D (19)** (1.5 mg, 3.7 μmol, 16%), **Ceratosteriod C (21)** (1.9 mg, 5.3 μmol, 22%), **Sclerosteriod A (20)** (1.4 mg, 3.9 μmol, 17%) and recovered **Stereonsteriod A (18)** (3.0 mg, 9.4μmol, 40%).

For **Sclerosteriod A (20):**

Physical state: colorless oil;

R_f = 0.4 (silica gel, 2:1 petroleum ether: EtOAc);

$[\alpha]_D^{25}$ = -22.7 (*c* = 0.1, $CHCl_3$);

HRMS (*m*/*z*): calcd for $C_{23}H_{36}O_3Na$, $[M+Na]^+$ 383.2557; found, 383.2559;

^1H NMR (600 MHz, $CDCl_3$): δ 5.74 (ddd, *J* = 16.4, 10.9, 7.7 Hz, 1H), 4.99–4.91 (d, *J* = 17.9 Hz, 2H), 4.34 (d, *J* = 12.1 Hz, 1H), 4.20 (d, *J* = 12.1 Hz, 1H), 3.64 (s, 1H), 2.25–2.17 (m, 1H), 2.06 (s, 3H), 1.98–1.91 (m, 1H), 1.88–1.82 (m, 1H), 1.80–1.60 (m, 6H), 1.55–1.45 (m, 2H), 1.40–1.14 (m, 7H), 1.04–0.83 (m, 4H), 0.79–0.71 (m, 1H), 0.57 (s, 3H).

^{13}C NMR (101 MHz, $CDCl_3$): δ 171.3, 139.8, 114.5, 70.9, 62.9, 55.9, 55.3, 54.6, 45.03, 43.6, 38.4, 38.0, 37.8, 35.9, 32.0, 31.8, 31.6, 28.2, 27.2, 24.7, 21.9, 21.2, 13.0.

For **Ceratosteriod C (21):**

Physical state: colorless gum;

R_f = 0.8 (silica gel, 2:1 petroleum ether: EtOAc);

$[\alpha]_D^{25}$ = +18.4 (c = 0.1, $CHCl_3$);

HRMS (m/z): calcd for $C_{23}H_{36}O_3Na$, $[M+Na]^+$ 383.2557; found, 383.2549;

^{1}H NMR (600 MHz, $CDCl_3$): δ 5.75 (ddd, J = 16.3, 11.1, 7.7 Hz, 1H), 5.03–4.90 (m, 2H), 4.74 (tt, J = 11.0, 5.0 Hz, 1H), 3.95 (d, J = 11.7 Hz, 1H), 3.81 (d, J = 11.5 Hz, 1H), 3.56 (s, 1H), 2.30–2.28 (m, 1H), 2.03 (s, 3H), 1.97–1.93 (m, 1H), 1.91–1.85 (m, 1H), 1.81–1.74 (m, 1H), 1.73–1.63 (m, 6H), 1.55–1.40 (m, 6H), 1.34–1.12 (m, 3H), 1.07–0.80 (m, 3H), 0.77–0.70 (m, 1H), 0.62 (s, 3H).

^{13}C NMR (101 MHz, $CDCl_3$): δ 170.9, 140.0, 114.7, 73.5, 61.0, 56.0, 55.5, 54.9, 45.0, 43.8, 39.4, 38.1, 36.2, 34.5, 32.1, 31.2, 29.9, 28.2, 28.1, 27.3, 24.9, 22.7, 21.6, 13.4.

For **Ceratosteriod D (19):**

Physical state: colorless gum;

R_f = 0.9 (silica gel, 2:1 petroleum ether: EtOAc);

$[\alpha]_D^{25}$ = -7.4 (c = 0.42, $CHCl_3$);

HRMS (m/z): calcd for $C_{25}H_{38}O_4Na$, $[M+Na]^+$ 425.2662; found, 425.2670;

^{1}H NMR (600 MHz, $CDCl_3$) δ 5.74 (ddd, J = 16.3, 11.0, 7.8, 1H), 5.01–4.92 (m, 2H), 4.72 (tt, J = 11.0, 4.9 Hz, 1H), 4.33 (d, J = 12.1 Hz, 1H), 4.23 (d, J = 12.1 Hz, 1H), 2.23 (d, J = 13.6 Hz, 1H), 2.06 (s, 3H), 2.02 (s, 3H), 1.96–1.92 (m, 1H), 1.85 (d, J = 12.1 Hz, 1H), 1.81–1.59 (m, 5H), 1.51–1.27 (m, 8H), 1.23–1.12 (m, 1H), 1.05–0.90 (m, 4H), 0.80–0.72 (m, 1H), 0.57 (s, 3H).

^{13}C NMR (101 MHz, $CDCl_3$): δ 171.4, 170.9, 139.9, 114.7, 73.2, 62.9, 56.0, 55.5, 54.6, 45.0, 43.8, 38.1, 37.9, 36.1, 34.3, 32.1, 31.7, 28.3, 27.7, 27.3, 24.9, 21.9, 21.6, 21.4, 13.2.

Supplementary Note 15: Stereonsteriod B (22, Supplementary Figure 80G**)** synthesis and characterization.

To a solution of **Ceratosteriod C** (2.0 equiv, 5.6 μmol, 1.0 equiv) in anhydrous CH_2Cl_2 (1 mL) was added DMP (5.0 mg, 11.1 μmol, 2.0 equiv) at r.t. under an argon atmosphere. The reaction was stirring for 3 h, then washed with saturated $NaHCO_3$ (3 x 5 mL) and brine (5 mL). The organic phase was dried over anhydrous Na_2SO_4, filtered and concentrated. The crude product was purified by silica gel column chromatography (15:1 petroleum ether: EtOAc) to afford **Stereonsteriod B** (2.0 mg, 100%) as a white solid.

Physical state: white solid;

R_f = 0.7 (silica gel, 5:1 petroleum ether: EtOAc);

$[\alpha]_D^{25}$ = +21.2 (c = 0.05, $CHCl_3$);

HRMS (m/z): calcd for $C_{23}H_{34}O_3Na$, $[M+Na]^+$ 381.2400; found, 381.2406;

^{1}H NMR (600 MHz, C**DCl_3):** δ 10.02 (s, 1H), 5.72 (ddd, J = 17.5, 10.5, 7.9 Hz, 1H), 5.00–4.91 (m, 2H), 4.70 (tt, J = 10.5, 4.7 Hz, 1H), 2.42 (d, J = 13.5 Hz, 1H), 2.00 (s, 3H), 1.98–1.85 (m, 4H), 1.83–1.75 (m, 2H), 1.73–1.65 (m, 2H),1.60–1.51 (m, 2H), 1.50–1.34 (m, 4H), 1.25–1.15 (m, 2H), 1.11–1.04 (m, 1H), 1.03–0.90 (m, 4H), 0.51 (s, 3H)

^{13}C NMR (101 MHz, $CDCl_3$): δ 208.3, 170.7, 139.5, 114.7, 72.7, 55.7, 55.3, 52.7, 51.6, 43.4, 43.3, 37.2, 37.0, 35.6, 31.9, 30.7, 28.4, 28.2, 27.1, 24.6, 21.4, 21.3, 12.8.

Supplementary Note 16: Compound **25 (**Supplementary Figure 81A) synthesis and characterization.
Argon was bubbled for 20 min through stirred Ac_2O (0.5 mL) containing compound **12** (6.9 mg, 19.9 μmol, 1.0 equiv) and NaI (12 mg, 80 μmol, 4.0 equiv). The mixture was cooled to 0 °C, and Me_3SiCl (9.0 mg, 80 μmol, 4.0 equiv) was added dropwise, then, the mixture was allowed to warm to 25 °C and stirring was continued for 4 h under an argon atmosphere. The reaction mixture was then poured into saturated aqueous $NaHCO_3$ (10 mL). After the mixture was stirred for 10 min, the product was extracted using EtOAc (3 × 15 mL). The combined extracts were washed with 10% aqueous $Na_2S_2O_3$ (2 × 10 mL), and brine (5 mL) and dried over Na_2SO_4. The solvent was removed to yield compound **24** which was used without further purification or characterization. Compound **24** was dissolved in EtOH (1 mL), and $NaBH_4$ (8 mg, one-time weight of starting material) was added. The solution was stirred under argon overnight (24 h). The reaction was quenched with acetone (1 mL) and stirred for another 10 min. Then, H_2O (1 mL) and $NaIO_4$ (21 mg, 0.1 mmol, 5.0 equiv) were added at rt. After stirred for 1 h, the mixture was diluted with EtOAc (10 mL) and washed with H_2O (10 mL) and brine (10 mL). The organic phase was dried over anhydrous Na_2SO_4, filtered and concentrated. The crude product was purified by silica gel column chromatography (2:1 petroleum ether: EtOAc) to afford **25** (4.4 mg, 13.8 μmol, 69% for two steps) as a white solid.
Physical state: white solid;
R_f = 0.6 (silica gel, 10:1 CH_2Cl_2: MeOH);
$[\alpha]_D^{25}$ = -27.8 (c = 0.3, $CHCl_3$);
HRMS (m/z): calcd for $C_{20}H_{31}O_3Na$, $[M+Na]^+$ 341.2087; found, 341.2065;
^1H NMR (400 MHz, $CDCl_3$) δ 9.77 (d, J = 2.2 Hz, 0.78H, C_{17}-Hα), 9.75 (d, J = 2.9 Hz, 0.22H, C_{17}-Hβ), 5.79–5.70 (m, 1H), 3.89–3.80 (m, 1H), 3.65–3.50 (m, 2H), 2.55–2.20 (m, 2H), 2.19–2.02 (m, 3H), 1.96–1.82 (m, 3H), 1.79–1.61 (m, 4H), 1.51 (d, J = 4.1 Hz, 1H), 1.47–1.35 (m, 2H), 1.34–1.25 (m, 2H), 1.73–1.07 (m, 1H), 1.04–0.81 (m, 5H).
^{13}C NMR (101 MHz, $CDCl_3$) δ 205.3, 135.7, 127.2, 71.5, 62.9, 62.9, 57.5, 50.5, 45.1, 42.4, 41.7, 38.7, 33.6, 33.0, 32.0, 31.2, 24.9, 21.3, 21.2, 14.3.

Supplementary Note 17: Reported **Sclerosteroid B (26,** Supplementary Figure 81B**)** synthesis and characterization.
To a solution of Ph_3PCH_3Br (42 mg, 0.12 mmol, 10.0 equiv) in anhydrous THF (1.0 mL) was added *n*BuLi (1.6 M in hexane, 73 μL, 10.0 equiv) at 0 °C under an argon atmosphere. After stirring for 30 min, a solution of **25** (4.2 mg, 11.7 μmol, 1.0 equiv) in anhydrous THF (0.2 mL) was dropwise added to the mixture. After stirred for another 2 h, Ac_2O (24 mg, 0.24 mmol, 20.0 equiv) and DMAP (1.5 mg, 0.012 mmol, 1.0 equiv) were added at rt under an argon atmosphere. After stirred for 5 h, the mixture was

diluted with EtOAc (10 mL) and washed with $NaHCO_3$ (3 x 10 mL). The organic phase was dried over anhydrous Na_2SO_4, filtered and concentrated. The crude product was purified by silica gel column chromatography (8:1 petroleum ether: EtOAc) to afford **26** (2.7 mg, 6.8 μmol, 60%) as a colorless oil.

Physical state: colorless oil;

R_f = 0.9 (silica gel, 2:1 petroleum ether: EtOAc);

$[\alpha]_D^{25}$ = -64.3 (*c* = 0.3, $CHCl_3$);

HRMS (*m*/*z*): calcd for $C_{25}H_{36}O_4Na$, $[M+Na]^+$ 423.2506; found, 423.2510;

^{1}H NMR (400 MHz, $CDCl_3$) *δ* 5.81–5.70 (m, 1H), 5.66–5.61 (m, 1H), 5.02–4.82 (m, 2H), 4.63 (tt, *J* = 10.9, 5.1 Hz, 1H), 4.53–4.44 m, 1H), 4.01–3.94 (m, 1H), 2.44–2.28 (m, 2H), 2.14–1.98 (d, *J* = 6.0 Hz, 9H), 1.94–1.67 (m, 5H), 1.65–1.59 (m, 1H), 1.55–1.39 (m, 3H), 1.23–1.05 (m, 3H), 1.04–0.91 (m, 2H), 0.82 (s, 0.65, C_{17}-Hβ), 0.62 (s, 2.59H, C_{17}-Hα).

^{13}C NMR (101 MHz, $CDCl_3$**)** *δ* 170.8, 170.6, 139.7, 134.6, 126.8, 114.7, 73.4, 64.6, 56.4, 55.2, 50.4, 43.5, 39.8, 38.1, 37.4, 33.5, 33.0, 31.4, 27.9, 27.2, 24.8, 21.4, 21.3, 21.2, 12.9.

Supplementary Note 18: Compound **27 (**Supplementary Figure 81C) synthesis and characterization.

To a solution of **12** (15 mg, 43 μmol, 1.0 equiv) in CH_2Cl_2/MeOH (1.5 mL, v: v = 3:2) was added $CeCl_3$ (32 mg, 0.13 mmol, 3.0 equiv) at -78 °C under an argon atmosphere. Then, a solution of $NaBH_4$ (3.3 mg, 87 μmol, 2.0 equiv) in EtOH (0.2 mL) was slowly added. The reaction mixture was allowed to warm to 0 °C for 20 min. Then, the reaction was quenched with acetone (1 mL) and stirred for 10 min. H_2O (1 mL) and $NaIO_4$ (46 mg, 0.22 mmol, 5.0 equiv) were added to the mixture at rt. After stirring for 1 h, the mixture was diluted with EtOAc (15 mL) and washed with H_2O (3 x 15 mL). The organic phase was dried over anhydrous Na_2SO_4, filtered and concentrated. The crude product was purified by silica gel column chromatography (2:1 petroleum ether: EtOAc) to afford **27** (10.4 mg, 32.7 μmol, 76%) as a white solid.

Physical state: white solid;

$\boldsymbol{R_f}$ = 0.6 (silica gel, 10:1 CH_2Cl_2: MeOH);

$\boldsymbol{[\alpha]_D^{25}}$ = +37.7 (*c* = 0.3, $CHCl_3$);

^{1}H NMR (400 MHz, $CDCl_3$): *δ* 9.76 (d, *J* = 2.1 Hz, 0.91 H), 9.73 (d, *J* = 2.9 Hz, 0.07 H), 5.73–5.67 (m, 1H), 4.19–4.13 (m, 1H), 3.97 (dd, *J* = 10.7, 1.2 Hz, 1H), 3.64 (d, *J* = 10.7 Hz, 1H), 2.28 (td, *J* = 9.0, 2.1 Hz, 1H), 2.20–2.06 (m, 3H), 2.02–1.97 (m, 1H), 1.97–1.89 (m, 1H), 1.84–1.63 (m, 5H), 1.60–1.51 (m, 1H), 1.45–1.38 (m, 1H), 1.38–1.24 (m, 3H), 1.14–1.04 (m, 1H), 1.02–0.91 (m, 2H), 0.77 (s, 3H).

^{13}C NMR (101 MHz, $CDCl_3$): *δ* 205.0, 142.5, 128.6, 66.8, 65.8, 62.8, 56.3, 53.8, 45.0, 42.7, 38.8, 36.1, 33.0, 32.6, 31.9, 29.9, 24.8, 21.1, 21.1, 14.1.

Supplementary Note 19: Sclerosteriod B (28, Supplementary Figure 81D**)** synthesis and characterization.

To a solution of Ph_3PCH_3Br (44 mg, 0.124 mmol, 10.0 equiv) in anhydrous THF (1.0 mL) was added *n*BuLi (2.5 M in hexane, 50 μL, 10.0 equiv) at 0 °C under an argon atmosphere. After stirring for 30 min,

a solution of **27** (4.0 mg, 12.4 μmol, 1.0 equiv) in anhydrous THF (0.2 mL) was dropwise added to the mixture. After stirred for another 3 h, Ac_2O (24 mg, 0.24 mmol, 20.0 equiv) and DMAP (1.5 mg, 0.012 mmol, 1.0 equiv) were added at rt under an argon atmosphere. After stirred for 2 h, the mixture was diluted with EtOAc (10 mL) and washed with $NaHCO_3$ (3 x 10 mL). The organic phase was dried over anhydrous Na_2SO_4, filtered and concentrated. The crude product was purified by silica gel column chromatography (8:1 petroleum ether: EtOAc) to afford **Sclerosteriod B** (3.5 mg, 8.8 μmol, 70%) as a colorless oil.

Physical state: colorless oil;

R_f = 0.7 (silica gel, 1:2 CH_2Cl_2: EtOAc);

$[\alpha]_D^{25}$ = +14.7 (c = 0.3, $CHCl_3$);

HRMS (*m*/*z*): calcd for $C_{25}H_{36}O_4Na$, $[M+Na]^+$ 423.2506; found, 423.2510;

^{1}H NMR (400 MHz, $CDCl_3$): δ 5.74 (ddd, J = 16.7, 10.6, 7.7 Hz, 1H), 5.49–5.43 (m, 1H), 5.25–5.17 (m, 1H), 5.00–4.91 (m, 2H), 4.48 (d, J = 11.2 Hz, 1H), 4.13 (d, J = 11.3 Hz, 1H), 2.25–2.15 (m, 1H), 2.11–2.02 (m, 8H), 1.98–1.89 (m, 2H), 1.83–1.60 (m, 6H), 1.56–1.49 (m, 3H), 1.46–1.30 (m, 2H), 1.20–1.13 (m, 1H), 1.00–0.91 (m, 2H), 0.90–0.87 (m, 1H), 0.60 (s, 3H).

^{13}C NMR (101 MHz, $CDCl_3$): δ 171.2, 171.2, 171.2, 145.1, 139.8, 122.4, 114.9, 77.5, 70.1, 67.0, 55.8, 55.3, 54.6, 43.6, 40.7, 37.8, 36.5, 33.2, 32.5, 31.7, 29.9, 27.3, 25.5, 24.8, 21.6, 21.3, 21.2, 13.1.

Supplementary References

1. Renata, H., Zhou, Q., Dünstl, G., Felding, J., Merchant, R. R., Yeh, C. H., Baran, P. S.. Development of a concise synthesis of ouabagenin and hydroxylated corticosteroid analogues. *J. Am. Chem. Soc.*, **137**, 1330-1340 (2015).

2. Kirk, D. N., Boon L. Y. New syntheses of 19, 21-dihydroxypregn-4-ene-3, 20-dione, 21-hydroxy-19-norpregn-4-ene-3, 20-dione, and 11β, 19, 21-trihydroxypregn-4-ene-3, 20-dione. *J. Chem. Soc., Perkin Trans. 1* **0**, 2945-2951 (1983).

3. Elks, Joseph, ed. The dictionary of drugs: chemical data: chemical data, structures and bibliographies. Springer, 2014.

4. Terasawa, T.; Okada, T., Convenient preparative routes to 19-hydroxy, 19-oxo-, 19-oic-, and 19-nor-deoxycorticosterone. *Tetrahedron* **42**, 537-545 (1986).

5. Huang, J.; Wang, H., Method for preparing 19-demethyl-4-androstenedione, CN 106928301 A, 2017-07-17.

6. Hofmann, K. & Stoffel W. TMbase - A database of membrane spanning proteins segments. *Biol. Chem.* Hoppe-Seyler **374**,166 (1993).

7. Wang, S.-K.; Dai, C.-F.; Duh, C.-Y., Cytotoxic Pregnane Steroids from the Formosan Soft Coral *Stereonephthya crystalliana*. *J. Nat. Prod*. **69**, 103-106 (2006).

8. Ferraboschi P, De Mieri M, Ragonesi L. Lipase-catalyzed preparation of corticosteroid 17α-esters endowed with antiandrogenic activity. *Tetrahedron Lett*. **49**, 4610-4612 (2008).

9. Fang, H.-Y.; Liaw, C.-C.; Chao, C.-H.; Wen, Z.-H.; Wu, Y.-C.; Hsu, C.-H.; Dai, C.-F.; Sheu, J.-H., Bioactive pregnane-type steroids from the soft coral Scleronephthya gracillimum. *Tetrahedron* **68**, 9694-9700 (2012).

10. Lu, C.-K.; Wang, S.-K.; Duh, C.-Y., New pregnane steroids from Formosan red alga Ceratodictyon spongiosum and symbiotic sponge Sigmadocia symbiotica. *Bull Chem Soc Jpn.* **84**, 943-946 (2011).

11. Clark, T. A.; Chong, R.; Maddox, I. S., An investigation into the 19-hydroxylation of androstenedione, cortexolone and progesterone by selected fungi. *Appl. Microbiol. Biotechnol.* **21**, 132-134 (1985).

12. Wang, Y Fujii, T.; Yamaoka, H.; Gomi, K.; Kitamoto, A.; Kumagai, C., Cloning and nucleotide sequence of the ribonuclease T1 gene (rntA) from Aspergillus oryzae and its expression in *Saccharomyces cerevisiae* and *Aspergillus oryzae*. *Biosci. Biotech. Biochem.* **59**, 1869-1874 (1995).

13. Jin, F. J.; Maruyama, J.-i.; Juvvadi, P. R.; Arioka, M.; Kitamoto, K., Development of a novel quadruple auxotrophic host transformation system by argB gene disruption using adeA gene and exploiting adenine auxotrophy in *Aspergillus oryzae*. *FEMS Microbiol. Lett.* **239**, 79-85 (2004).

14. Jin, F. J.; Maruyama, J.-i.; Juvvadi, P. R.; Arioka, M.; Kitamoto, K., Adenine auxotrophic mutants of *Aspergillus oryzae*: development of a novel transformation system with triple auxotrophic hosts. *Biosci. Biotech. Biochem*. **68**, 656-662 (2004).

15. Sambrook, J.; Russell, D. W., *Molecular Cloning: A Laboratory Manual, 3rd ed.* Cold Spring Harbor Laboratory Press, New York: 2001.
