# Supplementary material for: A biocatalytic hydroxylation-enabled unified approach to C19-hydroxylated steroids
Source: Nat Commun. 2019 Jul 29;10:3378. doi: 10.1038/s41467-019-11344-0 (PMC6662754; doi:10.1038/s41467-019-11344-0)
Supplement: Supplementary file 3 — Reporting Summary [file 41467_2019_11344_MOESM3_ESM.pdf]

nature research

Corresponding author(s): Xudong Qu

Last updated by author(s): Jul 10, 2019

Reporting Summary

Nature Research wishes to improve the reproducibility of the work that we publish. This form provides structure for consistency and transparency in reporting. For further information on Nature Research policies, see Authors & Referees and the Editorial Policy Checklist.

Statistics

For all statistical analyses, confirm that the following items are present in the figure legend, table legend, main text, or Methods section.

n/a	Confirmed	
☐	☒	The exact sample size (*n*) for each experimental group/condition, given as a discrete number and unit of measurement
☐	☒	A statement on whether measurements were taken from distinct samples or whether the same sample was measured repeatedly
☐	☒	The statistical test(s) used AND whether they are one- or two-sided<br>*Only common tests should be described solely by name; describe more complex techniques in the Methods section.*
☒	☐	A description of all covariates tested
☒	☐	A description of any assumptions or corrections, such as tests of normality and adjustment for multiple comparisons
☒	☐	A full description of the statistical parameters including central tendency (e.g. means) or other basic estimates (e.g. regression coefficient) AND variation (e.g. standard deviation) or associated estimates of uncertainty (e.g. confidence intervals)
☒	☐	For null hypothesis testing, the test statistic (e.g. *F*, *t*, *r*) with confidence intervals, effect sizes, degrees of freedom and *P* value noted<br>*Give P values as exact values whenever suitable.*
☒	☐	For Bayesian analysis, information on the choice of priors and Markov chain Monte Carlo settings
☒	☐	For hierarchical and complex designs, identification of the appropriate level for tests and full reporting of outcomes
☒	☐	Estimates of effect sizes (e.g. Cohen's *d*, Pearson's *r*), indicating how they were calculated

Our web collection on statistics for biologists contains articles on many of the points above.

Software and code

Policy information about availability of computer code

Data collection	Labsolution (Shimadzu, Inc, JPN)for HPLC data<br>TopSpin(Bruker, Inc,DE) for NMR data<br>Xcalibur (Themo Fisher Scientific ,Inc,USA) for LC-MS(<br>Crysalispro （Bruker, Inc, DE） for Crystal data<br>CytoFLEX flow cytometry (Beckman Coulter, Inc,USA) for Flow cytometry data
Data analysis	Labsolution (Shimadzu, Inc, JPN)for HPLC data<br>MestReNova for NMR data<br>Xreport (Themo Fisher Scientific ,Inc,USA) for LC-MS<br>Crysalispro （Bruker, Inc, DE） for Crystal data<br>FlowJofus for Flow cytometry data

For manuscripts utilizing custom algorithms or software that are central to the research but not yet described in published literature, software must be made available to editors/reviewers. We strongly encourage code deposition in a community repository (e.g. GitHub). See the Nature Research guidelines for submitting code & software for further information.

Data

Policy information about availability of data

All manuscripts must include a data availability statement. This statement should provide the following information, where applicable:

- Accession codes, unique identifiers, or web links for publicly available datasets
- A list of figures that have associated raw data
- A description of any restrictions on data availability

Accession number of P450 genes, CCDC deposition number of compound crystal structure and Source Data file

Field-specific reporting

Please select the one below that is the best fit for your research. If you are not sure, read the appropriate sections before making your selection.

☒ Life sciences ☐ Behavioural & social sciences ☐ Ecological, evolutionary & environmental sciences

For a reference copy of the document with all sections, see nature.com/documents/nr-reporting-summary-flat.pdf

Life sciences study design

All studies must disclose on these points even when the disclosure is negative.

Sample size	For the data in Supplementary Figure 2-4, two paralleled experiments were performed to achieve the results. For the data in Supplementary Figure 12, triplicate experiments were conducted for each group. As these experiments are very simple, duplicate or triplicate experiments are adequate for achieve the reliable data.
Data exclusions	No data was excluded.
Replication	For Figure 2-4, we ensure the same inoculation, the same fermentation conditions, the same medium, the same treatment methods, etc. For Figure 12, we ensure the same cell culture conditions and methods. In short, all conditions have been kept same.
Randomization	The order of testing and analysis of each samples are at random.
Blinding	The blingding was not possible for our experiments, each group of experiments was labeled accordingly. But all the experimental operations and conditions were kept same to ensure the accuracy of the experiments.

Reporting for specific materials, systems and methods

We require information from authors about some types of materials, experimental systems and methods used in many studies. Here, indicate whether each material, system or method listed is relevant to your study. If you are not sure if a list item applies to your research, read the appropriate section before selecting a response.

Materials & experimental systems

n/a	Involved in the study	
☒	☐	Antibodies
☐	☒	Eukaryotic cell lines
☒	☐	Palaeontology
☒	☐	Animals and other organisms
☒	☐	Human research participants
☒	☐	Clinical data

Methods

n/a	Involved in the study	
☒	☐	ChIP-seq
☐	☒	Flow cytometry
☒	☐	MRI-based neuroimaging

Eukaryotic cell lines

Policy information about cell lines

Cell line source(s)	The cell line is the HT-29 (ATCC® HTB-38)
Authentication	Pre-genomic DNA was extracted from HT-29 cells using Purelink®Genomic DNA Mini Kit and amplified by using PowerPlex®18D system kit(Promega DC1802,USA).The DNA was detected by ABI3500 Genetic Analyzer (Life3500, USA).
Mycoplasma contamination	One-step thermostatic Mycoplasma test kit (ShanghaiYise Medical Technology Co.,Ltd) was used to detect cell supernatant, and the results were negative.
Commonly misidentified lines (See ICLAC register)	There are no commonly misindentified cell lines used in this study

Flow Cytometry

Plots

Confirm that:

☒ The axis labels state the marker and fluorochrome used (e.g. CD4-FITC).

☒ The axis scales are clearly visible. Include numbers along axes only for bottom left plot of group (a 'group' is an analysis of identical markers).

☒ All plots are contour plots with outliers or pseudocolor plots.

☒ A numerical value for number of cells or percentage (with statistics) is provided.

Methodology

Sample preparation	1. HT-29 cells in logarithmic phase were evenly laid in 6-well plates with 3*10^ 5 cells per well. 2. The compounds were completely dissolved in DMSO to an appropriate concentration. were added the next day, blank control was 1% DMSO, and three biological replications were performed. 3. Cells were digested with 0.25% trypsin without EDTA. Cells were collected after termination of digestion and centrifuged at 1500rpm for 5 minutes. Supernatant was removed, and PBS was suspended again.4. Cells were washed twice with PBS, 1500rpm for 5 minutes.3. According to the operation instructions of Annexin V-FITC/PI cell apoptosis detection kit.The cells were suspended by adding 500 mL Binding Buffer. Add 5 mu L Annexin V-FITC, mix and add 5 mu L PI. Room temperature photoavoidance reaction for 5-15 minutes (with negative control, i.e. normal cells without Annexin V-FITC and PI;4. Flow cytometry was used for detection.
Instrument	CytoFLEX flow cytometry (Beckman Coulter, Inc,USA)
Software	the software is the FlowJofus
Cell population abundance	Each pannel respectively stands for the distribution of normal cells (FITC-PI-), the early apoptotic cells (FITC+PI-), the late apoptotic cells (FITC+PI+), and the necrotic cells (FITC-PI+). Triplicate experiments were conducted for each group. For the control group: normal cells (FITC-PI-) are as follows 91.11%, 90.01%, 91.68%; the early apoptotic cells (FITC+PI-) are as follows 4.28%, 4.13%,3.83%; the late apoptotic cells (FITC+PI+) are as follows: 2.51%, 2.66%,2.24%; the necrotic cells (FITC-PI+):2.10%, 3.20%,2.25%. For the compound 28: normal cells (FITC-PI-) are as follows 74.66%, 71.05%, 70.08%; the early apoptotic cells (FITC +PI-) are as follows 11.72%, 12.39%, 13.14%; the late apoptotic cells (FITC+PI+) are as follows: 12.00%, 14.59%, 14.82%; the necrotic cells (FITC-PI+):1.61%, 1.97%,1.96%.<br>Compound samples were purified by silica gel column separation method. The purity was identified by NMR. The results of NMR data is provided in the Supplementary Information
Gating strategy	the gating strategy is provided in the Supplementary Information

☒ Tick this box to confirm that a figure exemplifying the gating strategy is provided in the Supplementary Information.
